# Large Amplitude Quasi-Periodic Traveling Waves in Two Dimensional Forced Rotating Fluids

**DOI:** 10.1007/s00220-025-05247-z

**Published:** 2025-02-20

**Authors:** Roberta Bianchini, Luca Franzoi, Riccardo Montalto, Shulamit Terracina

**Affiliations:** 1 Consiglio Nazionale Delle Ricerche, 00185 Roma, Italy; 2 Dipartimento di Matematica “Federigo Enriques”, Università degli Studi di Milano, Via Cesare Saldini 50, 20133 Milano, Italy; 3 SISSA, Scuola Internazionale di Studi Avanzati, Via Bonomea 265, 34136 Trieste, Italy

## Abstract

We establish the existence of quasi-periodic traveling wave solutions for the $\beta$-plane equation on ${\mathbb {T}}^2$ with a large quasi-periodic traveling wave external force. These solutions exhibit large sizes, which depend on the frequency of oscillations of the external force. Due to the presence of small divisors, the proof relies on a nonlinear Nash-Moser scheme tailored to construct nonlinear waves of large size. To our knowledge, this is the first instance of constructing quasi-periodic solutions for a quasilinear PDE in dimensions greater than one, with a 1-smoothing dispersion relation that is highly degenerate - indicating an infinite-dimensional kernel for the linear principal operator. This degeneracy challenge is overcome by preserving the traveling-wave structure, the conservation of momentum and by implementing normal form methods for the linearized system with sublinear dispersion relation in higher space dimension.

## Introduction

Incompressible rotating fluids in three dimensions are described by the Euler-Coriolis equations, which, on the three dimensional torus ${\mathbb {T}}^3 = ({\mathbb {R}}/2\pi {\mathbb {Z}})^3$, read as

1.1 $$\begin{aligned} \begin{aligned}&\partial _{t} {{\textbf {u}}}+ ({{\textbf {u}}}\cdot \nabla ) {{\textbf {u}}}+ {\mathfrak {f}}\, \overrightarrow{e_3} \wedge {{\textbf {u}}}+ \nabla P = \textbf{F}, \\&\quad \nabla \cdot {{\textbf {u}}}=0, \end{aligned} \end{aligned}$$

where ${{\textbf {u}}}(t, x): {\mathbb {R}}\times {\mathbb {T}}^3 \rightarrow {\mathbb {R}}^3$ is the divergence-free velocity field, $P(t, x): {\mathbb {R}}\times {\mathbb {T}}^3 \rightarrow {\mathbb {R}}$ is the scalar pressure, ${\mathfrak {f}}={\mathfrak {f}}(x_2)$ is a scalar function, $\overrightarrow{e_3}=(0,0,1)^\top$ is the rotation vector, and $\textbf{F}(t,x):{\mathbb {R}}\times {\mathbb {T}}^3 \rightarrow {\mathbb {R}}^3$ is an external force. The term ${\mathfrak {f}}\,\overrightarrow{e_3}\wedge {{\textbf {u}}}$ is usually referred as the Coriolis force. It is customary in geophysical fluid dynamics to simplify the model introducing some approximations. First, noting that $\overrightarrow{e_3}\wedge {{\textbf {u}}}= (-u_2,u_1,0)^\top := ({{\textbf {u}}}_h^\perp , 0)^\top$, according to the notation ${{\textbf {u}}}=(u_1,u_2,u_3)=: ({{\textbf {u}}}_h,u_3)$, we assume a trivial dependence with respect to the third direction, namely $\partial _{x_3} {{\textbf {u}}}=0$ and $\partial _{x_3}\textbf{F}= 0$. As part of the standard elliptic constraint $\nabla \cdot \big (({{\textbf {u}}}\cdot \nabla ){{\textbf {u}}}+{\mathfrak {f}}\, \overrightarrow{e_3} \wedge {{\textbf {u}}}- \textbf{F}\big ) + \Delta P =0$ on the pressure, we also assume $\partial _{x_3} P =0$. This way, setting

$$\begin{aligned} \nabla =(\partial _{x_1},\partial _{x_2},\partial _{x_3})^\top =: (\nabla _{h},\partial _{x_3})^\top , \quad \textbf{F}= ({\mathtt F}_{1},{\mathtt F}_{2},{\mathtt F}_{3})^\top =: (\textbf{F}_{h},{\mathtt F}_{3})^\top , \end{aligned}$$

we are led to the reduced two dimensional problem

1.2 $$\begin{aligned} \begin{aligned}&\partial _{t} {{\textbf {u}}}_h + ({{\textbf {u}}}_h \cdot \nabla _h) {{\textbf {u}}}_h + {\mathfrak {f}}\, {{\textbf {u}}}_h^\perp + \nabla _{h} P = \textbf{F}_{h} , \\&\quad \nabla _{h} \cdot {{\textbf {u}}}_h=0. \end{aligned} \end{aligned}$$

A solution to the original three dimensional problem (1.1) is then recovered by solving the linear transport equation $\partial _{t} u_3 + {{\textbf {u}}}_h \cdot \nabla _{h} u_3 = {\mathtt F}_{3}$.

Next, we introduce the $\beta$-*plane approximation* by choosing

$$\begin{aligned} {\mathfrak {f}}(x_2)=\beta x_2, \end{aligned}$$

where $\beta \in {\mathbb {R}}\setminus \{0\}$ is a non-trivial constant. Note that we choose ${\mathfrak {f}}$ to be a *linear* function, and the vertical dynamics are considered trivial, i.e. $\partial _{x_3}{{\textbf {u}}}= 0$ as above. For further details, we refer to works such as McWilliams [40], Pedlosky [42], Chemin, Desjardins, Gallagher and Grenier [15], Gallagher and Saint-Raymond [29], and Pusateri and Widmayer [43]. The constant $\beta \in {\mathbb {R}}\setminus \{0\}$ represents the speed of rotation of the frame system around the rotation vector $\overrightarrow{e_3}$. Throughout this work, we treat $\beta$ as a fixed constant.

Introducing the scalar vorticity (i.e. the third component of the vorticity field $\nabla \wedge {{\textbf {u}}}$)

$$\begin{aligned} v:= \textrm{curl} \, {{\textbf {u}}}_h := \partial _{x_1} u_2 - \partial _{x_2} u_1, \end{aligned}$$

and applying the $\textrm{curl}$ operator to (1.2), yields the scalar equation

1.3 $$\begin{aligned} \partial _t v + {{\textbf {u}}}_h \cdot \nabla _{h} v + \beta \, {\mathtt L}v = \textrm{curl}\, \textbf{F}_{h} ,\quad \textbf{u}_h=\nabla _{h}^\perp (-\Delta )^{-1} v , \end{aligned}$$

which is the so-called $\beta$-*plane equation*, where we have, under the incompressibility condition $\nabla _{h}\cdot {{\textbf {u}}}_h = 0$,

$$\begin{aligned} \textrm{curl}( {\mathfrak {f}}\,{{\textbf {u}}}_h^\perp )= \partial _{x_1}(\beta x_2 u_1) - \partial _{x_2}(-\beta x_2 u_2) = \beta u_2 = \beta \, {\mathtt L}v, \end{aligned}$$

and the operator

1.4 $$\begin{aligned} {{\mathtt L}:=- (-\Delta )^{-1} \partial _{x_1}}, \end{aligned}$$

namely the second component of $\nabla _{h}^\perp (-\Delta )^{-1}$ with $\nabla _{h}^\perp = (\partial _{x_2},-\partial _{x_1})^\top$, acts on any function $h(x):{\mathbb {T}}^2\rightarrow {\mathbb {R}}$ with zero average as the Fourier multiplier

1.5 $$\begin{aligned} {\mathtt L}h(x):= \sum _{\xi \in {\mathbb {Z}}^2\setminus \{0\}} \textrm{i}\,{\mathtt L}(\xi ) {{\widehat{h}}}(\xi ) e^{\textrm{i}\,\xi \cdot x}, \quad { {\mathtt L}(\xi ):=- \frac{\xi _{1}}{|\xi |^2} }\quad \forall \,\xi =(\xi _{1},\xi _{2})^\top \in {\mathbb {Z}}^2\setminus \{0\}.\nonumber \\ \end{aligned}$$

### Remark 1.1

The sign in front of $\beta$ in equation (1.3) may be different compared to previous works in literature (for instance, see [21, 41, 43]). This discrepancy comes from how the operator ${\mathtt L}$ in (1.4)-(1.5) is defined, where a change of sign in front of the Laplacian appears depending on the sign convention for $\nabla _{h}^\perp$.

Approximating models for rotating fluids in two and three dimensions are commonly used in oceanography and geophysical fluid dynamics. An interesting regime that is frequently studied is when the fluid is rapidly rotating, namely when $|\beta | \gg 1$ in the $\beta$-plane approximation, even in the viscous case. In a series of works, Babin, Mahalov and Nicolaenko [2–4] proved regularity and integrability properties of solutions in 3D resonant tori. Dalibard and Saint-Raymond [19] devoted their analysis of the long-time behavior of solutions The singular limit in the vanishing Rossby number $\text {Ro} \sim \beta ^{-1}$ was investigated by Charve [14] and Dutrifoy [20]. The long-time behavior of solutions with high-speed rotation was studied by Koh, Lee and Takada [38] and Takada [46] by means of Strichartz estimates, by Dalibard [18] under the effect of random forcing and by Angullo-Castillo and Ferreira [1] in Besov spaces. The relation between the speed of rotation and the lifespan of solutions was investigated by Ghoul, Ibrahim, Lin and Titi [30] for the three dimensional primitive equations.

Independently of the speed of rotation, the global well-posedness of solutions was studied for the $\beta$-plane equation by Elgindi and Widmayer [21] and Pusateri and Widmayer [43]. The global well-posedness for the Euler-Coriolis equations was studied by Guo, Huang, Pausader and Widmayer [32] and Guo, Pausader and Widmayer [33] with axisymmetric initial data, and, very recently, by Ren and Tian [44] with general non-axisymmetric initial data. For the $\beta$-plane equation, we mention also the works of Wei, Zhang and Zhu [48] on the linear inviscid damping around shear flows, and of Wang, Zhang and Zhu [47] on the existence of non-shear solutions close to the Couette flow depending on the size of the rotational speed $\beta$.

### Main result

Led by the physical model discussed above, in this paper we focus our attention on the effect of a *large* forcing term of the form

$$\begin{aligned} \textrm{curl}\, \textbf{F}_{h} (t,x) = \lambda ^{\alpha } f(\lambda \,\omega t,x) \end{aligned}$$

where the parameter $\lambda \gg 1$ is large, $\alpha >0$ is a positive exponent and, given $\nu \in {\mathbb {N}}$, $\nu \geqslant 2$, the function $f({\varphi },x)$ is *quasi-periodic in time* with frequency vector $\lambda \,\omega \in {\mathbb {R}}^\nu \setminus \{0\}$, evaluated at the linear flow $t\mapsto f({\varphi },x)|_{{\varphi }= \lambda \, \omega t}$. We shall assume that $f \in {{\mathcal {C}}}^\infty ({\mathbb {T}}^\nu \times {\mathbb {T}}^2)$, , has zero average in *x*, that is

1.6 $$\begin{aligned} \int _{{\mathbb {T}}^2} f({\varphi }, x)\, \,\textrm{d}{x} = 0, \quad \forall {\varphi }\in {\mathbb {T}}^\nu . \end{aligned}$$

The parameter $\alpha >0$ will be fixed later in order to construct solutions of large amplitude.

Therefore, with a minor change in the notation, from now on we work with the following equation, on the two dimensional torus ${\mathbb {T}}^2$,

1.7 $$\begin{aligned} \partial _{t} v+ \beta \, {\mathtt L}v + u \cdot \nabla v = \lambda ^{\alpha } f(\lambda \,\omega t,x) , \quad t\in {\mathbb {R}}, \ x \in {\mathbb {T}}^2, \end{aligned}$$

where the two dimensional velocity field $u:{\mathbb {R}}\times {\mathbb {T}}^2 \rightarrow {\mathbb {R}}^2$ is determined from the scalar vorticity $v: {\mathbb {R}}\times {\mathbb {T}}^2 \rightarrow {\mathbb {R}}$ by the classical Biot-Savart law

1.8 $$\begin{aligned} u(t,x):= {\mathfrak {B}}[v (t,x)]:= \nabla ^\perp (-\Delta )^{-1} v(t,x) = \sum _{\xi \in {\mathbb {Z}}^2\setminus \{0\}} {\frac{\textrm{i}(\xi _{2},-\xi _{1})^\top }{|\xi |^2} }{{\widehat{v}}}(t,\xi ) e^{\textrm{i}\, \xi \cdot x}.\nonumber \\ \end{aligned}$$

With equation (1.7), we aim to study how the presence of a forcing term, *of substantial magnitude* and *highly oscillating in time*, affects the dynamics in the $\beta$-plane approximation *without relying on a perturbative regime*. Additionally, we seek to determine whether the quasi-periodic structure of the external force produces a time quasi-periodic solution of (1.7), *with substantial size* and *rapid* oscillations, specifically with a frequency vector of oscillation $\lambda \,\omega \in {\mathbb {R}}^\nu {\setminus }\{0\}$ of significant magnitude. The interaction between the size $\lambda$ of the vector $\lambda \,\omega$ and the size $\lambda ^\alpha$ of the forcing term will be discussed later.

In particular, we search for *quasi-periodic traveling wave* solutions, according to the following definition.

#### Definition 1.2

*(Quasi-periodic traveling wave).* Let $\nu \in {\mathbb {N}}$. We say that a function $v:{\mathbb {R}}\times {\mathbb {T}}^2\rightarrow {\mathbb {R}}$ is a *quasi-periodic traveling wave* with irrational frequency vector $\omega = (\omega _{1},...,\omega _{\nu })\in {\mathbb {R}}^{\nu }{\setminus }\{0\}$, that is $\omega \cdot \ell \ne 0$ for any $\ell \in {\mathbb {Z}}^\nu \setminus \{0\}$, and “wave vectors” ${\overline{\jmath }}_{1},...,{\overline{\jmath }}_{\nu }\in {\mathbb {Z}}^2$ if there exists a function $\breve{v}:{\mathbb {T}}^\nu \rightarrow {\mathbb {R}}$ such that

$$\begin{aligned} v(t,x) = \breve{v} (\omega _{1}t- \overline{\jmath }_1\cdot x,...,\omega _{\nu }t-\overline{\jmath }_{\nu }\cdot x) = \breve{v}(\omega t- \pi ( x)) , \quad \forall (t,x) \in {\mathbb {R}}\times {\mathbb {T}}^2, \end{aligned}$$

where $\pi :{\mathbb {R}}^2 \rightarrow {\mathbb {R}}^\nu$ is the linear map $x \mapsto \pi ( x):= (\overline{\jmath }_{1}\cdot x,...,\overline{\jmath }_{\nu }\cdot x)$. We also denote by $\pi ^\top$ the transpose of the map $\pi$.

We fix once and for all the $\nu$ vectors ${\overline{\jmath }}_{1},...,{\overline{\jmath }}_{\nu }\in {\mathbb {Z}}^2$ such that $\textrm{dim}\,\textrm{span}\{\overline{\jmath }_{1},...,\overline{\jmath }_{\nu }\} = 2$, in order to avoid trivial cases. Therefore, assuming that the forcing term $f(\lambda \,\omega t, x)$ is a quasi-periodic traveling function according to Definition 1.2, for most values of the parameter $\omega \in {\mathbb {R}}^\nu \setminus \{0\}$, we search for quasi-periodic traveling wave solutions $v({\varphi },x)|_{{\varphi }= \lambda \,\omega t}$ with *large* frequency vector $\lambda \,\omega \in {\mathbb {R}}^{\nu }$ (with a slight abuse of notation between *v*(*t*, *x*) and $v(\lambda \,\omega t,x)$) of the equations (1.7)-(1.8), which we rewrite as

1.9 $$\begin{aligned} \lambda \,\omega \cdot \partial _{{\varphi }} v + \beta \,{\mathtt L}v + u \cdot \nabla v = \lambda ^{\alpha } f(\lambda \, \omega t,x) , \quad u = {\mathfrak {B}}[v] = \nabla ^\perp (-\Delta )^{-1} v. \end{aligned}$$

We look for solutions $v \in H^s_0({\mathbb {T}}^\nu \times {\mathbb {T}}^2)$, for $s \gg 1$ large enough, where

1.10 $$\begin{aligned} \begin{aligned} H^s({\mathbb {T}}^\nu \times {\mathbb {T}}^2)&:= \Big \{ u({\varphi }, x) = \sum _{(\ell , j) \in {\mathbb {Z}}^\nu \times {\mathbb {Z}}^2 } {\widehat{u}}(\ell , j) e^{\textrm{i}(\ell \cdot {\varphi }+ j \cdot x)}: \Vert u \Vert _s^2 \\&:= \sum _{(\ell , j) \in {\mathbb {Z}}^\nu \times {\mathbb {Z}}^2} \langle \ell , j \rangle ^{2 s} |{\widehat{u}}(\ell , j)|^2 < + \infty \Big \}, \\ H^s_0({\mathbb {T}}^\nu \times {\mathbb {T}}^2)&:= \Big \{ u \in H^s({\mathbb {T}}^\nu \times {\mathbb {T}}^2): \int _{{\mathbb {T}}^2} u({\varphi }, x) \, d x = 0 \Big \}, \\ \langle \ell , j \rangle&:= \max \{ 1, |\ell |, |j| \}. \end{aligned} \end{aligned}$$

Let $\Omega \subset {\mathbb {R}}^\nu$ be the reference compact set

1.11 $$\begin{aligned} \Omega := \{\omega \in {\mathbb {R}}^\nu : \, 1 \leqslant |\omega | \leqslant 2 \}. \end{aligned}$$

The main theorem of this paper is the following.

#### Theorem 1.3

Let $\beta \in {\mathbb {R}}\setminus \{0\}$, $\alpha \in (1,2)$ and $\nu \in {\mathbb {N}}$, $\nu \geqslant 2$, be fixed. Let $f \in {{\mathcal {C}}}^\infty ({\mathbb {T}}^\nu \times {\mathbb {T}}^2, {\mathbb {R}})$ be a quasi-periodic traveling wave (see Definition 1.2) satisfying (1.6). There exists ${\overline{s}} = {\overline{s}}(\nu , \alpha ) > 0$ such that, for any $s \geqslant {\overline{s}}(\nu , \alpha )$, there exist $\lambda _0 = \lambda _0(f, s, \nu , \alpha , \beta ) \gg 1$ large enough and constants $C_1, C_2>0$, with $C_i = C_i(f, s, \nu , \alpha , \beta )$ for $i=1,2$, such that, for every $\lambda \geqslant \lambda _0$, the following holds. There exists a Borel set $\Omega _\lambda \subset \Omega$ of asymptotically full Lebesgue measure as $\lambda \rightarrow + \infty$, that is $\lim _{\lambda \rightarrow + \infty } |\Omega {\setminus } \Omega _\lambda | = 0$, such that, for every $\omega \in \Omega _\lambda$, there exists a quasi-periodic traveling wave $v_\lambda (\,\cdot \,; \omega ) \in H^s_0({\mathbb {T}}^\nu \times {\mathbb {T}}^2)$ that solves the equation (1.9). Moreover

1.12 $$\begin{aligned} C_1 \lambda ^{\alpha -1} \leqslant \inf _{\omega \in \Omega _\lambda } \Vert v_\lambda (\,\cdot ; \omega ) \Vert _s \leqslant \sup _{\omega \in \Omega _\lambda } \Vert v_\lambda (\,\cdot ; \omega ) \Vert _s \leqslant C_2 \lambda ^{\alpha -1+{\mathtt c}}, \end{aligned}$$

for some ${\mathtt c}\in (0,{\mathtt c}_{0})$ arbitrarily small, with ${\mathtt c}_{0}= {\mathtt c}_{0}(\alpha ,\nu )$. Finally, if we assume that $f({\varphi },x)$ that is even in the pair $({\varphi },x)$, then the solution $v_{\lambda }(\omega t,x)$ is linearly stable.

With Theorem 1.3, we prove that the presence of a large external forcing term that is quasi-periodic traveling and with large frequency of oscillations induces solutions to the forced $\beta$-plane equation (1.9) of large amplitude with the same properties for any value of $\lambda \gg 1$ sufficiently large. Let us make some remarks on the result.

1) *The role of the quasi-periodic traveling structure.* When the free dynamics of a system (that is, without an external force) is invariant by translations in space, traveling wave functions, either periodic or quasi-periodic, form a natural class where to search a solution to the evolution problem that preserves this symmetry. In the context of the model considered here, it is actually an important structure that we need to impose in order to avoid potential degeneracies and multiplicity of the eigenvalues, already at the linear level. More specifically, the linear dynamics of the equation (1.9) is governed by the operator $\lambda \, \omega \cdot \partial _{{\varphi }} + \beta \,{\mathtt L}$, whose spectrum when acting on functions in $H^s({\mathbb {T}}^\nu \times {\mathbb {T}}^2)$ is given by

$$\begin{aligned} \textrm{spec} \big ( \lambda \, \omega \cdot \partial _{{\varphi }} + \beta \,{\mathtt L}\big ) = \big \{ \textrm{i}(\lambda \,\omega \cdot \ell +\beta \,{\mathtt L}(j) ) : \, \ell \in {\mathbb {Z}}^\nu , \ j =(j_1,j_2)^\top \in {\mathbb {Z}}^2 \big \}. \end{aligned}$$

Assuming non-resonance conditions as the parameter varies (that shall be imposed later), the degeneracy is due to the fact the $0\in \textrm{spec} \big ( \lambda \, \omega \cdot \partial _{{\varphi }} + \beta \,{\mathtt L}\big )$ has infinite multiplicity, since it corresponds to any $(\ell ,j)=(0,(0,j_2)^\top )$, with $j_2\in {\mathbb {Z}}$, recalling (1.5). When acting on quasi-periodic traveling waves $v({\varphi },x)=\breve{v}({\varphi }-\pi (x))$, where $({\varphi },x)\in {\mathbb {T}}^\nu \times {\mathbb {T}}^2$ and ${\varphi }-\pi (x)\in {\mathbb {T}}^\nu$ (see also Definition 2.3), it is worth noting that the average in the angle ${\varphi }\in {\mathbb {T}}^\nu$ coincides with the average taken over both coordinates $({\varphi },x)$. This follows from the simple computation

$$\begin{aligned} \int _{{\mathbb {T}}^\nu } v({\varphi },x) \,\textrm{d}{{\varphi }}= \int _{{\mathbb {T}}^\nu } \breve{v}({\varphi }-\pi (x)) \,\textrm{d}{{\varphi }}= \int _{{\mathbb {T}}^\nu } \breve{v}({\varphi }) \,\textrm{d}{{\varphi }}= \text {constant w.r.t.}\,\, x. \end{aligned}$$

At the Fourier level, this implies that if $\ell =0$, then necessarily $j=0$ as well. *The previous degeneracy is avoided when the operator* $\lambda \, \omega \cdot \partial _{{\varphi }} + \beta \,{\mathtt L}$ *acts on quasi-periodic traveling waves.*

2) *The role of the large frequency and of the large forcing term.* The size of the forcing term is $\lambda ^{\alpha }$, with $\lambda \gg 1$. We consider the regime $\alpha \in (1,2)$ for the following reasons. At a purely heuristic level, one can expect the size of a (quasi-)periodic solution to be given by:

1.13 $$\begin{aligned} \text {size of the solution} = \frac{\text {size of the force}}{\text {size of the frequency}} = \frac{O(\lambda ^{\alpha })}{O(\lambda )} = O(\lambda ^{\alpha -1}) . \end{aligned}$$

When $\alpha \in (0,1)$, the expected solution will be of small size with respect to $\lambda \gg 1$. If $\alpha =1$, it will not be of small size, but uniformly bounded in $\lambda \gg 1$. The regime of interest is when $\alpha >1$. However, searching for solutions of size $O(\lambda ^{\alpha -1})$ implies that the quadratic nonlinearity $u \cdot \nabla v$ is of size $O(\lambda ^{2\alpha -2})$. To apply perturbative arguments in our scheme, we need the size of the nonlinear terms to be smaller than the size $O(\lambda ^{\alpha })$ of the external forcing term. This condition implies the restriction $\alpha <2$, which we apply. It is worth noting that we do not know if solutions in the regime $\alpha \geqslant 2$ might exist or not: handling such cases would likely require different strategies and techniques. Moreover, since the construction of the solution deals with small divisors and relative non-resonances conditions, the actual estimates for the solutions do not follow exactly the heuristic in (1.13), but we have to slightly worsen the rate for the upper bound, as stated in (1.12).

We also want to emphasize that the threshold $\lambda _0(f,s,\nu ,\beta )\gg 1$ in Theorem (1.3) needs to be strictly larger than $|\beta |>0$. This condition is crucial to obtain solutions of effective large size, satisfying the lower bound in (1.12). The solutions will exist for any fixed value of $\beta \in {\mathbb {R}}\setminus \{0\}$, regardless of its size. However, it is essential to note that these solutions will exhibit large amplitude only if the fast external oscillations $\lambda \,\omega$, with $\lambda \gg 1$, are stronger than the internal speed of rotation $\beta \ne 0$.

3) *Reversible solutions and linear stability.* It is possible to show that the free dynamics, namely equation (1.7) without the external forcing term, is *reversible*, which is a reflection of the well-known property of the Euler-Coriolis equations. If we assume parity conditions on the external forcing, in particular $f({\varphi },x)$ being even in the pair $({\varphi },x)$, then the equation (1.9) is still reversible and we can adapt our scheme to produce solutions $v_{\lambda }$ in Theorem 1.3 that are odd in the pair $({\varphi },x)$. Moreover, we can prove under this extra symmetry that these quasi-periodic traveling waves solutions are *linearly stable*, meaning that the Floquet exponent of the linearized operator at each solution, with the action of the latter restricted to quasi-periodic traveling waves, are purely imaginary. However, the symmetry of the reversible structure is *not necessary* for the construction of the solutions in Theorem (1.3) itself, but rather only for the analysis of their stability.

#### Literature on quasi-periodic waves in fluids

In the last years, there has been a discrete surge of works proving the existence of time quasi-periodic waves for PDEs arising in fluid dynamics. Most of these results in literature are proved by means of KAM for PDEs techniques, to deal with the presence of small divisors issues and consequent losses of regularity. For the two dimensional water waves equations, we mention Berti and Montalto [11], Baldi, Berti, Haus and Montalto [5] for time quasi-periodic standing waves and Berti, Franzoi and Maspero [8, 9], Feola and Giuliani [23] for time quasi-periodic traveling wave solutions. Recently, the existence of time quasi-periodic solutions was proved for the contour dynamics of vortex patches in active scalar equations. We mention Berti, Hassainia and Masmoudi [10] for vortex patches of the Euler equations close to Kirchhoff ellipses, Hmidi and Roulley [37] for the quasi-geostrophic shallow water equations, Hassainia, Hmidi and Masmoudi [34] for generalized surface quasi-geostrophic equations, Roulley [45] for Euler-$\alpha$ flows, Hassainia and Roulley [36] for Euler equations in the unit disk close to Rankine vortices and Hassainia, Hmidi and Roulley [35] for 2D Euler annular vortex patches. Time quasi-periodic solutions were also constructed for the 3D Euler equations with time quasi-periodic external force [7] and for the forced 2D Navier–Stokes equations [27] approaching in the zero viscosity limit time quasi-periodic solutions of the 2D Euler equations for all times.

We also mention that time quasi-periodic solutions for the Euler equations were constructed also by Crouseilles and Faou [17] in 2D, and by Enciso, Peralta-Salas and de Lizaur [22] in 3D and even dimensions: we remark that these latter solutions are engineered so that there are no small divisors issues to deal with, with consequently much easier proofs and a drawback of not having information on the potential stability of the solutions. Their construction has been recently adapted in [28] to prove the existence of time almost-periodic solutions for the Euler equation in 3D and even dimensions.

We conclude this part of the introduction with the following remarks. Our result, in companion with the recent work [16] on bi-periodic traveling wave solution for the non-resistive MHD system, is the first one in which the existence of *large amplitude* quasi-periodic solutions for a PDE with a forcing term of large size is proved. Up to now, large amplitude quasi-periodic solutions were constructed only for small perturbations of defocusing NLS and KdV equations by Berti, Kappeler and Montalto [12, 13], where the integrable structures of these two equations is exploited. We also mention that KAM techniques have been developed to study the dynamics of linear wave and Klein Gordon equations with potential of size *O*(1) and large frequencies $\lambda \,\omega$ with $\lambda \gg 1$, see [24, 26].

### Strategy of the proof

The solution $v_{\lambda }(\lambda \,\omega t,x)$ in Theorem 1.3 that we are going to construct is a quasi-periodic traveling wave, according to Definition 1.2, of the form, for some $\theta >0$ to determine,

$$\begin{aligned} v_{\lambda }({\varphi },x) = \lambda ^{\theta } w_{\lambda }({\varphi },x) , \quad w_{\lambda }({\varphi },x)=v_{\textrm{app},N} ({\varphi },x) + g({\varphi },x), \end{aligned}$$

so that $v_\lambda = O(\lambda ^{\theta })$ and $w_{\lambda } = O(1)$ with respect to $\lambda \rightarrow \infty$. According to this rescaling, the function $w_{\lambda }({\varphi },x)$ is searched as a quasi-periodic traveling solution of the equation

1.14 $$\begin{aligned} \big (\lambda \,\omega \cdot \partial _{{\varphi }}+ \beta \,{\mathtt L}\big )w_{\lambda } +\lambda ^{\theta } u \cdot \nabla w_{\lambda } = \lambda ^{\alpha -\theta } f(\lambda \, \omega t,x) , \quad u = {\mathfrak {B}}[w_{\lambda }]. \end{aligned}$$

The leading order of function $w_{\lambda }({\varphi },x)$ is the first approximation given by the function $v_{\textrm{app},N}$ of size *O*(1). The function $g({\varphi },x)$, on the other hand, will be a correction to $v_{\textrm{app},N}({\varphi },x)$, bifurcating from the latter, of the smaller size *o*(1) with respect to $\lambda \rightarrow \infty$. If we follow the heuristic as in (1.13), we would expect to simply fix $\theta =\alpha -1$. However, due to the issue of small divisors in inverting the operator $\lambda \,\omega \cdot \partial _{{\varphi }} +\beta \,{\mathtt L}$, we have to worsen the size and take $\theta$ slightly larger than $\alpha -1$ to get an effective upper bound on $w_\lambda$ of size *O*(1). We now sketchily illustrate how to construct these two terms and the main ideas of the strategy.

**Construction of the approximate solution** $v_{\textrm{app},N}$. In order to retrieve a perturbative framework when $\lambda \gg 1$, the first step is to search for a function that solves (1.14) up to some error term that is perturbatively small, namely of size *o*(1) with respect to $\lambda \rightarrow \infty$. We do so by searching for a quasi-periodic traveling wave function $v_{\textrm{app},N}({\varphi },x)$ of the form

1.15 $$\begin{aligned} v_{\textrm{app},N}({\varphi },x) = v_0({\varphi },x) + v_{1}({\varphi },x) +...+ v_{N}({\varphi },x), \end{aligned}$$

where $N\in {\mathbb {N}}_{0}$ is the number of iterations needed to produce the desired correction to the approximation. The first term of this finite expansion is given as the solution of the linear equation

$$\begin{aligned} \big (\lambda \,\omega \cdot \partial _{{\varphi }}+ \beta \,{\mathtt L}\big ) v_{0} = \lambda ^{\alpha -\theta } f . \end{aligned}$$

Building upon the preceding discussion below Theorem 1.3, in order to mitigate the otherwise infinite-dimensional degeneracy in the kernel, the linear operator $\lambda \,\omega \cdot \partial _{{\varphi }}+ \beta \,{\mathtt L}$ has to be inverted on quasi-periodic traveling waves with zero average. But, to deal with the presence of small divisors, we need to impose a non-resonance condition with a consequent loss of derivatives in the action of the inverse. This non-resonance condition is given by

$$\begin{aligned} |\lambda \,\omega \cdot \ell + \beta \, {\mathtt L}(j)| \geqslant \lambda \frac{\gamma }{|\ell |^\tau } , \quad \forall \,(\ell ,j) \in {\mathbb {Z}}^{\nu +2}\setminus \{0\} , \quad \pi ^\top (\ell )+j=0. \end{aligned}$$

for some given $\gamma \in (0,1)$ and $\tau >\nu +1$, where $\pi ^\top (\ell )+j=0$ is the Fourier restriction on the modes of quasi-periodic traveling waves. For this reason, we obtain

1.16 $$\begin{aligned} \Vert \big ( \lambda \,\omega \cdot \partial _{{\varphi }}+ \beta \,{\mathtt L}\big )^{-1} \Vert _{{\mathcal {L}}(H^{s+\tau }_0,H^s_0)} \leqslant O(\lambda ^{-1}\gamma ^{-1}). \end{aligned}$$

To impose smallness conditions later on, the small parameter $\gamma \in (0,1)$ coming from the non-resonance conditions will be linked to $\lambda$ by choosing $\gamma =\lambda ^{-{\mathtt c}}$, for some ${\mathtt c}\in (0,1)$ sufficiently small. This implies that the upper bound in (1.16) is actually of size $O(\lambda ^{-1+{\mathtt c}})$. On the other hand, together with the easy upper bound on the denominator $|\lambda \,\omega \cdot \ell + \beta \,{\mathtt L}(j)| \leqslant \lambda |\omega ||\ell |$, under the assumption that $\lambda \geqslant |\beta |$ and recalling (1.5), we will show that $\Vert \big ( \lambda \,\omega \cdot \partial _{{\varphi }}+ \beta \,{\mathtt L}\big )^{-1} \Vert _{{\mathcal {L}}(H^{s}_0,H^{s+1}_0)} \geqslant O(\lambda ^{-1})$. Therefore, we obtain that

1.17 $$\begin{aligned} O(\lambda ^{\alpha -\theta -1}) \leqslant \Vert v_{0}:= \big (\lambda \,\omega \cdot \partial _{{\varphi }}+ \beta \,{\mathtt L}\big )^{-1}[\lambda ^{\alpha -\theta } f] \Vert _{s} \leqslant O(\lambda ^{\alpha -\theta -1+{\mathtt c}}) . \end{aligned}$$

To get the upper bound in (1.17) of size *O*(1), we fix $\theta =\alpha -1+{\mathtt c}$, whereas the lower bound becomes of size $O(\lambda ^{-{\mathtt c}})$. These estimates of the leading order term of the solutions have the same rate, after rescaling, of the estimates of the whole solution in (1.12). For $N=0$, the error that is produced by approximately solving (1.14) is given by the nonlinear term $\lambda ^{\theta } {\mathfrak {B}}[v_0] \cdot \nabla v_0$, which is of size $O(\lambda ^{\theta })=O(\lambda ^{\alpha -(1-{\mathtt c})})$, smaller than the size of the forcing term $\lambda ^{\alpha -\theta } f= \lambda ^{1-{\mathtt c}}f$ for $\alpha \in (1,2)$ and ${\mathtt c}>0$ small enough so that $\alpha <2(1-{\mathtt c})$. The idea is to iterate this argument to construct the next-order approximate solution $v_1$, which solves the linear equation with a forcing term represented by the error from the previous step. That is

$$\begin{aligned} v_1:= - \big ( \lambda \,\omega \cdot \partial _{{\varphi }}+ \beta \,{\mathtt L}\big )^{-1} [\lambda ^{\theta } {\mathfrak {B}}[v_0] \cdot \nabla v_0] = O(\lambda ^{\theta -1+{\mathtt c}}) , \end{aligned}$$

which is small with respect to $\lambda \rightarrow \infty$, so that the approximate solution $v_{\textrm{app},1}=v_0+v_1$ solves (1.14) up to another error term that is even smaller with respect to the one created by $v_0$ alone. Iterating this procedure for *N* steps, we obtain the approximate solution $v_{\textrm{app},N} = v_0 + v_1 + \ldots + v_N$, where each $v_i$, $i=1,\dots , N$, is small as $\lambda \rightarrow \infty$, and $v_{\textrm{app},N}$ in (1.15) satisfies

$$\begin{aligned} \big (\lambda \,\omega \cdot \partial _{{\varphi }}+ \beta \,{\mathtt L}\big )v_{\textrm{app},N}+\lambda ^{\alpha -1} {\mathfrak {B}}[v_{\textrm{app},N}] \cdot \nabla v_{\textrm{app},N} - \lambda f({\varphi },x) = q_{N}({\varphi },x) , \end{aligned}$$

with $q_N$ a quasi-periodic traveling wave such that $\Vert q_N \Vert _{s} \leqslant O(\lambda ^{N(\alpha -2(1-{\mathtt c}))+\alpha -(1-{\mathtt c})})$. Choosing $N > \frac{\alpha -(1-{\mathtt c})}{2(1-{\mathtt c})-\alpha }$ ensures $\Vert q_N\Vert _s \lesssim o(1)$ as $\lambda \rightarrow \infty$. The detailed construction is provided in Proposition 3.3.

**Linearized operator and Nash-Moser iteration.** We are now in place to search for solution for (1.14) by means of a Nash-Moser iteration scheme, that is we look for the zeroes of the nonlinear functional

1.18 $$\begin{aligned} {\mathcal {F}}(w):=\big (\lambda \,\omega \cdot \partial _{{\varphi }}+ \beta \,{\mathtt L}\big ) w +\lambda ^{\theta } {\mathfrak {B}}[w] \cdot \nabla w - \lambda ^{\alpha -\theta } f({\varphi },x) , \end{aligned}$$

with $\theta :=\alpha -1+{\mathtt c}>0$, as fixed before. We use as initial guess the approximate solution $v_{\textrm{app},N}$ constructed before and we want to find a sequence of quasi-periodic traveling wave functions $(w_n = v_{\textrm{app},N} + g_n)_{n\in {\mathbb {N}}_{0}}$, $g_0:=0$, that converge to a zero of the nonlinear operator ${\mathcal {F}}$. Roughly speaking, the sequence is determined via a Newton scheme, with

$$\begin{aligned} w_{n+1} - w_{n} = - \big (\textrm{d}_{w} {\mathcal {F}}(w_n) \big )^{-1}\big [ {\mathcal {F}}(w_n) \big ]. \end{aligned}$$

The main issue, therefore, is to analyze the linearized operator $\textrm{d}_{w} {\mathcal {F}}(w_n)$, determine its invertibility and estimate the inverse operator. Linearizing (1.18) at $w_n$, we focus on

1.19 $$\begin{aligned} {\mathcal {L}}:= \textrm{d}_{w} {\mathcal {F}}(w_n):= \lambda \,\omega \cdot \partial _{{\varphi }} + \beta \,{\mathtt L}+ \lambda ^{\theta } {\mathfrak {B}}[w_n] \cdot \nabla + \lambda ^{\theta } \nabla w_n \cdot {\mathfrak {B}}. \end{aligned}$$

The main issue is that, in the regime $\lambda \gg 1$, the operator ${\mathcal {L}}$ in (1.19) is a perturbation of large size $O(\lambda ^\theta )$, $\theta < 1$ of the diagonal operator $\lambda \,\omega \cdot \partial _{{\varphi }} + \beta \,{\mathtt L}$. The strategy to establish invertibility and related estimates for the operator ${\mathcal {L}}$ in (1.19) involves reducing it to a diagonal operator with respect to the Fourier basis. This reduction procedure will be carried out in three steps.

1. Reduction of the transport.
2. Reduction to *smoothing* and *perturbative* of the large remainder.
3. KAM reducibility scheme.

If we achieve this reduction with a bounded invertible transformation close to the identity, we can deduce tame estimates for the action of the operator $\big (\textrm{d}_{w} {\mathcal {F}}(w_n) \big )^{-1}$. This operator will also experience a loss of derivatives, akin to the operator $\lambda \,\omega \cdot \partial _{{\varphi }} + \beta \,{\mathtt L}$. However, the Nash-Moser scheme compensates for this loss, ensuring fast convergence of the iterations in a low regularity norm while allowing controlled divergence in a high regularity norm. The invertibility of the operator ${\mathcal {L}}$ in (1.19) is discussed in Sect. 6 after its reduction, while the Nash-Moser iteration is proven in Sect. 7 and finally Theorem 1.3 in Sect. 8.

The remaining part of this introduction will briefly describe the steps involved in the reduction scheme of Sect. 5, which constitutes a substantial part of this paper.

*1) Reduction of the transport.* The first step is to reduce the highest order term in (1.19), which is represented by the transport operator

$$\begin{aligned} {\mathcal {T}}:= \lambda \,\omega \cdot \partial _{{\varphi }} + \lambda ^{\theta } {\mathfrak {B}}[w_n] \cdot \nabla = \lambda \big ( \omega \cdot \partial _{{\varphi }} + \lambda ^{\theta -1} {\mathfrak {B}}[w_n] \cdot \nabla \big ). \end{aligned}$$

Since $\theta -1=\alpha -2+{\mathtt c}<0$ for $\alpha < 2$ and ${\mathtt c}>0$ small enough, the rescaled vector field $\lambda ^{\theta -1} {\mathfrak {B}}[w_n]$ is perturbatively small with respect to $\lambda \rightarrow \infty$. Moreover, it has zero average in space and it is divergence free. Thanks to these properties, we will conjugate the operator ${\mathcal {T}}$ to fully reduce it to the operator $\lambda \,\omega \cdot \partial _{{\varphi }}$, as soon as the frequency vector $\omega \in {\mathbb {R}}^\nu \setminus \{0\}$ is Diophantine (see (3.5)). This is the content of Proposition 5.3, which follows the scheme in [7].

The remaining terms in (1.19), namely $\beta \,{\mathtt L}+ \lambda ^{\theta } \nabla w_n\cdot {\mathfrak {B}}$, undergo the same transformation to an operator of the form $\beta \,{\mathtt L}+ {\mathcal {E}}_{1}$, where ${\mathcal {E}}_{1}$ is a remainder operator of size $\lambda ^{\theta }$ belonging to the class ${{\mathcal {O}}}{{\mathcal {P}}}{{\mathcal {M}}}_{s}^{-1}$ of operators that exhibit 1-smoothing according to off-diagonal matrix decay (see Definition (2.5) and subsequent properties for precise definitions). Additionally, since $w_n({\varphi },x)$ is a quasi-periodic traveling wave, the remainder ${\mathcal {E}}_{1}$ is *momentum preserving*, meaning it maps quasi-periodic traveling waves into quasi-periodic traveling waves (refer to Sect. 2.3 for definitions and characterizations). This property is crucial for the next two steps.

*2) Reduction to a small and smoothing remainder.* After the previous transformation, we are left to work with the operator

$$\begin{aligned} {\mathcal {L}}_{1}:= \lambda \,\omega \cdot \partial _{{\varphi }} + \beta \,{\mathtt L}+ {\mathcal {E}}_{1}. \end{aligned}$$

For the KAM reduction, the remainder ${\mathcal {E}}_{1}$ lacks necessary smoothing for derivative compensation and remains large for $\alpha \in (1,2)$. The next step is to conjugate the operator ${\mathcal {L}}_{1}$ to reduce both the size and order of the remainder ${\mathcal {E}}_{1} \in {{\mathcal {O}}}{{\mathcal {P}}}{{\mathcal {M}}}_{s}^{-1}$. This situation represents a novel development in normal form techniques compared to the previous literature.

To address this, we conjugate the operator ${\mathcal {L}}_{1}$ in a series of $M-1$ iterations, culminating in the remainder ${\mathcal {E}}_{M}$ of the last iteration being of size *o*(1) as $\lambda \rightarrow \infty$ and in the class ${{\mathcal {O}}}{{\mathcal {P}}}{{\mathcal {M}}}_{s}^{-M}$. We briefly descrive the first iteration. We conjugate ${\mathcal {L}}_{1}$ with $\Phi _{1}= \textrm{exp} ({\mathcal {X}}_{1})$, with this transformation being invertible for ${\mathcal {X}}_{1}={\mathcal {X}}_{1}({\varphi }) \in {{\mathcal {O}}}{{\mathcal {P}}}{{\mathcal {M}}}_{s}^{-1}$ sufficiently small. By expanding $\Phi _{1}^{-1} {\mathcal {L}}_{1} \Phi _{1}$ via the standard Lie expansion, the only term of order $- 1$ is given by

$$\begin{aligned} \lambda \,\omega \cdot \partial _{{\varphi }} {\mathcal {X}}_{1} ({\varphi })+ {\mathcal {E}}_{1}({\varphi }). \end{aligned}$$

The sublinear nature of the dispersion relation is crucial. Indeed the effect of the operator $\lambda \,\omega \cdot \partial _{{\varphi }}$ is stronger than the dispersive operator $\beta \, {\mathtt L}$. This fact implies that the commutator $[{\mathcal {X}}_{1} ({\varphi }),\, \beta \,{\mathtt L}]$ is of order $- 2$ and hence it contributes to the remainder of the Lie expansion. We solve the homological equation

1.20 $$\begin{aligned} \lambda \,\omega \cdot \partial _{{\varphi }} {\mathcal {X}}_{1} ({\varphi })+ {\mathcal {E}}_{1}({\varphi }) = {\widehat{{\mathcal {E}}}}_{1}(0) , \quad {\mathcal {E}}_{1}({\varphi }) = \sum _{\ell \in {\mathbb {Z}}^\nu } {\widehat{{\mathcal {E}}}}_{1}(\ell ) e^{\textrm{i}\ell \cdot {\varphi }} \end{aligned}$$

in which we remove the dependence on time from the remainder ${\mathcal {E}}_{1}({\varphi })$ by inverting the operator $\lambda \,\omega \cdot \partial _{{\varphi }}$, provided the frequency vector is $\omega$ is Diophantine. A second key property here is that, since ${\mathcal {E}}_{1}$ is a momentum-preserving operator, the operator ${\widehat{{\mathcal {E}}}}_{1}(0)$, that is the average in time of ${\mathcal {E}}_{1}({\varphi })$, is diagonal with respect to the Fourier basis $(e^{\textrm{i}j \cdot x})_{j\in {\mathbb {Z}}^2}$ (as shown in Lemma 2.13). We obtain that ${\mathcal {X}}_{1} \in {{\mathcal {O}}}{{\mathcal {P}}}{{\mathcal {M}}}_{s}^{-1}$ is of size $O(\lambda ^{\theta -1+{\mathtt c}}) = O(1)$, as $\lambda \rightarrow \infty$, ensuring estimates on the maps $\Phi _{1}^{\pm 1}$ uniform with respect to $\lambda \gg 1$ large enough.

The operator ${\widehat{{\mathcal {E}}}}_{1}(0)$ contributes to a new diagonal operator $\lambda \,\omega \cdot \partial _{{\varphi }} + \beta \,{\mathtt L}+ {\widehat{{\mathcal {E}}}}_{1}(0)$. The new remainder, denoted by ${\mathcal {E}}_{2}$, contains terms that are neither solved by the homological equation (1.20) nor part of the normal form. The leading term of this new remainder is given by the commutator $[{\mathcal {X}}_{1},{\mathcal {E}}_{1}]$, which is of size $O(\lambda ^{2\theta -1+{\mathtt c}})$ and, by algebraic properties, in the class ${{\mathcal {O}}}{{\mathcal {P}}}{{\mathcal {M}}}_{s}^{-2}$. Therefore, the ensuing remainder ${\mathcal {E}}_{2}$ is smaller in size for $\alpha -2(1-{\mathtt c})<0$ (we then take $0 < \mathtt c \ll 1$ small enough) and exhibits more smoothing than the previous remainder ${\mathcal {E}}_{1}$. As in the construction of the approximate solution $v_{\textrm{app},N}$, we iterate this procedure to reduce the remainder to a perturbatively small size. The details of this reduction are provided in Proposition 5.6.

*3) KAM perturbative reduction.* The conclusion of the previous step is the linear operator

$$\begin{aligned} {\mathcal {L}}_{M} = \lambda \,\omega \cdot \partial _{{\varphi }} + \textbf{D}_0 + \textbf{E}_0, \quad \textbf{D}_0:= \beta \,{\mathtt L}+ {\mathcal {Z}}_{M}, \quad \textbf{E}_0:= {\mathcal {E}}_{M}. \end{aligned}$$

Here, the momentum preserving and constant coefficients operator $\lambda \,\omega \cdot \partial _{{\varphi }} + \beta \,{\mathtt L}+ {\mathcal {Z}}_{M}$ is diagonal with respect to the Fourier basis, featuring eigenvalues $\textrm{i}\,\lambda \, \omega \cdot \ell + \mu _{0}(j)$, where $\mu _{0}(j):=\textrm{i}\,\beta \, {\mathtt L}(j) + {\mathtt z}_{0,M}(j)\in {\mathbb {C}}$. For any $\ell \in {\mathbb {Z}}^\nu \setminus \{0\}$ and $j\in {\mathbb {Z}}^2\setminus \{0\}$, the operator ${\mathcal {E}}_{M}\in {{\mathcal {O}}}{{\mathcal {P}}}{{\mathcal {M}}}_{s}^{-M}$ is small, with a size of $O(\lambda ^{M(\theta -1+{\mathtt c})+1-{\mathtt c}})$ as $\lambda \rightarrow \infty$ (note that $\theta -1+{\mathtt c}< 0$). If we assume parity properties on the external forcing term and we preserve the reversible structure of the system in all the steps of the reduction, than we get the diagonal elements ${\mathtt z}_{0,M}(j)$ in ${\mathcal {Z}}_{M}$ are purely imaginary. Now, the goal is to fully reduce the operator ${\mathcal {L}}_{M}$ to a constant-coefficients diagonal operator through a series of conjugations. This process progressively annihilates the size of the perturbative remainder, ultimately yielding the final normal form $\lambda \,\omega \cdot \partial _{{\varphi }} + \beta \,{\mathtt L}+ \textbf{Z}_{\infty }$. A crucial problem, typical in PDEs in higher space dimension, is the presence of strong resonance phenomena. In this case, the difference of unperturbed eigenvalues ${\mathtt L}(j) - {\mathtt L}(j')$ is zero for infinitely many $j, j' \in {\mathbb {Z}}^2 \setminus \{ 0 \}$. Also at this step, this degeneracy issue is overcome by using the conservation of momentum. Indeed, at the first iterative step, the non-resonance conditions, required in the iterative KAM procedure, namey the second-order Melnikov conditions, take the form

1.21 $$\begin{aligned} |\textrm{i}\lambda \,\omega \cdot \ell + \mu _{0}(j)-\mu _{0}(j') | \geqslant \lambda \frac{\gamma }{{\langle {\ell }\rangle }^\tau |j'|^\tau } , \end{aligned}$$

for any $\ell \in {\mathbb {Z}}^\nu \setminus \{0\}$ and $j,j'\in {\mathbb {Z}}^2\setminus \{0\}$. It is noteworthy that the non-resonance conditions in (1.21), needed for estimating small divisors in the solution of the homological equations, lose regularity in both time and space. However, the key feature is the gain in size by a factor $\lambda \gg 1$. This is crucial to impose the appropriate smallness condition, ensuring the convergence of the scheme.

By imposing the non-resonance condition (1.21) above, one can construct a map $\Psi _0$ solving the *homological equation*

$$\begin{aligned} \lambda \, \omega \cdot \partial _{\varphi }\Psi _0({\varphi }) + [\textbf{D}_0,\, \Psi _0({\varphi })] + \textbf{E}_0({\varphi }) = \widehat{\textbf{E}}_0(0), \quad \widehat{\textbf{E}}_0(0) = \int _{{\mathbb {T}}^\nu } \textbf{E}_0({\varphi })\, \,\textrm{d}{{\varphi }}, \end{aligned}$$

and one gets

$$\begin{aligned} e^{- \Psi _1} {{\mathcal {L}}}_M e^{\Psi _1} = \lambda \, \omega \cdot \partial _{\varphi }+ \textbf{D}_0 + \widehat{\textbf{E}}_0(0) + \textbf{E}_1, \end{aligned}$$

where $\textbf{E}_1$ is of order $- M$ and it has size $\textbf{E}_1 \simeq \textbf{E}_0^2$ which is essentially quadratic with respect to the size of $\textbf{E}_0$. It is now crucial to use the conservation of momentum, which allows to deduce that $\widehat{\textbf{E}}_0(0)$ and also $\textbf{D}_1: = \textbf{D}_0 + \widehat{\textbf{E}}_0(0)$ are diagonal operators with respect to the Fourier basis $\{ e^{\textrm{i}j \cdot x}: j \in {\mathbb {Z}}^2 {\setminus } \{ 0 \} \}$.

The detailed construction of the KAM reduction are provided in Sects. 5.3 and 5.4. As remarked below Theorem 1.3, if we assume the preservation of the reversible structure, then we can infer that the final constant-coefficients diagonal normal form has purely imaginary eigenvalues and deduce the linear stability of the final solution. Nevertheless,

the construction of these large amplitude solutions in Theorem 1.3 does not depend on this property.

## Function Spaces, Norms and Linear Operators

### Function spaces

Let $d=2,3$ be the space dimension. Let $a: {\mathbb {T}}^\nu \times {\mathbb {T}}^d \rightarrow E$, $a = a({\varphi },x)$, be a function, with $E = {\mathbb {C}}^m$ or ${\mathbb {R}}^m$. Then, for $s \in {\mathbb {R}}$, its Sobolev norm $\Vert a \Vert _s$ is defined as

$$\begin{aligned} \Vert a \Vert _s^2:= \sum _{(\ell , j) \in {\mathbb {Z}}^\nu \times {\mathbb {Z}}^d} \langle \ell , j \rangle ^{2s} | {\widehat{a}}(\ell ,j) |^2 , \quad \ \langle \ell , j \rangle := \max \{ 1, |\ell |, |j| \} , \end{aligned}$$

where ${\widehat{a}}(\ell ,j)$ (either scalars or vectors) are the Fourier coefficients of $a({\varphi },x)$, namely

$$\begin{aligned} {\widehat{a}}(\ell ,j):= \frac{1}{(2\pi )^{\nu +d}} \int _{{\mathbb {T}}^{\nu +d}} a({\varphi },x) e^{- \textrm{i}(\ell \cdot {\varphi }+ j \cdot x)} \, \,\textrm{d}{{\varphi }}\,\textrm{d}{x} . \end{aligned}$$

We denote, for $E = {\mathbb {C}}^m$ or ${\mathbb {R}}^m$,

$$\begin{aligned} \begin{aligned} H^s&:= H^s_{{\varphi },x}:= H^s({\mathbb {T}}^{\nu } \times {\mathbb {T}}^d) \\&:= H^s({\mathbb {T}}^{\nu } \times {\mathbb {T}}^d, E):= \{ u: {\mathbb {T}}^{\nu } \times {\mathbb {T}}^d \rightarrow E, \ \Vert u \Vert _s < \infty \} . \end{aligned} \end{aligned}$$

In this paper, we use Sobolev norms for (real or complex, scalar- or vector-valued) functions $u( {\varphi }, x; \omega )$, $({\varphi },x) \in {\mathbb {T}}^\nu \times {\mathbb {T}}^d$, being Lipschitz continuous with respect to the parameter $\omega \in {\mathbb {R}}^\nu \setminus \{0\}$. We fix the threshold regularity

2.1 $$\begin{aligned} s_0 \geqslant \nu +d + 3 \end{aligned}$$

and we define the weighted Sobolev norms in the following way.

#### Definition 2.1

*(Weighted Sobolev norms).* Let $\gamma \in (0,1]$, $\Lambda \subseteq {\mathbb {R}}^{\nu }$ be an arbitrary closed set

and $s \geqslant s_0$ with $s_0$ as in (2.1). Given a function $u: \Lambda \rightarrow H^s({\mathbb {T}}^\nu \times {\mathbb {T}}^d)$, $\omega \mapsto u(\omega ) = u({\varphi },x; \omega )$ that is Lipschitz continuous with respect to $\omega$, we define its weighted Sobolev norm by

$$\begin{aligned} \Vert u \Vert _{s}^{\textrm{Lip}(\gamma )}:= \Vert u \Vert _{s,\Lambda }^{\textrm{Lip}(\gamma )}:= \Vert u\Vert _{s}^{\sup } + \gamma \,\Vert u\Vert _{s-1}^\textrm{lip}, \end{aligned}$$

where

$$\begin{aligned} \Vert u\Vert _{s}^{\sup }: =\Vert u\Vert _{s,\Lambda }^{\sup }:= \sup _{\omega \in \Lambda } \Vert u(\omega )\Vert _{s}, \quad \Vert u\Vert _{s}^\textrm{lip}:= \Vert u\Vert _{s,\Lambda }^\textrm{lip}:= \sup _{\omega _1,\omega _2\in \Lambda \atop \omega _1\ne \omega _2} \frac{\Vert u(\omega _1)-u(\omega _2)\Vert _{s}}{| \omega _1-\omega _2|}. \end{aligned}$$

For *u* independent of $({\varphi },x)$, we simply denote by $| u |^{\textrm{Lip}(\gamma )}:= | u|^{\sup } + \gamma \, | u|^\textrm{lip}$.

#### Lemma 2.2

(**Product**). For all $s \geqslant s_0$,

$$\begin{aligned} \Vert uv \Vert _{s}^{\textrm{Lip}(\gamma )}&\lesssim _s C(s) \Vert u \Vert _{s}^{\textrm{Lip}(\gamma )} \Vert v \Vert _{s_0}^{\textrm{Lip}(\gamma )}+ C(s_0) \Vert u \Vert _{s_0}^{\textrm{Lip}(\gamma )} \Vert v \Vert _{s}^{\textrm{Lip}(\gamma )}\,. \end{aligned}$$

**Notation.** We will have also a dependence on ${\mathbb {T}}^{\nu }\ni \varphi \rightarrow w(\varphi , \cdot )\in L^2_0({\mathbb {T}}^2)$ of which we want to control the regularity for the purposes of the nonlinear scheme. In particular, we will ask to be Lipschitz with respect to the function *w* as a parameter and therefore it is natural to introduce the following quantity. Given the map $w \mapsto g(w)$ where *g* is an operator (or a map or a scalar function), we define

2.2 $$\begin{aligned} \Delta _{12} g:= g(w_{2}) - g(w_{1}). \end{aligned}$$

#### Quasi-periodic traveling functions

We restate the definition of quasi-periodic traveling as given in Definition 1.2, for functions of $({\varphi },x)$ instead of (*t*, *x*).

##### Definition 2.3

*(Quasi-periodic traveling waves).* Let ${\overline{\jmath }}_{1},...,{\overline{\jmath }}_{\nu }\in {\mathbb {Z}}^d$ be a given choice of $\nu$ vectors in ${\mathbb {Z}}^d$. A function $u:{\mathbb {T}}^\nu \times {\mathbb {T}}^d\rightarrow {\mathbb {R}}$ is a *quasi-periodic traveling wave* if there exists a function $\breve{u}:{\mathbb {T}}^\nu \rightarrow {\mathbb {R}}$ such that

$$\begin{aligned} u({\varphi },x) = \breve{u}({\varphi }- \pi ( x)) , \quad \forall ({\varphi },x) \in {\mathbb {T}}^\nu \times {\mathbb {T}}^d, \end{aligned}$$

where $\pi :{\mathbb {R}}^d \rightarrow {\mathbb {R}}^\nu$ is the linear map $x \mapsto \pi ( x):= (\overline{\jmath }_{1}\cdot x,...,\overline{\jmath }_{\nu }\cdot x)$.

Comparing with Definition 1.2 (with $d=2$), it is convenient to call *quasi-periodic* traveling wave both the function $u({\varphi },x) = \breve{u}({\varphi }- \pi ( x))$ and the function of time $u(\omega t,x) = \breve{u}(\omega t- \pi ( x))$. We define the translation operator

2.3 $$\begin{aligned} \tau _\varsigma : h(x) \mapsto h(x + \varsigma ),\;\;\varsigma \in {\mathbb {R}}^{d}. \end{aligned}$$

Then, quasi-periodic traveling waves are also characterized by the relation

2.4 $$\begin{aligned} v(\varphi -\pi (\varsigma ),x)= ( \tau _\varsigma \circ v)(\varphi ,x)=v(\varphi ,x+\varsigma ), \quad \forall \,\varphi \in {\mathbb {T}}^\nu ,\; \varsigma \in {\mathbb {R}}^{d}, \; x\in {\mathbb {T}}^{d}. \end{aligned}$$

Expanding in Fourier the equivalence in (2.4), we obtain that a quasi-periodic traveling wave has the form

2.5 $$\begin{aligned} u({\varphi },x) = \sum _{{\begin{array}{c} (\ell , j) \in {\mathbb {Z}}^\nu \times {\mathbb {Z}}^d \\ \pi ^\top (\ell ) + j = 0 \end{array}}} {{\widehat{u}}}(\ell ,j) e^{\textrm{i}(\ell \cdot {\varphi }+ j\cdot x)}. \end{aligned}$$

We define the subspace, for $E= {\mathbb {C}}^m$ or ${\mathbb {R}}^m$,

$$\begin{aligned} S_{\pi }:=\big \{v(\varphi , x)\in L^2({\mathbb {T}}^{\nu +d},E) \,: \, v(\varphi , x)=V(\varphi - \pi ( x)), \ V(\theta )\in L^2({\mathbb {T}}^\nu , E) \big \}. \end{aligned}$$

With abuse of notation, we denote by $S_{\pi }$ also the subspace of $L^2({\mathbb {T}}^{\nu +d}, {\mathbb {R}})$ of scalar functions $v(\varphi , x)$ of the form $v(\varphi , x)=v(\varphi - \pi ( x)),\, V(\theta )\in L^2({\mathbb {T}}^\nu , {\mathbb {R}})$). We note that

$$\begin{aligned} (2.1.1) \quad \Leftrightarrow \quad v\in S_{\pi } \quad \Leftrightarrow \quad \pi ^\top \partial _{\varphi }v+\nabla v=0,\;\;\forall (\varphi ,x)\in {\mathbb {T}}^{\nu +d}. \end{aligned}$$

For any ${\mathtt K}\geqslant 1$, we define the smoothing operators on quasi-periodic traveling waves of the form (2.5) as

2.6 $$\begin{aligned} \begin{aligned} (\Pi _{{\mathtt K}} u)({\varphi },x):= \sum _{{\begin{array}{c} {\langle {\ell }\rangle }\leqslant {\mathtt K},\, j \in {\mathbb {Z}}^d \\ \pi ^\top (\ell ) + j = 0 \end{array}}} {{\widehat{u}}}(\ell ,j) e^{\textrm{i}(\ell \cdot {\varphi }+ j \cdot x)} , \quad \ \Pi ^\perp _{{\mathtt K}}:= \textrm{Id} - \Pi _{{\mathtt K}} . \end{aligned} \end{aligned}$$

Note that, if $u({\varphi },x)$ is a quasi-periodic traveling wave, then both $(\Pi _{{\mathtt K}} u)({\varphi },x)$ and $(\Pi _{{\mathtt K}}^\perp u)({\varphi },x)$ are quasi-periodic traveling waves as well.

##### Lemma 2.4

(**Smoothing**). The smoothing operators $\Pi _{{\mathtt K}}, \Pi _{{\mathtt K}}^\perp$ satisfy the smoothing estimates

$$\begin{aligned} \Vert \Pi _{{\mathtt K}} u \Vert _{s}^{\textrm{Lip}(\gamma )}&\leqslant {\mathtt K}^a \Vert u \Vert _{s-a}^{\textrm{Lip}(\gamma )}\, , \quad 0 \leqslant a \leqslant s \,, \\ \Vert \Pi _{{\mathtt K}}^\bot u \Vert _{s}^{\textrm{Lip}(\gamma )}&\leqslant {\mathtt K}^{-a} \Vert u \Vert _{s + a}^{\textrm{Lip}(\gamma )}\, , \quad a \geqslant 0 \,. \end{aligned}$$

### Matrix representation of linear operators

Let ${{\mathcal {R}}}: L^2({\mathbb {T}}^d) \rightarrow L^2({\mathbb {T}}^d)$ be a linear operator. It can be represented as

2.7 $$\begin{aligned} {{\mathcal {R}}} u (x):= \sum _{j, j' \in {\mathbb {Z}}^d} {\mathcal R}_j^{j'}{\widehat{u}}(j') e^{\textrm{i}j \cdot x} , \quad \text {for} \quad u (x) = \sum _{j \in {\mathbb {Z}}^d} {\widehat{u}}(j) e^{\textrm{i}j \cdot x} , \end{aligned}$$

where, for $j, j' \in {\mathbb {Z}}^d$, the matrix element ${\mathcal R}_j^{j'}$ is defined by

2.8 $$\begin{aligned} {{\mathcal {R}}}_j^{j'}:= \frac{1}{(2\pi )^d} \int _{{\mathbb {T}}^d} {\mathcal R}[e^{\textrm{i}j' \cdot x}] e^{- \textrm{i}j \cdot x} \,\textrm{d}{x}. \end{aligned}$$

We also consider smooth $\varphi$-dependent families of linear operators ${\mathbb {T}}^\nu \rightarrow {{\mathcal {B}}} (L^2({\mathbb {T}}^d))$, $\varphi \mapsto {{\mathcal {R}}}(\varphi )$, which we write in Fourier series with respect to $\varphi$ as

2.9 $$\begin{aligned} {{\mathcal {R}}}(\varphi ) = \sum _{\ell \in {\mathbb {Z}}^\nu } \widehat{\mathcal R}(\ell ) e^{\textrm{i}\ell \cdot \varphi }, \quad \widehat{{\mathcal {R}}}(\ell ):= \frac{1}{(2 \pi )^\nu } \int _{{\mathbb {T}}^\nu } {{\mathcal {R}}}(\varphi ) e^{- \textrm{i}\ell \cdot \varphi }\, \,\textrm{d}{\varphi }, \quad \ell \in {\mathbb {Z}}^\nu . \end{aligned}$$

According to (2.8), for any $\ell \in {\mathbb {Z}}^d$, the linear operator $\widehat{{\mathcal {R}}}(\ell ) \in {\mathcal B} (L^2({\mathbb {T}}^d))$ is identified with the matrix $(\widehat{\mathcal R}(\ell )_j^{j'})_{j, j' \in {\mathbb {Z}}^d}$. A map ${\mathbb {T}}^\nu \rightarrow {{\mathcal {B}}} (L^2({\mathbb {T}}^d))$, $\varphi \mapsto {{\mathcal {R}}}(\varphi )$ can be also regarded as a linear operator $L^2({\mathbb {T}}^{\nu +d}) \rightarrow L^2({\mathbb {T}}^{\nu +d })$ by

2.10 $$\begin{aligned} {{\mathcal {R}}} u(\varphi , x):= \sum _{{\begin{array}{c} \ell , \ell ' \in {\mathbb {Z}}^\nu \\ j, j' \in {\mathbb {Z}}^d \end{array}}} \widehat{{\mathcal {R}}}(\ell - \ell ')_j^{j '} {\widehat{u}}(\ell ', j') e^{\textrm{i}(\ell \cdot \varphi + j \cdot x)}, \quad \forall u \in L^2({\mathbb {T}}^{\nu + d}). \end{aligned}$$

If the operator ${{\mathcal {R}}}$ is invariant on the space of functions with zero average in *x*, we identify ${{\mathcal {R}}}$ with the matrix

$$\begin{aligned} \Big ( \widehat{{\mathcal {R}}}(\ell )_j^{j'} \Big )_{ j, j' \in {\mathbb {Z}}^d \setminus \{ 0 \}, \, \ell \in {\mathbb {Z}}^\nu } . \end{aligned}$$

Let ${{\mathcal {R}}}$ be a linear operator as in (2.7)-(2.10). We define ${\mathcal D}_{{\mathcal {R}}}$ as the operator

2.11 $$\begin{aligned} {{\mathcal {D}}}_{{\mathcal {R}}}:= \textrm{diag}_{j \in {\mathbb {Z}}^2} \widehat{{\mathcal {R}}}(0)_j^j, \quad ({\mathcal {D}}_{{\mathcal {R}}})(\ell )_j^{j'}:= {\left\{ \begin{array}{ll} \widehat{{\mathcal {R}}}(\ell )_j^{j'} &  \quad \text {if} \quad j=j', \ \ell =0, \\ 0 &  \text {otherwise}. \end{array}\right. }. \end{aligned}$$

In particular, we say that ${\mathcal {R}}$ is a *diagonal operator* if ${\mathcal {R}}\equiv {\mathcal {D}}_{{\mathcal {R}}}$.

For the purpose of the Normal Form methods for the linearized operator in Sect. 5, it is convenient to introduce the following norms. These norms take into account both the order and the off-diagonal decay of the matrix elements representing any linear operator on $L^2({\mathbb {T}}^{\nu +d})$.

#### Definition 2.5

*(Matrix decay norm and the class* ${{\mathcal {O}}}{{\mathcal {P}}}{{\mathcal {M}}}^m_s$). Let $m \in {\mathbb {R}}$, $s \geqslant s_0$ and ${\mathcal {R}}$ be an operator represented by the matrix in (2.10). We say that ${{\mathcal {R}}}$ belongs to the class ${{\mathcal {O}}}{{\mathcal {P}}}{{\mathcal {M}}}^m_s$ if

2.12 $$\begin{aligned} | {\mathcal {R}}|_{m, s}:= \sup _{j' \in {\mathbb {Z}}^d} \Big ( \sum _{(\ell ,j) \in {\mathbb {Z}}^{\nu +d}} \langle \ell , j-j' \rangle ^{2s} | \widehat{\mathcal {R}}(\ell )_j^{j'}|^2 \Big )^{\frac{1}{2}} \langle j' \rangle ^{- m} < \infty . \end{aligned}$$

If the operator ${\mathcal {R}}= {\mathcal {R}}(\omega )$ is Lipschitz with respect to the parameter $\omega \in \Lambda \subseteq {\mathbb {R}}^{\nu }$, we define

$$\begin{aligned} \begin{aligned}&| {{\mathcal {R}}} |_{m, s}^{\textrm{Lip}(\gamma )}:= |{{\mathcal {R}}}|_{m, s}^\textrm{sup} + \gamma |{{\mathcal {R}}}|^\textrm{lip}_{m, s - 1}, \\&\quad |{{\mathcal {R}}}|_{m, s}^\textrm{sup}:= \sup _{\omega \in \Lambda } |{{\mathcal {R}}}(\omega )|_{m, s} , \quad |{{\mathcal {R}}}|_{m, s - 1}^\textrm{lip}:= \sup _{{\begin{array}{c} \omega _1, \omega _2 \in \Lambda \\ \omega _1 \ne \omega _2 \end{array}}} \dfrac{|{{\mathcal {R}}}(\omega _1) - {{\mathcal {R}}}(\omega _2)|_{m, s - 1}}{|\omega _1 - \omega _2|} . \end{aligned} \end{aligned}$$

It readily follows that

2.13 $$\begin{aligned} \begin{aligned}&m \leqslant m' \Longrightarrow {{\mathcal {O}}}{{\mathcal {P}}}{{\mathcal {M}}}^m_s \subseteq {{\mathcal {O}}}{{\mathcal {P}}}{{\mathcal {M}}}^{m'}_s \quad \text {and} \quad |\cdot |_{m', s}^{\textrm{Lip}(\gamma )} \leqslant |\cdot |_{m, s}^{\textrm{Lip}(\gamma )}, \\&\quad s \leqslant s' \Longrightarrow {{\mathcal {O}}}{{\mathcal {P}}}{{\mathcal {M}}}^m_{s'} \subseteq {{\mathcal {O}}}{{\mathcal {P}}}{{\mathcal {M}}}^m_s \quad \text {and} \quad | \cdot |_{m, s}^{\textrm{Lip}(\gamma )} \leqslant |\cdot |_{m, s'}^{\textrm{Lip}(\gamma )}. \end{aligned} \end{aligned}$$

We now state some standard properties of the decay norms that are needed for the reducibility scheme of Sect. 5. If $a \in H^s$, $s \geqslant s_0$, then the multiplication operator ${{\mathcal {M}}}_a: u \mapsto a u$ satisfies

$$\begin{aligned} {{\mathcal {M}}}_a \in {{\mathcal {O}}}{{\mathcal {P}}}{{\mathcal {M}}}_s^0 \quad \text {and} \quad |{\mathcal M}_a|_{0, s}^{\textrm{Lip}(\gamma )} \lesssim \Vert a \Vert _s^{\textrm{Lip}(\gamma )}. \end{aligned}$$

#### Lemma 2.6

(*i*) Let $s \geqslant s_0$ and ${{\mathcal {R}}} \in {{\mathcal {O}}}{{\mathcal {P}}}{{\mathcal {M}}}^0_s$. If $\Vert u \Vert _s^{\textrm{Lip}(\gamma )} < \infty$, then

$$\begin{aligned} \Vert {{\mathcal {R}}} u \Vert _s^{\textrm{Lip}(\gamma )} \lesssim _{s} |{\mathcal R}|_{0, s_0}^{\textrm{Lip}(\gamma )} \Vert u \Vert _s^{\textrm{Lip}(\gamma )} + |{{\mathcal {R}}}|_{0, s}^{\textrm{Lip}(\gamma )} \Vert u \Vert _{s_0}^{\textrm{Lip}(\gamma )}; \end{aligned}$$

(*ii*) Let $s \geqslant s_0$, $m, m' \in {\mathbb {R}}$, and let ${{\mathcal {R}}} \in {{\mathcal {O}}}{{\mathcal {P}}}{{\mathcal {M}}}^m_s$, ${{\mathcal {Q}}} \in {{\mathcal {O}}}{{\mathcal {P}}}{{\mathcal {M}}}^{m'}_{s + |m|}$. Then ${{\mathcal {R}}} {{\mathcal {Q}}} \in {{\mathcal {O}}}{{\mathcal {P}}}{{\mathcal {M}}}^{m + m'}_s$ and

$$\begin{aligned} |{{\mathcal {R}}}{{\mathcal {Q}}}|_{m + m', s}^{\textrm{Lip}(\gamma )} \lesssim _{s, m} |{{\mathcal {R}}}|_{m, s}^{\textrm{Lip}(\gamma )} |{\mathcal Q}|_{m', s_0 + |m|}^{\textrm{Lip}(\gamma )} + |{{\mathcal {R}}}|_{m, s_0}^{\textrm{Lip}(\gamma )} |{{\mathcal {Q}}}|_{m', s + |m|}^{\textrm{Lip}(\gamma )}\ ; \end{aligned}$$

(*iii*) Let $s \geqslant s_0$, $m\geqslant 0$ and ${{\mathcal {R}}} \in {{\mathcal {O}}}{{\mathcal {P}}}{{\mathcal {M}}}^{-m}_s$. Then, for any integer $n \geqslant 1$, ${{\mathcal {R}}}^n \in {{\mathcal {O}}}{{\mathcal {P}}}{{\mathcal {M}}}^{-m}_s$ and there exist constants $C(s_0,m), C(s, m) > 0$, independent of *n*, such that

2.14 $$\begin{aligned} \begin{aligned}&|{{\mathcal {R}}}^n|_{-m, s_0}^{\textrm{Lip}(\gamma )} \leqslant C(s_0, m)^{n - 1} \big (|{{\mathcal {R}}}|_{-m, s_0}^{\textrm{Lip}(\gamma )}\big )^{n} , \\&\quad |{{\mathcal {R}}}^n|_{-m, s}^{\textrm{Lip}(\gamma )} \lesssim \, \big (C(s, m)|{{\mathcal {R}}}|_{-m, s_0}^{\textrm{Lip}(\gamma )}\big )^{n - 1} |{{\mathcal {R}}}|_{-m, s}^{\textrm{Lip}(\gamma )}; \end{aligned} \end{aligned}$$

(*iv*) Let $s \geqslant s_0$, $m \geqslant 0$ and ${{\mathcal {R}}} \in {{\mathcal {O}}}{{\mathcal {P}}}{{\mathcal {M}}}^{- m}_s$. Then there exists $\delta (s,m) \in (0, 1)$ small enough such that, if $|{{\mathcal {R}}}|_{-m, s_0}^{\textrm{Lip}(\gamma )} \leqslant \delta (s,m)$, then the map $\Phi := \textrm{exp}({{\mathcal {R}}}) \in {{\mathcal {O}}}{{\mathcal {P}}}{{\mathcal {M}}}^{0}_s$ is invertible and satisfies the estimates

$$\begin{aligned} |\Phi ^{\pm 1}|_{0, s}^{\textrm{Lip}(\gamma )} \lesssim _s 1 + |{\mathcal R}|_{-m, s}^{\textrm{Lip}(\gamma )}; \end{aligned}$$

(*v*) Let $s \geqslant s_0$, $m \in {\mathbb {R}}$ and ${{\mathcal {R}}} \in {{\mathcal {O}}}{{\mathcal {P}}}{{\mathcal {M}}}^m_s$. Let ${{\mathcal {D}}}_{{\mathcal {R}}}$ be the diagonal operator as in (2.11) Then ${{\mathcal {D}}}_{{\mathcal {R}}} \in {{\mathcal {O}}}{{\mathcal {P}}}{{\mathcal {M}}}^m_s$ and $|{{\mathcal {D}}}_{{\mathcal {R}}}|_{m, s}^{\textrm{Lip}(\gamma )} \lesssim |{{\mathcal {R}}}|_{m, s_0}^{\textrm{Lip}(\gamma )}$ for any $s \geqslant s_0$. As a consequence,

$$\begin{aligned} | \widehat{{\mathcal {R}}}(0)_j^j |^{\textrm{Lip}(\gamma )} \lesssim \langle j \rangle ^m|{{\mathcal {R}}}|_{s_0}^{\textrm{Lip}(\gamma )}. \end{aligned}$$

#### Proof

Items (*i*), (*ii*) are proved in Lemma 2.6 in [28]. To prove item (*iii*) we need the following preliminary estimates:

2.15 $$\begin{aligned} \begin{aligned}&|{{\mathcal {R}}}^n|_{0, s_0}^{\textrm{Lip}(\gamma )} \leqslant {\mathtt C}(s_0)^{n - 1} \big (|{{\mathcal {R}}}|_{0, s_0}^{\textrm{Lip}(\gamma )}\big )^{n} , \\&\quad |{{\mathcal {R}}}^n|_{0, s}^{\textrm{Lip}(\gamma )} \leqslant \big ({\mathtt C}(s_0)|{{\mathcal {R}}}|_{0, s_0}^{\textrm{Lip}(\gamma )}\big )^{n - 1} |{{\mathcal {R}}}|_{0, s}^{\textrm{Lip}(\gamma )}. \end{aligned} \end{aligned}$$

One can easily prove them by an induction argument and by using item (*ii*) (see also Lemma 2.6-(*iii*) in [28]). We now prove (2.14). By item (*ii*), (2.15) and (2.13), we compute, for any $n\geqslant 1$,

$$\begin{aligned} \begin{aligned} |{\mathcal {R}}^{n-1} {\mathcal {R}}|_{-m, s_0}^{\textrm{Lip}(\gamma )}&\lesssim _{m,s_0} |{\mathcal {R}}^{n-1}|_{0, s_0}^{\textrm{Lip}(\gamma )} |{\mathcal {R}}|_{-m, s_0}^{\textrm{Lip}(\gamma )} \\&\lesssim _{m,s_0} ({\mathtt C}(s_0))^{n-2}(|{\mathcal {R}}|^{\textrm{Lip}(\gamma )}_{0,s_0})^{n-1}|{\mathcal {R}}|_{-m, s_0}^{\textrm{Lip}(\gamma )} \\&\leqslant c(m,s_0) ({\mathtt C}(s_0))^{n-2}(|{\mathcal {R}}|^{\textrm{Lip}(\gamma )}_{-m,s_0})^{n}, \\ |{\mathcal {R}}^{n-1} {\mathcal {R}}|_{-m, s}^{\textrm{Lip}(\gamma )}&\lesssim _{m,s} |{\mathcal {R}}^{n-1}|_{0, s}^{\textrm{Lip}(\gamma )} |{\mathcal {R}}|_{-m, s_0}^{\textrm{Lip}(\gamma )} + |{\mathcal {R}}^{n-1}|_{0, s_0}^{\textrm{Lip}(\gamma )} |{\mathcal {R}}|_{-m, s}^{\textrm{Lip}(\gamma )} \\&\lesssim _{m,s} ({\mathtt C}(s)|{\mathcal {R}}|^{\textrm{Lip}(\gamma )}_{0,s_0})^{n-2} |{\mathcal {R}}|^{\textrm{Lip}(\gamma )}_{0,s} |{\mathcal {R}}|_{-m, s_0}^{\textrm{Lip}(\gamma )} \\&\quad \, \,+ ({\mathtt C}(s_0))^{n-2}(|{\mathcal {R}}|^{\textrm{Lip}(\gamma )}_{0,s_0})^{n-1}|{\mathcal {R}}|_{-m, s}^{\textrm{Lip}(\gamma )} \\&\leqslant c(m,s) {\mathtt C}(s)^{n-2} (|{\mathcal {R}}|^{\textrm{Lip}(\gamma )}_{-m,s_0})^{n-1}|{\mathcal {R}}|_{-m, s}^{\textrm{Lip}(\gamma )} , \end{aligned} \end{aligned}$$

and the bound follows provided that $C(m,s_0)\geqslant \max \{ c(m,s_0), {\mathtt C}(s_0)\}$ and $C(m,s)\geqslant \max \{ c(m,s), {\mathtt C}(s)\}$. Item (*iv*) follows by recalling that $\Phi ^{\pm 1} = \textrm{exp}(\pm {\mathcal {R}}) = \textrm{Id}+ \sum _{k=1}^{\infty } \frac{{{\mathcal {R}}}^{n}}{n!}$ and by (2.15), (2.13). The claims of item (*v*) are a direct consequence of the definition of the matrix decay norm in Definition 2.12. $\square$

For $N > 0$, we define the operators $\Pi _N {{\mathcal {R}}}$ and $\Pi _N^\perp {\mathcal {R}}$ by means of their matrix representation as follows:

2.16 $$\begin{aligned} (\widehat{\Pi _N {{\mathcal {R}}}})(\ell )_{j}^{j'}:= {\left\{ \begin{array}{ll} \widehat{{\mathcal {R}}}(\ell )_j^{j'} &  \text {if } |\ell |, |j - j'| \leqslant N, \\ 0 &  \text {otherwise}, \end{array}\right. } \qquad \ \Pi _N^\bot {{\mathcal {R}}}:= {{\mathcal {R}}} - \Pi _N {{\mathcal {R}}}.\qquad \end{aligned}$$

#### Lemma 2.7

For all $s, \alpha \geqslant 0$, $m \in {\mathbb {R}}$, one has $|\Pi _N {\mathcal R}|_{m, s + \alpha }^{\textrm{Lip}(\gamma )} \leqslant N^\alpha |{\mathcal R}|_{m, s}^{\textrm{Lip}(\gamma )}$ and $|\Pi _N^\bot {{\mathcal {R}}}|_{m, s}^{\textrm{Lip}(\gamma )} \leqslant N^{- \alpha } |{{\mathcal {R}}}|_{m, s + \alpha }^{\textrm{Lip}(\gamma )}$.

#### Proof

The claims follow directly from (2.12) and (2.16). $\square$

We also define the projection $\Pi _0$ on the space of zero average functions as

2.17 $$\begin{aligned} \Pi _0 h:= \frac{1}{(2 \pi )^{ d}} \int _{{\mathbb {T}}^{ d}} h(\varphi , x)\, \,\textrm{d}{x} , \qquad \Pi _0^\bot := \textrm{Id} - \Pi _0. \end{aligned}$$

In particular, for any $m, s \geqslant 0$,

2.18 $$\begin{aligned} |\Pi _0^\bot |_{0, s} \leqslant 1, \quad |\Pi _0|_{- m, s} \lesssim _{m} 1. \end{aligned}$$

We finally mention the elementary properties of the Laplacian operator $- \Delta$ and its inverse $(- \Delta )^{- 1}$ acting on functions with zero average in *x*:

2.19 $$\begin{aligned} - \Delta u(x) = \sum _{\xi \in {\mathbb {Z}}^d \setminus \{ 0 \}} |\xi |^2 {\widehat{u}}(\xi ) e^{\textrm{i}x \cdot \xi } , \quad (- \Delta )^{- 1} u(x) = \sum _{\xi \in {\mathbb {Z}}^d \setminus \{ 0 \}} \frac{1}{|\xi |^2} \widehat{u}(\xi ) e^{\textrm{i}x \cdot \xi }.\qquad \end{aligned}$$

By Definition 2.5, one easily verifies, for any $s\geqslant 0$,

2.20 $$\begin{aligned} |- \Delta |_{2, s} \leqslant 1, \quad |(- \Delta )^{- 1}|_{- 2, s} \leqslant 1. \end{aligned}$$

Finally, recalling the definition of $\mathtt L$ in (1.5) we have, for any $s\geqslant 0$,

2.21 $$\begin{aligned} | {\mathtt L}|_{-1, s} \leqslant 1. \end{aligned}$$

#### Real and reversible operators

For any function $u({\varphi },x)$, we write $u \in X$ when $u = \textrm{even}({\varphi },x)$, meaning that $u({\varphi },x)=u(-{\varphi },-x)$, and $u \in Y$ when $u = \textrm{odd}({\varphi },x)$, meaning that $u(-{\varphi },-x)=-u({\varphi },x)$.

##### Definition 2.8

(*i*) We say that a linear operator $\Phi$ is *reversible* if $\Phi : X \rightarrow Y$ and $\Phi : Y \rightarrow X$. We say that $\Phi$ is *reversibility preserving* if $\Phi : X \rightarrow X$ and $\Phi : Y \rightarrow Y$.

(*ii*) We say that an operator $\Phi : L^2({\mathbb {T}}^d) \rightarrow L^2({\mathbb {T}}^d)$ is *real* if $\Phi (u)$ is real valued for any *u* real valued.

It is convenient to reformulate real and reversibility properties of linear operators in terms of their matrix representations.

##### Lemma 2.9

A linear operator ${{\mathcal {R}}}$ is:

(*i*) real if and only if $\widehat{\mathcal R}(\ell )_{j}^{j'} = \overline{\widehat{{\mathcal {R}}}(-\ell )_{- j}^{- j'}}$ for all $\ell \in {\mathbb {Z}}^\nu$, $j, j' \in {\mathbb {Z}}^d$;

(*ii*) reversible if and only if $\widehat{\mathcal R}(\ell )_j^{j'} = - \widehat{{\mathcal {R}}}(-\ell )_{- j}^{- j'}$ for all $\ell \in {\mathbb {Z}}^\nu$, $j, j' \in {\mathbb {Z}}^d$;

(*iii*) reversibility-preserving if and only if $\widehat{{\mathcal {R}}}(\ell )_j^{j'} = \widehat{{\mathcal {R}}}(-\ell )_{- j}^{- j'}$ for all $\ell \in {\mathbb {Z}}^\nu$, $j, j' \in {\mathbb {Z}}^d$.

### Momentum preserving operators

The following definition is crucial in the construction of traveling waves.

#### Definition 2.10

*(Momentum preserving)*. A ${\varphi }$-dependent family of linear operators ${\mathcal {R}}({\varphi })$, ${\varphi }\in {\mathbb {T}}^\nu$, is *momentum preserving* if

2.22 $$\begin{aligned} {\mathcal {R}}({\varphi }- \pi (\varsigma ) ) \circ \tau _\varsigma = \tau _\varsigma \circ {\mathcal {R}}({\varphi }) \,, \quad \forall \,{\varphi }\in {\mathbb {T}}^\nu \,, \ \varsigma \in {\mathbb {R}}^d \,, \end{aligned}$$

where the translation operator $\tau _\varsigma$ is defined in (2.3).

In particular, we mention that the operators $-\Delta$ and $(-\Delta )^{-1}$ defined in (2.19) are momentum preserving.

Momentum preserving operators are closed under several operations.

#### Lemma 2.11

Let ${\mathcal {R}}({\varphi }), {\mathcal {Q}}({\varphi })$ be momentum preserving operators. Then:

(i) (Composition): ${\mathcal {R}}({\varphi }) \circ {\mathcal {Q}}({\varphi })$ is a momentum preserving operator;
(ii) (Inversion): If ${\mathcal {R}}({\varphi })$ is invertible then ${\mathcal {R}}({\varphi })^{-1}$ is momentum preserving;
(iii) (Flow): Assume that
  2.23 $$\begin{aligned} \partial _{t} \Phi ^t ({\varphi }) = {\mathcal {R}}({\varphi }) \Phi ^t ({\varphi }) \,, \quad \Phi ^0 ({\varphi }) = \textrm{Id} \,, \end{aligned}$$
  has a unique propagator $\Phi ^t ({\varphi })$ for any $t\in [0,1]$. Then $\Phi ^t ( {\varphi })$ is momentum preserving.

#### Proof

Item (*i*) follows directly by (2.22). Item (*ii*) follows by taking the inverse, of (2.22) and using that $\tau _{-\varsigma } = \tau _\varsigma ^{-1}$. Finally, item (*iii*) holds because $\tau _\varsigma ^{-1} \Phi ^t ( {\varphi }- \pi (\varsigma )) \tau _\varsigma$ solves the same Cauchy problem in (2.23). $\square$

#### Lemma 2.12

Let ${\mathcal {R}}({\varphi })$ be a momentum preserving linear operator and *u* a quasi-periodic traveling wave, according to Definition 2.3. Then ${\mathcal {R}}({\varphi }) u$ is a quasi-periodic traveling wave.

#### Proof

It follows by Definition 2.10 and by the characterization of traveling waves in (2.4). $\square$

We now provide a characterization of the momentum preserving property in Fourier space. In particular, momentum preserving operators that are independent of ${\varphi }\in {\mathbb {T}}^\nu$ are actually diagonal.

#### Lemma 2.13

A ${\varphi }$-dependent family of operators ${\mathcal {R}}({\varphi })$, with ${\varphi }\in {\mathbb {T}}^\nu$, is momentum preserving if and only if the matrix elements of ${\mathcal {R}}({\varphi })$, defined by (2.10), fulfill

2.24 $$\begin{aligned} {\widehat{{\mathcal {R}}}}(\ell )_{j}^{j'} \ne 0 \quad \Rightarrow \quad \pi ^\top ( \ell ) + j-j' = 0 \,, \quad \forall \, \ell \in {\mathbb {Z}}^\nu , \ \ j,j'\in {\mathbb {Z}}^d \,. \end{aligned}$$

As a consequence we have that, for any momentum preserving operator ${\mathcal {R}}({\varphi })$, the operator $\widehat{{\mathcal {R}}}(0)$ satisfies

2.25 $$\begin{aligned} {\widehat{{\mathcal {R}}}}(0)_{j}^{j'} \ne 0 \quad \Rightarrow \quad j=j' , \end{aligned}$$

that is, ${{\widehat{{\mathcal {R}}}}}(0)$ is a time-independent diagonal operator (recall (2.11)).

#### Proof

By (2.10) we have, for any function *u*(*x*),

$$\begin{aligned} \tau _\varsigma ( {\mathcal {R}}({\varphi }) u ) = \sum _{j,j'\in {\mathbb {Z}}^d}\sum _{\ell \in {\mathbb {Z}}^\nu } {\widehat{{\mathcal {R}}}}(\ell )_j^{j'} e^{\textrm{i}j \cdot \varsigma } u_{j'} e^{\textrm{i}(\ell \cdot {\varphi }+ j\cdot x )} \end{aligned}$$

and

$$\begin{aligned} {\mathcal {R}}({\varphi }-\pi (\varsigma )) [\tau _\varsigma u] = \sum _{j,j'\in {\mathbb {Z}}^d}\sum _{\ell \in {\mathbb {Z}}^\nu } {\widehat{{\mathcal {R}}}}(\ell )_j^{j'} e^{- \textrm{i}\ell \cdot \pi (\varsigma )} e^{\textrm{i}j' \cdot \varsigma } u_{j'} e^{\textrm{i}(\ell \cdot {\varphi }+ j\cdot x )} \,. \end{aligned}$$

Therefore, using that $\ell \cdot \pi (\varsigma ) = \pi ^\top (\ell )\cdot \varsigma$, we obtain that (2.22) is equivalent to (2.24). The claim in (2.25) follows immediately by (2.24): indeed, assuming that the matrix elements $\big ( {\widehat{{\mathcal {R}}}}(0)_{j}^{j'}\big )_{j,j'\in {\mathbb {Z}}^d, \, \ell \in {\mathbb {Z}}^\nu }$ of ${{\widehat{{\mathcal {R}}}}} (0)$ are zero outside the diagonal, by using the momentum condition $\pi ^\top ( \ell ) + j-j' = 0$, for $\ell = 0$. $\square$

### Conjugations with change of variables

Here we recall some results from [7] regarding certain properties of linear operators with matrix decay that are conjugated by a change of variables

2.26 $$\begin{aligned} {\mathcal {A}}={\mathcal {A}}({\varphi }): u \mapsto {\mathcal {A}}({\varphi }) u, \quad ({\mathcal {A}}({\varphi })u)(x):= u(x+{\varvec{\alpha }}({\varphi },x)), \end{aligned}$$

where ${\varvec{\alpha }}: {\mathbb {T}}^\nu \times {\mathbb {T}}^d \rightarrow {\mathbb {R}}^d$. We have the following lemmata.

#### Lemma 2.14

Let $\Vert {\varvec{\alpha }}\Vert _{s_0}^{\textrm{Lip}(\gamma )}\leqslant \delta (s_0)$ sufficiently small. The composition operator ${\mathcal {A}}$ in (2.26) satisfies the tame estimates, for any $s\geqslant s_0$,

$$\begin{aligned} \Vert {\mathcal {A}}u \Vert _{s}^{\textrm{Lip}(\gamma )} \lesssim _{s} \Vert u\Vert _{s}^{\textrm{Lip}(\gamma )} + \Vert {\varvec{\alpha }}\Vert _{s}^{\textrm{Lip}(\gamma )} \Vert u \Vert _{s_0}^{\textrm{Lip}(\gamma )} . \end{aligned}$$

Moreover, it is an invertible map, with inverse given by

2.27 $$\begin{aligned} {\mathcal {A}}^{-1}= {\mathcal {A}}({\varphi })^{-1}: u \mapsto {\mathcal {A}}({\varphi })^{-1} u , \quad ({\mathcal {A}}({\varphi })^{-1} u)(y):= u(y+\breve{{\varvec{\alpha }}}({\varphi },y)), \end{aligned}$$

for some function $\breve{{\varvec{\alpha }}}({\varphi },y)$ such that $x:=y+\breve{{\varvec{\alpha }}}({\varphi },y)$ is the inverse diffeomorphism of the map $y:=x+{\varvec{\alpha }}({\varphi },x)$, with estimates, for any $s\geqslant s_0$,

$$\begin{aligned} \begin{aligned}&\Vert \breve{{\varvec{\alpha }}}\Vert _{s}^{\textrm{Lip}(\gamma )}\lesssim _{s} \Vert {\varvec{\alpha }}\Vert _{s}^{\textrm{Lip}(\gamma )} , \quad \Vert {\mathcal {A}}^{-1} u \Vert _{s}^{\textrm{Lip}(\gamma )} \lesssim _{s} \Vert u\Vert _{s}^{\textrm{Lip}(\gamma )} + \Vert {\varvec{\alpha }}\Vert _{s}^{\textrm{Lip}(\gamma )} \Vert u \Vert _{s_0}^{\textrm{Lip}(\gamma )} , \\&\quad \Vert ({\mathcal {A}}^{\pm 1} -\textrm{Id})u \Vert _{s}^{\textrm{Lip}(\gamma )} \lesssim _{s} \Vert {\varvec{\alpha }}\Vert _{s_0+1}^{\textrm{Lip}(\gamma )} \Vert u\Vert _{s+1}^{\textrm{Lip}(\gamma )} + \Vert {\varvec{\alpha }}\Vert _{s+1}^{\textrm{Lip}(\gamma )} \Vert u \Vert _{s_0+1}^{\textrm{Lip}(\gamma )} . \end{aligned} \end{aligned}$$

#### Proof

We refer to Lemma 2.3-(*ii*), (*iii*) in [7] for the proof. $\square$

#### Lemma 2.15

If ${\varvec{\alpha }}({\varphi }, x )$ is a quasi-periodic traveling wave, then the operators ${\mathcal {A}}({\varphi })$ and ${\mathcal {A}}({\varphi })^{-1}$ defined in (2.26)-(2.27) are momentum preserving.

#### Proof

We have

$$\begin{aligned} \begin{aligned} {\mathcal {A}}({\varphi }- \pi (\varsigma )) [\tau _\varsigma u]&= u(x+ {\varvec{\alpha }}({\varphi }- \pi (\varsigma ),x) + \varsigma ) \\&= u(x+ \varsigma + {\varvec{\alpha }}({\varphi },x+ \varsigma )) = \tau _\varsigma \big ( {\mathcal {A}}({\varphi }) u\big ) . \end{aligned} \end{aligned}$$

This proves that ${\mathcal {A}}({\varphi })$ is momentum preserving. By Lemma 2.11-(*ii*), we have that ${\mathcal {A}}({\varphi })^{-1}$ is momentum preserving as well. $\square$

#### Lemma 2.16

(**Conjugation of the Laplacian**). Let $S>s_0$ and ${\varvec{\alpha }}\in H^{S+2}({\mathbb {T}}^{\nu +d})$, with $\Vert {\varvec{\alpha }}\Vert _{s_0+\mu }^{\textrm{Lip}(\gamma )}\leqslant \delta$ for some $\delta =\delta (d,\nu )\in (0,1)$ small enough and for some $\mu =\mu (d,\nu )>0$. The following hold:

(*i*) The operator $-\Delta$ acting on functions with zero average in *x*, as defined in (2.19), is conjugated via (2.26)-(2.27) to the operator ${\mathcal {P}}_{-\Delta }$ acting on functions with zero average in *x* defined as

2.28 $$\begin{aligned} {\mathcal {P}}_{-\Delta }:= \Pi _0^\perp {\mathcal {A}}^{-1} (-\Delta ) {\mathcal {A}}\Pi _0^\perp = -\Delta + {\mathcal {P}}_{2}, \end{aligned}$$

where ${\mathcal {P}}_{2} \in {{\mathcal {O}}}{{\mathcal {P}}}{{\mathcal {M}}}^2_s$ satisfies, for any $s_0\leqslant s\leqslant S$,

2.29 $$\begin{aligned} |{\mathcal {P}}_{2} |_{2,s}^{\textrm{Lip}(\gamma )} \lesssim _{s} \Vert {\varvec{\alpha }}\Vert _{s+\mu }^{\textrm{Lip}(\gamma )}. \end{aligned}$$

Furthermore, if ${\varvec{\alpha }}({\varphi },x)$ is a quasi-periodic traveling wave, then ${\mathcal {P}}_{-\Delta }$ and ${\mathcal {P}}_{2}$ are momentum preserving, according to Definition 2.10;

(*ii*) The operator ${\mathcal {P}}_{-\Delta }$ in (2.28) is invertible on the functions of zero average in *x*, with inverse given by

2.30 $$\begin{aligned} {\mathcal {P}}_{-\Delta }^{-1}:= \Pi _0^\perp {\mathcal {A}}^{-1} (-\Delta )^{-1} {\mathcal {A}}\Pi _0^\perp = (-\Delta )^{-1}+ {\mathcal {P}}_{-2}, \end{aligned}$$

where the operator $(-\Delta )^{-1}$ acting on functions with zero average in *x* is defined in (2.19), whereas ${\mathcal {P}}_{-2} \in {{\mathcal {O}}}{{\mathcal {P}}}{{\mathcal {M}}}^{-2}$ satisfies, for any $s_0\leqslant s\leqslant S$,

2.31 $$\begin{aligned} |{\mathcal {P}}_{-2} |_{-2,s}^{\textrm{Lip}(\gamma )} \lesssim _{s} \Vert {\varvec{\alpha }}\Vert _{s+\mu }^{\textrm{Lip}(\gamma )}. \end{aligned}$$

Furthermore, if ${\varvec{\alpha }}({\varphi },x)$ is a quasi-periodic traveling wave, then ${\mathcal {P}}_{-\Delta }^{-1}$ and ${\mathcal {P}}_{-2}$ are momentum preserving, according to Definition 2.10.

#### Proof

We start with item (*i*). We compute explicitly

$$\begin{aligned} \begin{aligned} {\mathcal {A}}^{-1} (-\Delta ) {\mathcal {A}}= -\Delta + \sum _{i,j=1}^{d} a_{ij}({\varphi },y) \partial _{y_i} \partial _{y_j} + \sum _{j=1}^{d} b_{j} ({\varphi },y) \partial _{y_j} , \end{aligned} \end{aligned}$$

where, for $$i,j=1,...,d$$,

2.32 $$\begin{aligned} \begin{aligned} a_{jj}({\varphi },y)&:= - {\mathcal {A}}^{-1} \big \{ | \nabla (x_j + \alpha _{j}({\varphi },x)) |^2 - 1 \big \}, \quad i= j , \\ a_{i,j}({\varphi },y)&:= - 2 {\mathcal {A}}^{-1}\big \{ \nabla (x_i + \alpha _{i}({\varphi },x)) \cdot \nabla (x_j + \alpha _{i}({\varphi },x)) \big \} , \quad i\ne j ,\\ b_{j}({\varphi },y)&:= - {\mathcal {A}}^{-1} \big \{ \Delta \alpha _{j}({\varphi },x) \big \}, \quad {\varvec{\alpha }}({\varphi },x) = (\alpha _{1}({\varphi },x),...,\alpha _{d}({\varphi },x)). \end{aligned} \end{aligned}$$

We conclude that the operator ${\mathcal {P}}_{2}$ in (2.28) is given by

$$\begin{aligned} {\mathcal {P}}_{2}:= \Pi _0^\perp \Big ( \sum _{i,j=1}^{d} a_{ij}({\varphi },y) \partial _{y_i} \partial _{y_j} + \sum _{j=1}^{d} b_{j} ({\varphi },y) \partial _{y_j} \Big ) , \end{aligned}$$

and the estimate (2.29) follows by the explicit formulae of the coefficients in (2.32), Lemma 2.6-(*ii*) and (2.18). Moreover, if ${\varvec{\alpha }}({\varphi },x)$ is a traveling wave, and since the operators $-\Delta$ and $\Pi _0^\perp$ are momentum preserving, it follows from Lemma  2.15 that the operators ${\mathcal {P}}_{-\Delta }$ and ${\mathcal {P}}_{2}$ are momentum preserving as well.

We now prove item (*ii*). First, note that

$$\begin{aligned} \begin{aligned} {\mathcal {P}}_{-\Delta } {\mathcal {P}}_{-\Delta }^{-1}&= \Pi _0^\perp {\mathcal {A}}^{-1} (-\Delta ) {\mathcal {A}}\Pi _0^\perp {\mathcal {A}}^{-1} (-\Delta )^{-1} {\mathcal {A}}\Pi _0^\perp \\&= \Pi _0^\perp - \Pi _0^\perp {\mathcal {A}}^{-1} (-\Delta ) {\mathcal {A}}\Pi _0 {\mathcal {A}}^{-1} (-\Delta )^{-1} {\mathcal {A}}\Pi _0^\perp \\&= \Pi _0^\perp - \Pi _0^\perp {\mathcal {A}}^{-1} (-\Delta ) \Pi _0 {\mathcal {A}}^{-1} (-\Delta )^{-1} {\mathcal {A}}\Pi _0^\perp = \Pi _0^\perp , \end{aligned} \end{aligned}$$

since ${\mathcal {A}}\Pi _0 = \Pi _0$ and $(-\Delta )\Pi _0 = 0$, so that ${\mathcal {P}}_{-\Delta }^{-1}$ defined in (2.30) is indeed the inverse of ${\mathcal {P}}_{-\Delta }$ on the space of function with zero average with respect to *x*. Next, we write

$$\begin{aligned} {\mathcal {P}}_{-\Delta } = -\Delta + {\mathcal {P}}_{2} = (-\Delta ) \big ( \textrm{Id} + F \big ), \quad F:= (-\Delta )^{-1} {\mathcal {P}}_{2}, \end{aligned}$$

where, by Lemma 2.6-(*ii*) and (2.20), we have $F\in {{\mathcal {O}}}{{\mathcal {P}}}{{\mathcal {M}}}_{s}^0$ and, for any $s_0 \leqslant s \leqslant S$,

$$\begin{aligned} | F|_{0,s}^{\textrm{Lip}(\gamma )} \lesssim _{s} | {\mathcal {P}}_{2}|_{2,s+2}^{\textrm{Lip}(\gamma )} \lesssim _{s} \Vert {\varvec{\alpha }}\Vert _{s+\mu }^{\textrm{Lip}(\gamma )}. \end{aligned}$$

Then, for $\Vert {\varvec{\alpha }}\Vert _{s_0 +\mu }^{\textrm{Lip}(\gamma )}$ sufficiently small, the operator $\textrm{Id}+ F \in {{\mathcal {O}}}{{\mathcal {P}}}{{\mathcal {M}}}_{s}^0$ is invertible by a standard Neumann series argument, with inverse $(\textrm{Id}+ F)^{-1} \in {{\mathcal {O}}}{{\mathcal {P}}}{{\mathcal {M}}}_{s}^0$. We obtain that

$$\begin{aligned} {\mathcal {P}}_{-\Delta }^{-1} = (\textrm{Id} + F)^{-1} (-\Delta )^{-1} = (-\Delta )^{-1} + {\mathcal {P}}_{-2} , \end{aligned}$$

where, by Lemma 2.6 and (2.20),

$$\begin{aligned} {\mathcal {P}}_{-2}:= \big [ (\mathrm Id + F)^{-1} - \textrm{Id} \big ] (-\Delta )^{-1} \in {{\mathcal {O}}}{{\mathcal {P}}}{{\mathcal {M}}}_{s}^{-2}, \end{aligned}$$

and, for any $s_0\leqslant s \leqslant S$, satisfies the estimate (2.31). Moreover, if ${\varvec{\alpha }}({\varphi },x)$ is a traveling wave, then ${\mathcal {P}}_{-\Delta }^{-1}$ and ${\mathcal {P}}_{-2}$ are momentum preserving by item (*i*), Lemma 2.11 and the fact that the operator $(-\Delta )^{-1}$ is momentum preserving. $\square$

## Construction of the Approximate Solution

The first step in the search for a solution of (1.9) is to find an approximate solution of size $O(\lambda ^{\theta })$, with $\alpha - 1< \theta < 1$ to be determined. By rescaling the unknown $v \rightsquigarrow \lambda ^{\theta } v$ in (1.9), we look for quasi-periodic traveling wave solutions $v({\varphi },x)$ of size *O*(1) as zeroes of the nonlinear map ${{\mathcal {F}}}(v) \equiv {{\mathcal {F}}}(v; \omega , \lambda )$ of the form

3.1 $$\begin{aligned} \begin{aligned}&{{\mathcal {F}}}(v):= \lambda \,\omega \cdot \partial _{{\varphi }} v + \beta \,{\mathtt L}v + \lambda ^{\theta } {{\mathcal {N}}}(v,v) - \lambda ^{\alpha -\theta } f({\varphi },x) =0, \\&\quad {{\mathcal {N}}}(v_1,v_2): = {\mathfrak {B}}[v_1] \cdot \nabla v_2. \end{aligned} \end{aligned}$$

If we want to construct *reversible* solutions, we require the forcing $f({\varphi },x)$ to satisfy the symmetry condition

3.2 $$\begin{aligned} f({\varphi },x) = \textrm{even}({\varphi },x), \end{aligned}$$

where $\textrm{even}({\varphi },x)$ denotes a function which is even in both variables ${\varphi }$ and *x*. Given $v(\varphi , x)$ a quasi-periodic traveling wave, we denote by ${{\mathcal {L}}} \equiv {{\mathcal {L}}}(v):= \textrm{d}_{v}{{\mathcal {F}}}(v)$ the linearized operator near *v*:

3.3 $$\begin{aligned} \begin{aligned}&{{\mathcal {L}}} = \lambda \, \omega \cdot \partial _\varphi + \beta \, {\mathtt L}+ \lambda ^{\theta } \big ( \textbf{a}_0(\varphi , x) \cdot \nabla + {{\mathcal {E}}}_0 \big ), \\&\quad \textbf{a}_0(\varphi , x): = {\mathfrak {B}}\big [ v \big ] (\varphi , x) , \quad {\mathcal {E}}_{0}[h]:= \nabla v \cdot {\mathfrak {B}}[h] , \end{aligned} \end{aligned}$$

where ${\mathtt L}$ is defined in (1.5) and the Biot-Savart operator ${\mathfrak {B}}:= \nabla ^\perp (-\Delta )^{-1}$ is as in (1.8).

From now on, the dimension $d=2$ is fixed once and for all, so that all the definitions and properties in Sect. 2 will be applied with $d=2$.

First we establish some quantitative estimates on ${{\mathcal {F}}}$ that we shall use in the sequel and its behaviour when acting on quasi-periodic traveling waves.

### Lemma 3.1

Let $$s_0>0 $$ be as in (2.1). The following hold:

(*i*) Assume that $s > s_0$, $v(\,\cdot \,; \omega ) \in H^{s + 1}_0({\mathbb {T}}^{\nu + 2})$ and $\Vert v \Vert _{s_0 + 1}^{\textrm{Lip}(\gamma )} \lesssim 1$. Then the operator ${\mathcal {F}}(v)$ in (3.1) satisfies the following estimate:

$$\begin{aligned} \Vert {{\mathcal {F}}}(v) \Vert _s^{\textrm{Lip}(\gamma )} \lesssim _s \lambda \big (1 + \Vert v \Vert _{s + 1}^{\textrm{Lip}(\gamma )} \big ). \end{aligned}$$

Moreover, if *v* is a quasi-periodic traveling wave, then ${\mathcal {F}}(v)$ is a quasi-periodic traveling wave. In addition, assuming (3.2), if $v({\varphi },x)=\textrm{odd}({\varphi },x)$, then ${\mathcal {F}}(v)({\varphi },x)=\textrm{even}({\varphi },x)$;

(*ii*) Assume that $\Vert v \Vert _{s_0 + 1}^{\textrm{Lip}(\gamma )} \lesssim 1$. Then, for any $b\geqslant 1$, $h(\,\cdot \,;\omega ) \in H^{s_0 + b}_0({\mathbb {T}}^{\nu + 2})$ and ${\mathtt K}\in {\mathbb {N}}$,

$$\begin{aligned} \begin{aligned}&\Vert {{\mathcal {L}}} h \Vert _{s_0}^{\textrm{Lip}(\gamma )} \lesssim \lambda \Vert h \Vert _{s_0 + 1}^{\textrm{Lip}(\gamma )}, \\&\quad \Vert [{\mathcal {L}},\Pi _{{\mathtt K}}^\perp ] h \Vert _{s_0}^{\textrm{Lip}(\gamma )} \lesssim \lambda ^{\theta } {\mathtt K}^{1-b}\big ( \Vert h \Vert _{s_0+b}^{\textrm{Lip}(\gamma )} + \Vert v \Vert _{s_0+b}^{\textrm{Lip}(\gamma )} \Vert h \Vert _{s_0+1}^{\textrm{Lip}(\gamma )} \big ) , \\&\quad \Vert [\Pi _{{\mathtt K}},{\mathcal {L}}] h \Vert _{s_0+b}^{\textrm{Lip}(\gamma )} \lesssim \lambda ^{\theta } {\mathtt K}\big ( \Vert h \Vert _{s_0+b}^{\textrm{Lip}(\gamma )} + \Vert v \Vert _{s_0+b+1}^{\textrm{Lip}(\gamma )} \Vert h \Vert _{s_0+1}^{\textrm{Lip}(\gamma )} \big ) . \end{aligned} \end{aligned}$$

Moreover, if *h* is a quasi-periodic traveling wave, then ${\mathcal {L}}h$ is a quasi-periodic traveling wave. In addition, if $h({\varphi },x)=\textrm{odd}({\varphi },x)$, then ${\mathcal {L}}h({\varphi },x)=\textrm{even}({\varphi },x)$;

(*iii*) Let $v(\,\cdot \,;\omega ), \,h(\,\cdot \,;\omega ) \in H_{0}^{s + 1}({\mathbb {T}}^{\nu + 2})$. Then

$$\begin{aligned} Q (v, h):= {{\mathcal {F}}}(v + h) - {{\mathcal {F}}}(v) - {{\mathcal {L}}} h = \lambda ^\theta {\mathcal {N}}(h,h) \end{aligned}$$

satisfies

$$\begin{aligned} \int _{{\mathbb {T}}^2} Q(v,h)({\varphi },x) \,\textrm{d}{x} = 0 , \quad \Vert Q(v, h) \Vert _{s}^{\textrm{Lip}(\gamma )} \lesssim \lambda ^{\theta } \Vert h \Vert _{s_0 + 1}^{\textrm{Lip}(\gamma )} \Vert h \Vert _{s + 1}^{\textrm{Lip}(\gamma )}\ . \end{aligned}$$

Moreover, if *v* and *h* are quasi-periodic traveling waves, then *Q*(*v*, *h*) is a quasi-periodic traveling wave. In addition, assuming (3.2), if $v({\varphi },x),h({\varphi },x)=\textrm{odd}({\varphi },x)$, then $Q(v,h)({\varphi },x)=\textrm{even}({\varphi },x)$.

### Proof

The claimed estimates follow by (3.3), using that $\alpha -\theta <1$, $Q(v, h) = \lambda ^{\theta }{{\mathcal {N}}}(h, h)$, by a repeated applications of the interpolation estimate (2.2), and by the fact that $\Pi _{{\mathtt K}}$ and $\Pi _{{\mathtt K}}^\perp$ commute with $\lambda \,\omega \cdot \partial _{{\varphi }} + \beta \,{\mathtt L}$. Moreover, by (3.1) and using that $\textrm{div} {\mathfrak {B}}[h]= 0$ for any sufficiently smooth function $h({\varphi }, x)$, by (1.8), we have

$$\begin{aligned} \int _{{\mathbb {T}}^2} Q(v,h)({\varphi },x) \,\textrm{d}{x}  &   = \lambda ^{\theta } \int _{{\mathbb {T}}^2} {\mathfrak {B}}[h({\varphi },x)] \cdot \nabla h({\varphi },x) \,\textrm{d}{x} \\  &   = - \lambda ^\theta \int _{{\mathbb {T}}^2} \textrm{div} {\mathfrak {B}}[h({\varphi },x)] h({\varphi },x) \,\textrm{d}{x} = 0 . \end{aligned}$$

The claims on the quasi-periodic traveling waves follow by (3.1), (3.3), the fact that $f({\varphi },x)$ is a quasi-periodic function, and Lemmata 2.11, 2.12. Finally, assuming (3.2), the claims on the parities follow by (3.1), (1.5), (1.8), (3.3) and Lemma 2.9. $\square$

For $N\in {\mathbb {N}}_0$, we construct an approximate solution of (3.1) of the form

3.4 $$\begin{aligned} \begin{aligned} v_{\textrm{app},N}({\varphi },x)&= v_0({\varphi },x)+v_1({\varphi },x)+...+v_N({\varphi },x) \\&= v_{\textrm{app},N-1}({\varphi },x) + v_{N}({\varphi },x), \quad v_{\textrm{app},-1}:= 0 , \end{aligned} \end{aligned}$$

in such a way that the function $v_{\textrm{app}, N}({\varphi },x)$ of size *O*(1) solves (3.1) up to a progressively smaller error term which, for $N\in {\mathbb {N}}$ sufficiently large, becomes perturbatively small for $\lambda \gg 1$ big enough, see (3.14) and (3.15) in Proposition 3.3. Moreover, both the approximate solution $v_{\textrm{app},N}({\varphi },x)$ that we are going to construct and the final error term are quasi-periodic traveling waves.

For fixed $\gamma \in (0,1)$ and $\tau > \nu -1$, recalling (1.11), we define the set of the Diophantine frequency vectors

3.5 $$\begin{aligned} {\mathtt D}{\mathtt C}(\gamma ,\tau ):= \Big \{ \omega \in \Omega : \ |\omega \cdot \ell | \geqslant \gamma {\langle {\ell }\rangle }^{-\tau } \ \forall \,\ell \in {\mathbb {Z}}^{\nu }\setminus \{0\} \Big \} . \end{aligned}$$

It is well known that the Lebesgue measure $|\Omega {\setminus } {\mathtt D}{\mathtt C}(\gamma ,\tau ) | = O(\gamma )$. Moreover, we consider the following non-resonance condition, for $\tau > \nu + 1$,

3.6 $$\begin{aligned} \begin{aligned} \Omega _{\gamma }:= \Big \{ \omega&\in {\mathtt D}{\mathtt C}(\gamma ,\tau ) : | \lambda \,\omega \cdot \ell + \beta \,{\mathtt L}(j) | \geqslant \lambda \tfrac{\gamma }{ {\langle {\ell }\rangle }^{\tau }}, \forall \,(\ell ,j)\in {\mathbb {Z}}^{\nu +2}\setminus \{0\}, \ \pi ^\top (\ell ) + j=0 \Big \}. \end{aligned}\nonumber \\ \end{aligned}$$

At each step of the iteration, we solve linear equations of the following form.

### Lemma 3.2

Let $s\geqslant 0$, $\gamma \in (0,1)$ and $\tau > \nu + 1$, Let $g (\,\cdot \,;\omega ,\lambda ) \in H_0^{s+2\tau +1}({\mathbb {T}}^{\nu +2})$ be a quasi-periodic traveling wave. Then, for any $\omega \in \Omega _{\gamma }$ as in (3.6), the linear equation

3.7 $$\begin{aligned} \lambda \, \omega \cdot \partial _{{\varphi }} w({\varphi },x) + \beta \,{\mathtt L}w({\varphi },x) + g({\varphi },x) = 0 \end{aligned}$$

is solved by the quasi-periodic traveling wave $w(\,\cdot ;\omega ,\lambda ) \in H_0^s({\mathbb {T}}^{\nu +2})$, defined as

3.8 $$\begin{aligned} \begin{aligned} w({\varphi },x)&\, = w({\varphi },x;\omega ,\lambda ):= - (\lambda \, \omega \cdot \partial _{{\varphi }} + \beta \,{\mathtt L})^{-1} g({\varphi },x) \\&:= - \sum _{(\ell ,j)\in {\mathbb {Z}}^{\nu +2}, j\ne 0 \atop \pi ^\top (\ell )+j =0} \frac{1}{\textrm{i}\big ( \lambda \,\omega \cdot \ell + \beta \,{\mathtt L}(j)\big )} {\widehat{g}}(\ell ,j) e^{\textrm{i}(\ell \cdot {\varphi }+j\cdot x)}, \end{aligned} \end{aligned}$$

with estimates

3.9 $$\begin{aligned} \begin{aligned}&\Vert w \Vert _{s}^{\textrm{Lip}(\gamma )} \lesssim _{s} \lambda ^{-1} \gamma ^{-1} \Vert g \Vert _{s+2\tau +1}^{\textrm{Lip}(\gamma )} , \\&\quad \inf _{\omega \in \Omega _\gamma } \Vert w(\cdot ; \omega ) \Vert _s \geqslant \tfrac{1}{2} \min \{\lambda ^{-1}, |\beta |^{-1}\} \inf _{\omega \in \Omega _\gamma }\Vert g(\cdot ; \omega ) \Vert _{s-1} . \end{aligned} \end{aligned}$$

Furthermore, we have the measure estimate $|{\mathtt D}{\mathtt C}(\gamma ,\tau ) {\setminus }\Omega _{\gamma }|\lesssim \gamma$, where ${\mathtt D}{\mathtt C}(\gamma , \tau )$ is defined in (3.5).

In addition, if $g({\varphi },x)=\textrm{even}({\varphi },x)$, then $w({\varphi },x)=\textrm{odd}({\varphi },x)$.

### Proof

We write $w({\varphi },x) = \sum _{(\ell , j) \in {\mathbb {Z}}^{\nu +2}, \, j\ne 0} {{\widehat{w}}}(\ell ,j) e^{\textrm{i}(\ell \cdot {\varphi }+ j\cdot x)}$. Then, expanding the equation (3.7) in Fourier, we get

$$\begin{aligned} \textrm{i}\,\big ( \lambda \,\omega \cdot \ell + \beta \, {\mathtt L}(j) \big ) {{\widehat{w}}}(\ell ,j) + {{\widehat{g}}}(\ell ,j)= 0. \end{aligned}$$

Since $g({\varphi },x)$ is a quasi-periodic traveling wave of the form (2.5), we obtain

$$\begin{aligned} {{\widehat{w}}}(\ell ,j):= {\left\{ \begin{array}{ll} \textrm{i}\,\big ( \lambda \,\omega \cdot \ell + \beta \, {\mathtt L}(j) \big )^{-1} {{\widehat{g}}}(\ell ,j) &  \text {if } \ \pi ^\top (\ell ) + j = 0 , \\ 0 &  \text {otherwise}. \end{array}\right. } \end{aligned}$$

We conclude that $w({\varphi },x)$ in (3.8) is indeed a solution of (3.7) and it is a quasi-periodic traveling wave. The upper bound on $\Vert w \Vert _{s}^{\textrm{Lip}(\gamma )}$ in (3.9) follows by Definition 2.1 and since $\omega \in \Omega _{\gamma }$ as in (3.6), whereas the lower bound follows by

the upper bound on the denominator, having $|\omega |\leqslant 2$ for any $\omega \in \Omega _{\gamma } \subset {\mathtt D}{\mathtt C}(\gamma ,\tau )$ (see (3.5)-(3.6)),

$$\begin{aligned} |\lambda \,\omega \cdot \ell +\beta {\mathtt L}(j) | \leqslant \lambda | \omega | |\ell | + |\beta | \leqslant 2 \max \{\lambda ,|\beta |\} |\ell | \leqslant 2 \max \{\lambda ,|\beta |\} {\langle {\ell ,j}\rangle }. \end{aligned}$$

We now prove the measure estimate. For $(\ell ,j)\in {\mathbb {Z}}^{\nu +2}{\setminus }\{0\}$, with $\pi ^\top (\ell ) +j=0$, we define the resonant set

$$\begin{aligned} R_{\ell ,j}:= \Big \{ \omega \in {\mathtt D}{\mathtt C}(\gamma ,\tau ) \,: \, | \lambda \,\omega \cdot \ell + \beta \,{\mathtt L}(j) | < \lambda \gamma {\langle {\ell }\rangle }^{-\tau } \Big \} . \end{aligned}$$

Note that $\ell \ne 0$. Indeed if $\ell = 0$, then also $j = 0$ (by the relation $\pi ^\top (\ell ) +j=0$) but this is not possible since $(\ell , j ) \ne (0, 0)$. Hence, let

$$\begin{aligned} \omega = \frac{\ell }{|\ell |} s + v, \quad v \cdot \ell = 0 \end{aligned}$$

and set

$$\begin{aligned} z(s):=\lambda \,\omega \cdot \ell + \beta \,{\mathtt L}(j) = \lambda |\ell | s + \beta {\mathtt L}(j). \end{aligned}$$

Since $|\partial _s z(s)| \geqslant \lambda |\ell |$, one gets that

$$\begin{aligned} \Big | \Big \{ s: \omega = \frac{\ell }{|\ell |} s + v \in R_{\ell , j} \Big \} \Big | \lesssim \lambda \gamma \langle \ell \rangle ^{- (\tau + 1)} \end{aligned}$$

and hence by Fubini, one obtains that $|R_{\ell , j}| \lesssim \lambda \gamma \langle \ell \rangle ^{- (\tau + 1)}$. We then compute

$$\begin{aligned} \begin{aligned} | {\mathtt D}{\mathtt C}(\gamma ,\tau ) \setminus \Omega _{\gamma } |&= \Big | \bigcup _{(\ell ,j)\in {\mathbb {Z}}^{\nu +2}\setminus \{0\} \atop \pi ^\top (\ell )+j=0} R_{\ell ,j} \Big | = \Big | \bigcup _{{\begin{array}{c} \ell \in {\mathbb {Z}}^\nu \setminus \{0\} \\ |j| \lesssim |\ell | \end{array}}} R_{\ell ,j } \Big | \\&\lesssim \sum _{{\begin{array}{c} \ell \ne 0 \\ |j| \lesssim |\ell | \end{array}}} \frac{\lambda \gamma }{\langle \ell \rangle ^{\tau + 1}} \lesssim \sum _{\ell \ne 0} \frac{\lambda \gamma |\ell |^2}{\langle \ell \rangle ^{\tau + 1}} \lesssim \sum _{\ell \ne 0} \frac{\lambda \gamma }{\langle \ell \rangle ^{\tau - 1}} , \end{aligned} \end{aligned}$$

where the last inequality holds since $\tau > \nu + 1$.

Finally, if we assume $g({\varphi },x)=\textrm{even}({\varphi },x)$, then we have that $w({\varphi },x)=\textrm{odd}({\varphi },x)$, using that $\lambda \,\omega \cdot \partial _{{\varphi }}+\beta \,{\mathtt L}$ is reversible, by (1.5), Lemma 2.9 and Definition 2.8. This concludes the proof. $\square$

We are now ready to construct explicitly the approximate solution of (3.1).

### Proposition 3.3

(**Approximate solution**). Let $N\in {\mathbb {N}}_0$. Let $s\geqslant s_0$, $\gamma \in (0,1)$ and $\tau > \nu + 1$. For any $\omega \in \Omega _{\gamma }$, there exists a quasi-periodic traveling wave $v_{\textrm{app},N}({\varphi },x) = v_{\textrm{app},N}({\varphi },x; \omega ,\lambda ) \in H_0^s({\mathbb {T}}^{\nu +2})$ of the form (3.4) satisfying

3.10 $$\begin{aligned} {\mathcal {F}}(v_{\textrm{app},N}({\varphi },x)) = q_{N}({\varphi },x), \end{aligned}$$

where the remainder $q_{N}({\varphi },x) = q_{N}({\varphi },x,\omega ,\lambda ) \in H_0^s({\mathbb {T}}^{\nu +2})$ is a quasi-periodic traveling wave as well. Moreover, there exists $\overline{\lambda }=\overline{\lambda }(s,N,\tau ,\beta )\gg 1$ large enough such that, defining the constants

$$\begin{aligned} \begin{aligned}&\kappa _{N-\frac{1}{2}}(\tau ):= \kappa _{N}(\tau ) -1 , \quad \kappa _{N}(\tau ):= 2(N+1)(\tau +1) , \end{aligned} \end{aligned}$$

and assuming, for a given ${\mathtt c}\in (0,\frac{1}{3}(2-\alpha ))$,

3.11 $$\begin{aligned} \lambda \geqslant \overline{\lambda } \geqslant \max \{ 1,|\beta |\}, \quad \gamma = \lambda ^{-{\mathtt c}} , \quad \theta := \alpha -1 +{\mathtt c}, \end{aligned}$$

the following estimates hold, for any $s\geqslant s_0$:

3.12 $$\begin{aligned}&\Vert v_{\textrm{app},N} -v_{\textrm{app},N-1} \Vert _s^{\textrm{Lip}(\gamma )} \lesssim _{s,N} \lambda ^{N(\alpha -2(1-{\mathtt c}) )} \Vert f \Vert _{s+\kappa _{N-\frac{1}{2}}(\tau )} \,; \end{aligned}$$

3.13 $$\begin{aligned}&\Vert v_{\textrm{app},N} \Vert _s^{\textrm{Lip}(\gamma )} \lesssim _{s,N} \Vert f \Vert _{s+\kappa _{N-\frac{1}{2}}(\tau )}\,, \quad \inf _{\omega \in \Omega _\gamma } \Vert v_{\textrm{app},N}(\cdot ; \omega ) \Vert _s \gtrsim _{s, N} \lambda ^{-{\mathtt c}} \Vert f \Vert _{s-1} \,; \end{aligned}$$

3.14 $$\begin{aligned}&\Vert q_{N} \Vert _{s}^{\textrm{Lip}(\gamma )} \lesssim _{s,N} \lambda ^{(N+1)(\alpha -2(1-{\mathtt c}))+1-{\mathtt c}} \Vert f\Vert _{s+\kappa _{N}(\tau )}\,. \end{aligned}$$

In particular, assuming the symmetry condition (3.2) on the forcing term $f({\varphi }, x)$ in (1.9), we have that $v_{\textrm{app},N}({\varphi },x)= \textrm{odd}({\varphi },x)$ and $q_N({\varphi },x)=\textrm{even}({\varphi },x)$. Finally, suppose that

3.15 $$\begin{aligned} N >\frac{\alpha -(1-{\mathtt c})}{2(1-{\mathtt c})-\alpha }. \end{aligned}$$

Then, $v_{\textrm{app},N}({\varphi },x)$ approximately solves (3.1), namely the term on the right-hand side of (3.14) becomes perturbatively small for sufficiently large $\lambda \gg 1$.

### Proof

We argue by induction on $N\in {\mathbb {N}}_0$. Let $N=0$. Let $v_{\textrm{app},0}({\varphi },x) = v_0({\varphi },x)$ be a solution of the equation

3.16 $$\begin{aligned} (\lambda \,\omega \cdot \partial _{{\varphi }} + \beta \, {\mathtt L}) v_{0}({\varphi },x) - \lambda ^{\alpha -\theta } f({\varphi },x) = 0. \end{aligned}$$

By Lemma 3.2, $v_0({\varphi },x)$ is a well-defined quasi-periodic traveling wave, with estimates, recalling (3.11),

3.17 $$\begin{aligned} \begin{aligned}&\Vert v_{0} \Vert _{s}^{\textrm{Lip}(\gamma )} \lesssim _{s} \lambda ^{-1} \gamma ^{-1} \Vert \lambda ^{\alpha -\theta } f \Vert _{s+2\tau +1} = \gamma ^{-1}\lambda ^{-{\mathtt c}} \Vert f\Vert _{s+2\tau +1} = \Vert f\Vert _{s+2\tau +1} , \\&\quad \inf _{\omega \in \Omega _\gamma }\Vert v_{0}(\cdot ; \omega ) \Vert _{s}\geqslant \frac{1}{2} \min \{\lambda ^{-1}, |\beta |^{-1} \} \, \Vert \lambda ^{\alpha -\theta } f \Vert _{s-1} \geqslant \frac{1}{2} \lambda ^{-{\mathtt c}} \Vert f \Vert _{s-1}, \end{aligned} \end{aligned}$$

which proves (3.12) with $N=0$, as well as (3.13), having $\kappa _{-\frac{1}{2}}(\tau )=2\tau +1$. Using (3.16), we have that (3.10) holds at $N=0$ with $q_0({\varphi },x)$ defined by

3.18 $$\begin{aligned} q_0({\varphi },x):= \lambda ^{\theta }{\mathcal {N}}(v_0({\varphi },x),v_0({\varphi },x)) . \end{aligned}$$

By (3.1), (1.8), Lemma 2.2, Lemma 3.1-(*iii*), estimate (3.17) and (3.11), we obtain that (3.18) has zero average in space and satisfies (3.14) with $N=0$, having $\kappa _{0}(\tau )= 2(\tau +1)>0$. The claim that $v_0({\varphi },x)=\textrm{odd}({\varphi },x)$ follows by assuming (3.2) and by Lemma 3.2, whereas $q_0({\varphi },x)=\textrm{even}({\varphi },x)$ follows by (3.18), (3.1) and Lemma 3.1-(*iii*).

We now assume by induction that (3.10) holds at $N\in {\mathbb {N}}_0$ with estimates (3.12)-(3.13) and we prove the claim at $N+1$. We insert (3.4) into (3.10), both with $N\rightsquigarrow N+1$. Since $v_{\textrm{app},N}$ satisfies (3.10), we look for $v_{N+1}$ satisfying

3.19 $$\begin{aligned} \begin{aligned}&\big (\lambda \, \omega \cdot \partial _{{\varphi }} + \beta \, {\mathtt L}\big )v_{N+1} + \lambda ^{\theta }\big ({\mathcal {N}}(v_{\textrm{app},N} + v_{N+1},v_{\textrm{app},N} + v_{N+1} ) \\&\quad - {\mathcal {N}}(v_{\textrm{app},N},v_{\textrm{app},N}) \big )+q_{N}({\varphi },x) = q_{N+1}({\varphi },x), \end{aligned} \end{aligned}$$

with $q_{N+1}({\varphi },x)$ to be determined. In particular, we choose $v_{N+1}({\varphi },x)$ to be a solution to

3.20 $$\begin{aligned} \big (\lambda \, \omega \cdot \partial _{{\varphi }} + \beta \, {\mathtt L}\big )v_{N+1}({\varphi },x)+q_{N}({\varphi },x) =0. \end{aligned}$$

Then, by Lemma 3.2, estimate (3.14) and (3.11), we obtain, for any $s\geqslant s_0$

3.21 $$\begin{aligned} \begin{aligned} \Vert v_{N+1} \Vert _{s}^{\textrm{Lip}(\gamma )}&\lesssim _{s} \lambda ^{-1} \gamma ^{-1} \Vert q_{N} \Vert _{s+2\tau + 1}^{\textrm{Lip}(\gamma )} \\&\lesssim _{s} \lambda ^{-1+{\mathtt c}} \lambda ^{(N+1)(\alpha -2(1-{\mathtt c})) +1-{\mathtt c}} \Vert f \Vert _{s+ \kappa _{N}(\tau )+2\tau +1}^{\textrm{Lip}(\gamma )} , \end{aligned} \end{aligned}$$

which proves (3.12) at the step $N+1$, setting $\kappa _{N+1-\frac{1}{2}}(\tau ):= \kappa _{N}(\tau )+2\tau +1$.

We now prove (3.13) at the step $N+1$. By (3.4), estimate (3.12) at the step $N+1$, the induction assumption on (3.13), (3.11) and assuming $\lambda \gg 1$ large enough, we have

$$\begin{aligned} \begin{aligned} \Vert v_{\textrm{app},N+1} \Vert _{s}^{\textrm{Lip}(\gamma )}&\leqslant \Vert v_{\textrm{app},N} \Vert _{s}^{\textrm{Lip}(\gamma )} + \Vert v_{\textrm{app},N+1} - v_{\textrm{app},N} \Vert _{s}^{\textrm{Lip}(\gamma )} \\&\lesssim _{s,N} \big ( 1 + \lambda ^{(N+1)(\alpha -2(1-{\mathtt c}))} \big ) \Vert f \Vert _{s+\kappa _{N-\frac{1}{2}}(\tau )}^{\textrm{Lip}(\gamma )} \lesssim _{s,N} \Vert f \Vert _{s+\kappa _{N-\frac{1}{2}}(\tau )}^{\textrm{Lip}(\gamma )}, \end{aligned} \end{aligned}$$

which proved the upper bound in (3.13) at the step $N+1$, whereas

$$\begin{aligned} \begin{aligned} \inf _{\omega \in \Omega _\gamma }\Vert v_{\textrm{app},N+1}(\cdot ; \omega ) \Vert _{s}&\geqslant \inf _{\omega \in \Omega _\gamma } \Vert v_{\textrm{app},N}(\cdot ; \omega ) \Vert _{s} - \Vert v_{\textrm{app},N+1} - v_{\textrm{app},N}\Vert _{s}^{\textrm{Lip}(\gamma )} \\&\geqslant C_{s, N}\lambda ^{-{\mathtt c}} \Vert f \Vert _{s-1} - K_{s, N} \lambda ^{(N+1)(\alpha -2(1-{\mathtt c}))} \Vert f\Vert _{s+\kappa _{N+1-\frac{1}{2}}} \\&\geqslant \frac{C_{s, N}}{2} \lambda ^{-{\mathtt c}} \Vert f \Vert _{s-1} , \end{aligned} \end{aligned}$$

(for some positive constants $C_{s, N}, K_{s, N} > 0$), assuming $\lambda \geqslant {\overline{\lambda }} \gg 1$ large enough such that

3.22 $$\begin{aligned} \lambda ^{(N+1)(\alpha -2(1-{\mathtt c})) + {\mathtt c}}\leqslant \frac{C_{s,N}}{2 K_{s,N}}\frac{ \Vert f \Vert _{s-1} }{ \Vert f\Vert _{s+\kappa _{N+1-\frac{1}{2}}} }, \end{aligned}$$

which shows the lower bound in (3.13) at the step $N+1$, as claimed. Note that we can impose (3.22) for $\lambda \gg 1$ because ${\mathtt c}<\frac{1}{3}(2-\alpha )$ implies $2(1-{\mathtt c})-\alpha> {\mathtt c}> \frac{{\mathtt c}}{N+1}$.

Furthermore, since $q_{N}$ and $v_{\textrm{app},N}$ are quasi-periodic traveling waves by induction assumption, by Lemma 3.2 we have that $v_{N+1}$ and $v_{\textrm{app},N+1}$ are quasi-periodic traveling waves as well.

It remains to define $q_{N+1}({\varphi },x)$ and to prove (3.14) at the step $N+1$. By inserting (3.20) into (3.19), the identity holds by defining

3.23 $$\begin{aligned} \begin{aligned} q_{N+1}&:=\lambda ^{\theta }\big ({\mathcal {N}}(v_{\textrm{app},N} + v_{N+1},v_{\textrm{app},N} + v_{N+1} ) - {\mathcal {N}}(v_{\textrm{app},N},v_{\textrm{app},N}) \big ) \\&\, = \lambda ^{\alpha -1+{\mathtt c}} \big ( {\mathcal {N}}(v_{\textrm{app},N}, v_{N+1} ) + {\mathcal {N}}( v_{N+1},v_{\textrm{app},N} ) + {\mathcal {N}}( v_{N+1}, v_{N+1} ) \big ), \end{aligned} \end{aligned}$$

where we used (3.11) and the bilinearity of ${\mathcal {N}}(\,\cdot \,, \,\cdot \,)$. By Lemma 3.1-(*iii*), we deduce that $q_{N+1}({\varphi },x)$ has zero average in space. By (3.1), (1.8), Lemma 2.2, and estimates (3.13), (3.21), we estimate (3.23) by

$$\begin{aligned} \begin{aligned} \Vert q_{N+1} \Vert _{s}^{\textrm{Lip}(\gamma )}&\lesssim _{s} \lambda ^{\alpha -1+{\mathtt c}} \Big ( \Vert v_{\textrm{app},N} \Vert _{s-1}^{\textrm{Lip}(\gamma )} \Vert v_{N+1} \Vert _{s_0+1}^{\textrm{Lip}(\gamma )} + \Vert v_{\textrm{app},N} \Vert _{s_0-1}^{\textrm{Lip}(\gamma )} \Vert v_{N+1} \Vert _{s+1}^{\textrm{Lip}(\gamma )} \\&\qquad \qquad \quad \ \ \Vert v_{N+1} \Vert _{s-1}^{\textrm{Lip}(\gamma )} \Vert v_{\textrm{app},N} \Vert _{s_0+1}^{\textrm{Lip}(\gamma )} + \Vert v_{N+1} \Vert _{s_0-1}^{\textrm{Lip}(\gamma )} \Vert v_{\textrm{app},N} \Vert _{s+1}^{\textrm{Lip}(\gamma )} \\&\qquad \qquad \quad \ \ \Vert v_{N+1} \Vert _{s-1}^{\textrm{Lip}(\gamma )} \Vert v_{N+1} \Vert _{s_0+1}^{\textrm{Lip}(\gamma )} + \Vert v_{N+1} \Vert _{s_0-1}^{\textrm{Lip}(\gamma )} \Vert v_{N+1} \Vert _{s+1}^{\textrm{Lip}(\gamma )} \Big ) \\&\lesssim _{s,N} \lambda ^{(N+2)(\alpha -2(1-{\mathtt c})) + 1-{\mathtt c}} \big ( 1+ \lambda ^{(N+1)(\alpha -2(1-{\mathtt c}))} \big ) \Vert f \Vert _{s+\kappa _{N+1-\frac{1}{2}}(\tau )+1} \\&\lesssim _{s,N} \lambda ^{(N+2)(\alpha -2(1-{\mathtt c}))+1-{\mathtt c}} \Vert f \Vert _{s+\kappa _{N+1-\frac{1}{2}}(\tau )+1} , \end{aligned} \end{aligned}$$

which yields (3.14) at the step $N+1$, with $\kappa _{N+1}(\tau ):= \kappa _{N+1-\frac{1}{2}}(\tau )+1$. Furthermore, since $v_{\textrm{app},N}$ is a quasi-periodic traveling wave by induction assumption and we showed that $v_{N+1}$ is a quasi-periodic traveling wave, it follows that $q_{N+1}$ in (3.23) is a quasi-periodic traveling wave as well. Finally, the claim that $v_{\textrm{app},N+1}({\varphi },x)=\textrm{odd}({\varphi },x)$ follows from the fact that $q_N({\varphi },x)=\textrm{even}({\varphi },x)$, $v_{\textrm{app},N}({\varphi },x)=\textrm{odd}({\varphi },x)$ by induction assumption and by Lemma 3.2, whereas the $q_{N+1}({\varphi },x)=\textrm{even}({\varphi },x)$ follows by (3.23), (3.1), (1.8) and Lemma 2.9. This concludes the proof of the claim and of the proposition. $\square$

## The Linearized Operator

Once the approximate solution $v_{\textrm{app},N}({\varphi },x)$ with respect to $\lambda \gg 1$ (as constructed in Proposition 3.3, see (3.13) and (3.14)), is obtained, we proceed to study the linearization of equation (3.1) at any approximate solution of the Nash-Moser iteration (see Sect. 7). This approximate solution takes the form

4.1 $$\begin{aligned} w= v_{\textrm{app},N}+ g \in {{\mathcal {C}}}^\infty ({\mathbb {T}}^\nu \times {\mathbb {T}}^2) , \end{aligned}$$

satisfying the ansatz

4.2 $$\begin{aligned} \Vert w \Vert _{s_0 + \sigma }^{\textrm{Lip}(\gamma )} =\Vert w \Vert _{s_0 + \sigma ,\Lambda _{o}}^{\textrm{Lip}(\gamma )} \leqslant C_0, \quad \begin{array}{ll} \text {for some constants } \sigma ,\, C_0 \gg 0 \text { large enough } \\ \text {and for a given } \Lambda _{o}\subseteq \Omega _{\gamma } \subset {\mathbb {R}}^\nu , \end{array} \end{aligned}$$

recalling the definition of $\Omega _{\gamma }$ in (3.6). The set $\Lambda _{o}=\Lambda _{o}(w)$ will change at each step of the Nash-Moser iteration in Proposition 7.3. Recalling (3.3), the linearized operator is given by

4.3 $$\begin{aligned} {\mathcal {L}}:= \textrm{d}_{v} {\mathcal {F}}( w):= \lambda \, \omega \cdot \partial _{{\varphi }} + \beta \,{\mathtt L}+ \lambda ^{\theta }\textbf{a}_{0}\cdot \nabla + \lambda ^{\theta } {{\mathcal {E}}_{0}}, \end{aligned}$$

where

4.4 $$\begin{aligned} \textbf{a}_{0}:= {\mathfrak {B}}\big [ w \big ] , \quad {\mathcal {E}}_{0}[h]:= \nabla w \cdot {\mathfrak {B}}[h] \end{aligned}$$

and

4.5 $$\begin{aligned} \theta := \alpha -1+{\mathtt c}, \quad \text {for some} \ \ {\mathtt c}\in (0,\tfrac{1}{3}(2-\alpha )). \end{aligned}$$

The range for the parameter ${\mathtt c}>0$ ensures that, for any fixed $\alpha \in (1,2)$,

4.6 $$\begin{aligned} \theta -1< \theta - 1 + {\mathtt c}< 0 . \end{aligned}$$

The parameter ${\mathtt c}$ will be fixed arbitrarily small enough in Proposition 7.3, independently of the step of the Nash-Moser iteration. In particular, it will guarantee that

4.7 $$\begin{aligned} \lambda ^{\theta -1} \gamma ^{-1} \ll 1 . \end{aligned}$$

By the ansatz (4.2) and by the definitions (4.4), we get that

$$\begin{aligned} \Vert \textbf{a}_{0} \Vert _{s} \lesssim _s \Vert w \Vert _{s-1} , \quad \forall s \geqslant s_0 , \end{aligned}$$

and, for any $s\geqslant s_0$, ${\mathcal {E}}_{0} \in {{\mathcal {O}}}{{\mathcal {P}}}{{\mathcal {M}}}_{s}^{- 1}$, with the estimate

$$\begin{aligned} |{\mathcal {E}}_{0}|_{- 1, s, \alpha } \lesssim _{s, \alpha } \Vert w \Vert _{s+1} , \quad \forall s \geqslant s_0, \quad \alpha \in {\mathbb {N}}. \end{aligned}$$

By the definition of $\textbf{a}_{0}$ in (4.4) and the definition of the Biot-Savart operator ${\mathfrak {B}}$ in (1.8), we clearly have that

4.8 $$\begin{aligned} {\langle {\textbf{a}_{0}}\rangle }_{x}:=\frac{1}{(2\pi )^2}\int _{{\mathbb {T}}^2}\textbf{a}_{0}({\varphi },x) \,\textrm{d}{x} = 0, \quad \textrm{div}(\textbf{a}_{0}):= \nabla \cdot \textbf{a}_{0} = 0 . \end{aligned}$$

Moreover, using also that $\textrm{div} (\nabla ^\perp h) = 0$ for any *h*, the operators $\textbf{a}_{0} \cdot \nabla$, ${\mathcal {E}}_{0}$ and ${\mathcal L}$ leave invariant the subspace of zero average function, with

4.9 $$\begin{aligned} \begin{aligned}&[\Pi _0^\bot , \textbf{a}_{0} \cdot \nabla ] = 0, \quad [\Pi _0^\bot , {\mathcal {E}}_{0} ] = 0, \\&\quad \textbf{a}_{0} \cdot \nabla \Pi _0 = \Pi _0 \textbf{a}_{0} \cdot \nabla = 0, \quad {\mathcal {E}}_{0} \Pi _0 = \Pi _0 {\mathcal {E}}_{0} = 0 ,\\&\quad [\Pi _0^\bot , {{\mathcal {L}}}] = 0, \quad \Pi _0 {{\mathcal {L}}}= {{\mathcal {L}}} \Pi _0 = 0, \end{aligned} \end{aligned}$$

implying that

4.10 $$\begin{aligned} \textbf{a}_{0} \cdot \nabla = \Pi _0^\bot \textbf{a}_{0} \cdot \nabla \Pi _0^\bot , \quad {\mathcal {E}}_{0} = \Pi _0^\bot {\mathcal {E}}_{0} \Pi _0^\bot , \quad {\mathcal L} = \Pi _0^\bot {{\mathcal {L}}} \Pi _0^\bot . \end{aligned}$$

We always work on the space of zero average functions and we shall preserve this invariance along the whole paper.

## Normal Form Reduction

We now present the step-by-step scheme of reduction for the operator ${{\mathcal {L}}}$ defined in (4.3).

### Reduction of the transport

We consider the composition operator

5.1 $$\begin{aligned} ({\mathcal {B}}u)({\varphi },x):= h ({\varphi }, x+ {\varvec{\beta }}({\varphi },x)) , \quad ({\mathcal {B}}^{-1} h ) ({\varphi },y):= h ({\varphi }, y + \breve{{\varvec{\beta }}}({\varphi },y)), \end{aligned}$$

induced by a ${\varphi }$-dependent family of diffeomorphisms of the torus

$$\begin{aligned} {\mathbb {T}}^2 \rightarrow {\mathbb {T}}^2, \quad x \mapsto y:= x + {\varvec{\beta }}({\varphi },x) , \end{aligned}$$

with inverse $y \mapsto x = y + \breve{{\varvec{\beta }}}({\varphi },y)$ for $\Vert {\varvec{\beta }}\Vert _{s}$ sufficiently small and where ${\varvec{\beta }}({\varphi },x)$, $\breve{{\varvec{\beta }}}({\varphi },y)$ are quasi-periodic traveling wave functions.

We need to reduce to constant coefficients the highest order operator which is a transport operator of the form

5.2 $$\begin{aligned} \begin{aligned} {\mathcal {T}}&:= \lambda \, \omega \cdot \partial _{{\varphi }} + \lambda ^{\theta } \textbf{a}_{0} \cdot \nabla = \lambda \, {\widetilde{{\mathcal {T}}}}, \\ {\widetilde{{\mathcal {T}}}}&:= \omega \cdot \partial _\varphi + \textbf{b}(\varphi , x)\cdot \nabla , \quad \textbf{b}(\varphi , x):= \varepsilon \textbf{a}_0(\varphi , x), \quad \varepsilon := \lambda ^{\theta -1}. \end{aligned} \end{aligned}$$

Note that, for $\lambda \gg 1$, by(4.5)-(4.6), one has that $\varepsilon = \lambda ^{\theta -1} \ll 1$ and the quasi-periodic traveling wave function $\textbf{b}(\varphi , x)$ satisfies the estimate

5.3 $$\begin{aligned} \begin{aligned}&\Vert \textbf{b} \Vert _s \lesssim _s \lambda ^{\theta -1} \Vert w \Vert _{s-1}, \quad \forall s \geqslant s_0, \\&\quad \Vert \Delta _{12} \textbf{b} \Vert _{s_1} \lesssim _{s_1} \lambda ^{\theta -1} \Vert w_1 - w_2 \Vert _{s_1-1} , \quad s_1 \geqslant s_0, \end{aligned} \end{aligned}$$

where *w* is given in (4.1), the notation $\Delta _{12}$ is given in (2.2), and $w_1, w_2$ satisfy the ansatz (4.2). We consider the set of Diophantine non-resonance conditions as defined in (3.5).

We have the following result.

#### Proposition 5.1

(**Straightening of the transport operator** ${\mathcal {T}}$). Let $\gamma \in (0,1)$ and $\tau >\nu -1$. There exists $\sigma := \sigma (\tau , \nu ) > 0$ large enough such that, for any $S > s_0 + \sigma$, there exist $\delta := \delta (S, \tau , \nu ) \in (0, 1)$ small enough and $\tau _{1}=\tau _{1}(\tau ,\nu )>0$ such that, if (4.2), (5.3) hold and

5.4 $$\begin{aligned} N_0^{\tau _{1}}\varepsilon \gamma ^{- 1} = N_0^{\tau _{1}} \lambda ^{\theta -1} \gamma ^{-1} \leqslant \delta , \end{aligned}$$

is fulfilled, then the following holds. There exists an invertible diffeomorphism ${\mathbb {T}}^2 \rightarrow {\mathbb {T}}^2$, $x \mapsto x + {\varvec{\beta }}(\varphi , x; \omega )$ with inverse $y \mapsto y + \breve{\varvec{\beta }}(\varphi , y; \omega )$, defined for all $\omega \in {\mathtt D}{\mathtt C}(2\gamma , \tau )\cap \Lambda _{o}$, with the set given in (3.5),

satisfying, for any $s_0\leqslant s \leqslant S - \sigma$,

5.5 $$\begin{aligned} \Vert {\varvec{\beta }}\Vert _s^{\textrm{Lip}(\gamma )}, \Vert \breve{\varvec{\beta }}\Vert _{s}^{\textrm{Lip}(\gamma )} \lesssim _{s} N_0^{2\tau +1} \varepsilon \gamma ^{- 1} \Vert w \Vert _{s + \sigma }^{\textrm{Lip}(\gamma )} , \end{aligned}$$

such that one gets the conjugation

5.6 $$\begin{aligned} {{\mathcal {B}}}^{- 1} {{\mathcal {T}}} {{\mathcal {B}}} = \lambda \, \omega \cdot \partial _\varphi , \end{aligned}$$

where the invertible maps ${\mathcal {B}}$, ${\mathcal {B}}^{-1}$ are defined in (5.1), satisfying the estimates, for any $s\in [s_0,S-\sigma ]$,

5.7 $$\begin{aligned} \begin{aligned}&\Vert {{\mathcal {B}}}^{\pm 1} h\Vert _s^{\textrm{Lip}(\gamma )} \lesssim _{s} \Vert h \Vert _s^{\textrm{Lip}(\gamma )} + \Vert w \Vert _{s + \sigma }^{\textrm{Lip}(\gamma )} \Vert h \Vert _{s_0}^{\textrm{Lip}(\gamma )}, \\&\quad \Vert ({{\mathcal {B}}}^{\pm 1} - \textrm{Id}) h\Vert _s^{\textrm{Lip}(\gamma )} \lesssim _{s} N_0^{2\tau +1} \varepsilon \gamma ^{- 1} \big ( \Vert w \Vert _{s_0 + \sigma }^{\textrm{Lip}(\gamma )} \Vert h \Vert _{s + 1}^{\textrm{Lip}(\gamma )} + \Vert w \Vert _{s + \sigma }^{\textrm{Lip}(\gamma )} \Vert h \Vert _{s_0 + 1}^{\textrm{Lip}(\gamma )} \big ) . \end{aligned}\nonumber \\ \end{aligned}$$

Let $s_1 \geqslant s_0$ and assume that $w_1, w_2$ satisfy (4.2) with $\sigma _{0} \geqslant s_1 + \sigma$. Then, for any $\omega \in {\mathtt D}{\mathtt C}(2\gamma , \tau )\cap \Lambda _{o}(w_1)\cap \Lambda _{o}(w_2)$, with $w_1,w_2$ satisfying (4.2) and recalling the notation $\Delta _{12}$ in (2.2), one has

5.8 $$\begin{aligned} \begin{aligned}&\Vert \Delta _{12} {\varvec{\beta }}\Vert _{s_1} ,\, \Vert \Delta _{12} \breve{\varvec{\beta }}\Vert _{s_1} \lesssim _{s_1} N_0^{2\tau +1}\varepsilon \gamma ^{- 1} \Vert w_1 - w_2 \Vert _{s_1 + \sigma }, \\&\quad \Vert \Delta _{12} {{\mathcal {B}}}^{\pm 1} h \Vert _{s_1} \lesssim _{s_1} N_0^{2\tau +1} \varepsilon \gamma ^{- 1} \Vert w_1 -w_2 \Vert _{s_1 +\sigma } \Vert h \Vert _{s_1 + 1}. \end{aligned} \end{aligned}$$

Furthermore, ${\varvec{\beta }},\breve{{\varvec{\beta }}}$ are quasi-periodic traveling waves, and the related maps ${{\mathcal {B}}}, {{\mathcal {B}}}^{- 1}$ are momentum preserving (see Definition 2.10). In addition, if we assume $w({\varphi },x)= \textrm{odd}({\varphi },x)$, then ${\varvec{\beta }},\breve{{\varvec{\beta }}}$ are $\textrm{odd}(\varphi ,x)$ and the related maps ${{\mathcal {B}}}, {{\mathcal {B}}}^{- 1}$ are reversibility preserving (see Definition 2.8).

In order to prove Proposition 5.1, we follow [7] and [9]. First we show the following iterative lemma. We fix the constants

5.9 $$\begin{aligned} \begin{aligned}&N_0 >0 , \quad N_{-1}:= 1, \quad \chi := 3/2, \quad N_{n}:= N_0^{\chi ^n}, \quad n \in {\mathbb {N}}_0, \\&\quad {\mathfrak {a}}:= 6 \tau +8, \quad {\mathfrak {b}}:= {\mathfrak {a}}+1 . \end{aligned} \end{aligned}$$

#### Lemma 5.2

Let $\gamma \in (0,1)$ and $\tau >\nu -1$. There exists $\sigma := \sigma (\tau , \nu ) > 0$ large enough such that, for any $S > s_0 + \sigma$, there exist $\delta := \delta (S, \tau , \nu ) \in (0, 1)$ small enough, $N_0=N_0(S,\tau ,\nu )>0$ large enough and $\tau _{1}=\tau _{1}(\tau ,\nu )>0$ such that, if (4.2), (5.3) and (5.4) hold for $\sigma _{0}\geqslant s_0 +\sigma$, the following holds for $n\geqslant 0$:

There exists a linear operator

5.10 $$\begin{aligned} \begin{aligned} {\mathcal {T}}_{n}:= \lambda \,{\widetilde{{\mathcal {T}}}}_{n}, \quad {\widetilde{{\mathcal {T}}}}_{n}:= \omega \cdot \partial _{{\varphi }} + {\mathtt m}_{n} \cdot \nabla + \textbf{b}_{n}({\varphi },x) \cdot \nabla , \end{aligned} \end{aligned}$$

defined for all $\omega \in {\mathcal {O}}_{n}^{\gamma }$, where we define ${\mathcal {O}}_{0}^{\gamma }:=\Lambda _{o}$ for $n=0$ and, for $n\geqslant 1$,

5.11 $$\begin{aligned} \begin{aligned}&{\mathcal {O}}_{n}^{\gamma }:= \Big \{ \omega \in {\mathcal {O}}_{n-1}^\gamma \,: \, |\omega \cdot \ell + {\mathtt m}_{n-1}\cdot j | \geqslant {\gamma }\,{{\langle {\ell }\rangle }^{-\tau }} , \ \ \forall \,(\ell ,j)\in {\mathbb {Z}}^{\nu +2}, \\&\quad \ \ 0<| \ell | \leqslant N_{n-1} , \ \ \pi ^\top (\ell ) + j = 0 \Big \} , \end{aligned} \end{aligned}$$

and $\textbf{b}_{n}({\varphi },x)$, ${\mathtt m}_{n}$ satisfy the following properties. The function $\textbf{b}_{n}({\varphi },x)$ is a quasi-periodic traveling wave, with estimates, for any $s \in [s_0,S-\sigma ]$,

5.12 $$\begin{aligned} \Vert \textbf{b}_{n} \Vert _{s}^{\textrm{Lip}(\gamma )} \leqslant C(s,{\mathfrak {b}}) N_{n-1}^{-{\mathfrak {a}}} \Vert \textbf{b}\Vert _{s+\sigma } , \quad \Vert \textbf{b}_{n} \Vert _{s+{\mathfrak {b}}}^{\textrm{Lip}(\gamma )} \leqslant C(s,{\mathfrak {b}}) N_{n-1} \Vert \textbf{b}\Vert _{s+\sigma }, \end{aligned}$$

for some constant $C(s,{\mathfrak {b}})>0$ monotone increasing with $[s_0,S-\sigma ]$, and ${\mathtt m}_{n}$ is a real constant satisfying

5.13 $$\begin{aligned} | {\mathtt m}_{n} |^{\textrm{Lip}(\gamma )} \leqslant 2 \Vert \textbf{b}\Vert _{s_0}^{\textrm{Lip}(\gamma )} , \quad |{\mathtt m}_{n} - {\mathtt m}_{n-1} |^{\textrm{Lip}(\gamma )} \leqslant C(s_0,{\mathfrak {b}}) N_{n-2}^{-{\mathfrak {a}}} \Vert \textbf{b}\Vert _{s_0+{\mathfrak {b}}}^{\textrm{Lip}(\gamma )} , \ \ n\geqslant 2. \nonumber \\ \end{aligned}$$

For $n\geqslant 1$, there exists an invertible diffeomorphism of the torus ${\mathbb {T}}^2\rightarrow {\mathbb {T}}^2$, $x \mapsto x + {\varvec{\beta }}_{n-1}({\varphi },x)$, with inverse $y \mapsto y + \breve{{\varvec{\beta }}}_{n-1}({\varphi },x)$, such that, for any $s\in [s_0,S - \sigma ]$,

5.14 $$\begin{aligned} \begin{aligned}&\Vert {\varvec{\beta }}_{n-1} \Vert _{s}^{\textrm{Lip}(\gamma )}, \ \Vert \breve{{\varvec{\beta }}}_{n-1} \Vert _{s}^{\textrm{Lip}(\gamma )} \lesssim _{s} N_{n-1}^{2\tau +1} N_{n-1}^{-{\mathfrak {a}}} \varepsilon \gamma ^{-1} \Vert w \Vert _{s+\sigma }^{\textrm{Lip}(\gamma )} , \\&\quad \Vert {\varvec{\beta }}_{n-1} \Vert _{s+{\mathfrak {b}}}^{\textrm{Lip}(\gamma )} , \ \Vert \breve{{\varvec{\beta }}}_{n-1} \Vert _{s+{\mathfrak {b}}}^{\textrm{Lip}(\gamma )} \lesssim _{s} N_{n-1}^{2\tau +1} N_{n-1} \varepsilon \gamma ^{-1} \Vert w \Vert _{s+\sigma }^{\textrm{Lip}(\gamma )} . \end{aligned} \end{aligned}$$

The operators

$$\begin{aligned} ({\mathcal {B}}_{n-1} u)({\varphi },x):= h ({\varphi }, x+ {\varvec{\beta }}_{n-1}({\varphi },x)) , \quad ({\mathcal {B}}_{n-1}^{-1} h ) ({\varphi },y):= h ({\varphi }, y + \breve{{\varvec{\beta }}}_{n-1}({\varphi },y)), \end{aligned}$$

satisfy, for any $\omega \in {\mathcal {O}}_{n}^{\gamma }$, the conjugation

5.15 $$\begin{aligned} {\mathcal {T}}_{n} = {\mathcal {B}}_{n-1}^{-1} {\mathcal {T}}_{n-1} {\mathcal {B}}_{n-1} . \end{aligned}$$

Furthermore, ${\varvec{\beta }}_{n-1},\breve{{\varvec{\beta }}}_{n-1}$ are quasi-periodic traveling waves, and the related maps ${{\mathcal {B}}}_{n-1}, {\mathcal B}_{n-1}^{- 1}$ are momentum preserving. In addition, if we assume $w({\varphi },x)=\textrm{odd}({\varphi },x)$, then ${\varvec{\beta }}_{n-1},\breve{{\varvec{\beta }}}_{n-1}$ are $\textrm{odd}(\varphi ,x)$, the related maps ${{\mathcal {B}}}_{n-1}, {\mathcal B}_{n-1}^{- 1}$ are reversibility preserving, and $\textbf{b}_{n}$ is $\textrm{even}({\varphi },x)$.

#### Proof

We argue by induction on $n\in {\mathbb {N}}_0$. The claimed statements for $n=0$ follow directly by setting ${\mathtt m}_{0}:= 0$ and $\textbf{b}_{0}({\varphi },x):=\textbf{b}({\varphi },x)$, with $\textbf{b}({\varphi },x)$ defined in (5.2) with estimate (5.3).

We now assume that the claimed properties hold for some $n\geqslant 0$ and we prove them at the step $n+1$. We look for a diffeomorphism of the torus ${\mathbb {T}}^2 \rightarrow {\mathbb {T}}^2$, $x \mapsto x + {\varvec{\beta }}_{n}({\varphi },x)$, with inverse $y \mapsto y + \breve{{\varvec{\beta }}}_{n}({\varphi },x)$, such that, defining the operators

$$\begin{aligned} ({\mathcal {B}}_{n} u)({\varphi },x):= h ({\varphi }, x+ {\varvec{\beta }}_{n}({\varphi },x)) , \quad ({\mathcal {B}}_{n}^{-1} h ) ({\varphi },y):= h ({\varphi }, y + \breve{{\varvec{\beta }}}_{n}({\varphi },y)), \end{aligned}$$

the operator ${\mathcal {B}}_{n}^{-1} {\mathcal {T}}_{n} {\mathcal {B}}_{n}= \lambda \, {\mathcal {B}}_{n}^{-1} {\widetilde{{\mathcal {T}}}}_{n} {\mathcal {B}}_{n}$ has the desired properties. We compute

$$\begin{aligned} \begin{aligned} {\mathcal {B}}_{n}^{-1} {\widetilde{{\mathcal {T}}}}_{n} {\mathcal {B}}_{n}&= \omega \cdot \partial _{{\varphi }} + {\mathtt m}_{n} \cdot \nabla + \big \{ {\mathcal {B}}_{n}^{-1} \big ( \omega \cdot \partial _{{\varphi }} {\varvec{\beta }}_{n} + {\mathtt m}_{n} \cdot \nabla {\varvec{\beta }}_{n} + \textbf{b}_{n} + \textbf{b}_{n} \cdot \nabla {\varvec{\beta }}_{n} \big ) \big \} \cdot \nabla \\&= \omega \cdot \partial _{{\varphi }} + {\mathtt m}_{n} \cdot \nabla + \big \{ {\mathcal {B}}_{n}^{-1} \big ( \omega \cdot \partial _{{\varphi }} {\varvec{\beta }}_{n} + {\mathtt m}_{n} \cdot \nabla {\varvec{\beta }}_{n} + \Pi _{N_n} \textbf{b}_{n} \big ) \big \} \cdot \nabla + \textbf{b}_{n+1} \cdot \nabla , \end{aligned} \end{aligned}$$

where

5.16 $$\begin{aligned} \textbf{b}_{n+1}:= {\mathcal {B}}_{n}^{-1} \textbf{g}_{n} , \quad \textbf{g}_{n}:= \Pi _{N_n}^\perp \textbf{b}_{n} + \textbf{b}_{n} \cdot \nabla {\varvec{\beta }}_{n} , \end{aligned}$$

and the projectors $\Pi _{N_n}$, $\Pi _{N_n}^\perp$ are defined in (2.6). For any $\omega \in {\mathcal {O}}_{n+1}^\gamma$, we solve the homological equation

5.17 $$\begin{aligned} \omega \cdot \partial _{{\varphi }} {\varvec{\beta }}_{n} + {\mathtt m}_{n} \cdot \nabla {\varvec{\beta }}_{n} + \Pi _{N_n} \textbf{b}_{n} = {\langle {\textbf{b}_{n}}\rangle }_{{\varphi }} , \end{aligned}$$

where

$$\begin{aligned} {\langle {\textbf{b}_{n}}\rangle }_{{\varphi }}:= \frac{1}{(2\pi )^{\nu }} \int _{{\mathbb {T}}^{\nu }} \textbf{b}_{n}({\varphi },x) \,\textrm{d}{{\varphi }}. \end{aligned}$$

Note that, since $\textbf{b}_{n}$ is a quasi-periodic traveling wave, we have that ${\langle {\textbf{b}_{n}}\rangle }_{{\varphi }}={\langle {\textbf{b}_{n}}\rangle }_{{\varphi },x}\in {\mathbb {R}}^2$ is constant, since

$$\begin{aligned} \frac{1}{(2\pi )^\nu } \int _{{\mathbb {T}}^\nu }\textbf{b}_{n}({\varphi },x)\,\textrm{d}{{\varphi }}= \sum _{j\in {\mathbb {Z}}^2 \atop \pi ^\top (0)+j=0} {\widehat{\textbf{b}_{n}}}(0,j) e^{\textrm{i}\,jx} ={\widehat{\textbf{b}_{n}}}(0,0) ={\langle {\textbf{b}_{n}}\rangle }_{{\varphi },x}. \end{aligned}$$

Explicitly, the solution of the equation (5.17) is given by

5.18 $$\begin{aligned} \begin{aligned} {\varvec{\beta }}_{n}({\varphi },x)&:= -( \omega \cdot \partial _{{\varphi }} + {\mathtt m}_{n} \cdot \nabla )^{-1} \big [ \Pi _{N_n}\textbf{b}_{n} - {\langle {\textbf{b}_{n}}\rangle }_{{\varphi }} \big ] \\&:= - \sum _{(\ell , j) \in {\mathbb {Z}}^{\nu +2} \setminus \{0\}, \atop |\ell |\leqslant N_n, \, \pi ^\top (\ell ) + j = 0} \frac{1}{\textrm{i}(\omega \cdot \ell + {\mathtt m}_{n}\cdot j)} {\widehat{\textbf{b}}}_{n}(\ell ,j) e^{\textrm{i}(\ell \cdot {\varphi }+ j\cdot x)} . \end{aligned} \end{aligned}$$

We define

5.19 $$\begin{aligned} \begin{aligned} {\mathcal {T}}_{n+1}&:= \lambda \, {\widetilde{{\mathcal {T}}}}_{n+1}, \quad {\widetilde{{\mathcal {T}}}}_{n+1}:= \omega \cdot \partial _{{\varphi }} + {\mathtt m}_{n+1} \cdot \nabla + \textbf{b}_{n+1} \cdot \nabla , \\ {\mathtt m}_{n+1}&:= {\mathtt m}_{n} + {\langle {\textbf{b}_{n}}\rangle }_{{\varphi }} . \end{aligned} \end{aligned}$$

Therefore, for any $\omega \in {\mathcal {O}}_{n+1}^\gamma$, we have that ${\widetilde{{\mathcal {T}}}}_{n+1}= {\mathcal {B}}_{n}^{-1} {\widetilde{{\mathcal {T}}}}_{n} {\mathcal {B}}_{n}$, so that (5.15) holds at the step $n+1$. By (5.18) and Lemma 2.4, we have, for any $s\geqslant 0$,

5.20 $$\begin{aligned} \begin{aligned} \Vert {\varvec{\beta }}_{n} \Vert _{s}^{\textrm{Lip}(\gamma )}&\lesssim _{s} \Vert \Pi _{N_n} \textbf{b}_{n} \Vert _{s+2\tau +1}^{\textrm{Lip}(\gamma )} \lesssim _{s} N_n^{2\tau +1} \gamma ^{-1} \Vert \textbf{b}_{n}\Vert _{s}^{\textrm{Lip}(\gamma )} , \\ \Vert \nabla {\varvec{\beta }}_{n} \Vert _{s}^{\textrm{Lip}(\gamma )}&\lesssim _{s} \Vert \Pi _{N_n} \textbf{b}_{n} \Vert _{s+2\tau +2}^{\textrm{Lip}(\gamma )} \lesssim _{s} N_n^{2\tau +2} \gamma ^{-1} \Vert \textbf{b}_{n}\Vert _{s}^{\textrm{Lip}(\gamma )} . \end{aligned} \end{aligned}$$

The latter estimate, together with the induction estimate (5.12) on $\textbf{b}_{n}$, imply, for any $s \in [s_0,S-\sigma ]$,

5.21 $$\begin{aligned} \begin{aligned} \Vert {\varvec{\beta }}_{n+1} \Vert _{s}^{\textrm{Lip}(\gamma )} , \Vert \breve{{\varvec{\beta }}}_{n+1} \Vert _{s}^{\textrm{Lip}(\gamma )}&\lesssim _{s} N_n^{2\tau +1} N_{n-1}^{-{\mathfrak {a}}} \varepsilon \gamma ^{-1} \Vert w \Vert _{s+\sigma }^{\textrm{Lip}(\gamma )} , \\ \Vert {\varvec{\beta }}_{n+1} \Vert _{s+{\mathfrak {b}}}^{\textrm{Lip}(\gamma )} , \Vert \breve{{\varvec{\beta }}}_{n+1} \Vert _{s+{\mathfrak {b}}}^{\textrm{Lip}(\gamma )}&\lesssim _{s} N_n^{2\tau +1} N_{n-1} \varepsilon \gamma ^{-1} \Vert w \Vert _{s+\sigma }^{\textrm{Lip}(\gamma )} , \end{aligned} \end{aligned}$$

which are the estimates (5.14) at the step $n+1$. Note that, by the definition of the constant ${\mathfrak {a}}$ in (5.9) and the ansatz (4.2), from (5.21) we deduce, with $s=s_0$,

$$\begin{aligned} \Vert {\varvec{\beta }}_{n+1}\Vert _{s_0}^{\textrm{Lip}(\gamma )} , \Vert \breve{{\varvec{\beta }}}_{n+1}\Vert _{s_0}^{\textrm{Lip}(\gamma )} \lesssim N_0^{2\tau +1} \varepsilon \gamma ^{-1}. \end{aligned}$$

Together with the smallness condition (5.4), Lemma 2.14 implies that, for any $s_0 \leqslant s \leqslant S-\sigma +{\mathfrak {b}}$,

5.22 $$\begin{aligned} \Vert {\mathcal {B}}_{n}^{\pm 1} h\Vert _{s}^{\textrm{Lip}(\gamma )} \lesssim _{s} \Vert h \Vert _{s}^{\textrm{Lip}(\gamma )} + N_n^{2\tau +1} \gamma ^{-1} \Vert \textbf{b}_{n} \Vert _{s}^{\textrm{Lip}(\gamma )} \Vert h \Vert _{s_0}^{\textrm{Lip}(\gamma )}. \end{aligned}$$

Now we estimate $\textbf{b}_{n+1}$ in (5.16). First, we estimate $\textbf{g}_{n}$. By (5.9), (5.12), the ansatz (4.2) and the smallness condition (5.4), we have

5.23 $$\begin{aligned} N_n^{2\tau +2} \gamma ^{-1} \Vert \textbf{b}_{n} \Vert _{s_0}^{\textrm{Lip}(\gamma )} \leqslant 1. \end{aligned}$$

By Lemma 2.4, Lemma 2.2 and estimates (5.20), (5.23), we have, for any $s \in [s_0,S-\sigma ]$,

5.24 $$\begin{aligned} \begin{aligned}&\Vert \textbf{g}_{n} \Vert _{s_0}^{\textrm{Lip}(\gamma )} \lesssim \Vert \textbf{b}_{n}\Vert _{s_0}^{\textrm{Lip}(\gamma )} , \\&\quad \Vert \textbf{g}_{n} \Vert _{s}^{\textrm{Lip}(\gamma )} \lesssim _{s} N_n^{-{\mathfrak {b}}} \Vert \textbf{b}_{n} \Vert _{s+{\mathfrak {b}}}^{\textrm{Lip}(\gamma )} + N_{n-1}^{2\tau +2} \gamma ^{-1} \Vert \textbf{b}_{n} \Vert _{s}^{\textrm{Lip}(\gamma )} \Vert \textbf{b}_{n} \Vert _{s_0}^{\textrm{Lip}(\gamma )} , \\&\quad \Vert \textbf{g}_{n} \Vert _{s+{\mathfrak {b}}}^{\textrm{Lip}(\gamma )} \lesssim _{s} \Vert \textbf{b}_{n} \Vert _{s+{\mathfrak {b}}}^{\textrm{Lip}(\gamma )} (1 + N_{n}^{2\tau +2} \gamma ^{-1} \Vert \textbf{b}_{n} \Vert _{s_0}^{\textrm{Lip}(\gamma )}) \lesssim _{s} \Vert \textbf{b}_{n} \Vert _{s+{\mathfrak {b}}}^{\textrm{Lip}(\gamma )} . \end{aligned} \end{aligned}$$

Therefore, estimates (5.22)-(5.24) imply that, for any $s \in [s_0,S-\sigma ]$,

$$\begin{aligned} \begin{aligned}&\Vert \textbf{b}_{n+1} \Vert _{s}^{\textrm{Lip}(\gamma )} \lesssim _{s} N_n^{-{\mathfrak {b}}} \Vert \textbf{b}_{n} \Vert _{s+{\mathfrak {b}}}^{\textrm{Lip}(\gamma )} + N_n^{2\tau +2} \gamma ^{-1} \Vert \textbf{b}_{n} \Vert _{s}^{\textrm{Lip}(\gamma )} \Vert \textbf{b}_{n} \Vert _{s_0}^{\textrm{Lip}(\gamma )} , \\&\quad \Vert \textbf{b}_{n} \Vert _{s+{\mathfrak {b}}}^{\textrm{Lip}(\gamma )} \lesssim _{s} \Vert \textbf{b}_{n} \Vert _{s+{\mathfrak {b}}}^{\textrm{Lip}(\gamma )} . \end{aligned} \end{aligned}$$

Using the definition of the constants ${\mathfrak {a}}$, ${\mathfrak {b}}$ in (5.9) and the induction assuptions on the estimates (5.12), by a standard argument we deduce the estimates (5.12) at the step $n+1$. The estimates (5.13) follow by the definition of ${\mathtt m}_{n+1}$ in (5.19), the induction assumption on (5.12) and by a telescopic argument.

Finally, by (5.18), since $\textbf{b}_{n}({\varphi },x)$ is a quasi-periodic traveling wave by induction assumption, we have that ${\varvec{\beta }}_{n}({\varphi },x)$, $\breve{{\varvec{\beta }}}_{n}({\varphi },y)$ are quasi-periodic traveling waves by Lemma 2.15, which also imply that ${\mathcal {B}}_{n}^{\pm 1}$ are momentum preserving operators. Recalling (5.16), we deduce that also $\textbf{b}_{n+1}({\varphi },y)$ is a quasi-periodic traveling wave.

Finally if *w* is $\textrm{odd}({\varphi }, x)$, by (5.18), since $\textbf{b}_{n}({\varphi },x)$ is $\textrm{even}({\varphi },x)$, we have that ${\varvec{\beta }}_{n}({\varphi },x)$ and $\breve{{\varvec{\beta }}}_{n}({\varphi },y)$ are $\textrm{odd}({\varphi },y)$ and hence ${\mathcal {B}}_{n}^{\pm 1}$ are reversibility preseving operators. Recalling (5.16), we deduce that also $\textbf{b}_{n+1}({\varphi },y)$ is $\textrm{even}({\varphi },y)$. This concludes the proof. $\square$

We then define

5.25 $$\begin{aligned} {\widetilde{{\mathcal {B}}}}_{n}:= {\mathcal {B}}_{0} \circ {\mathcal {B}}_{1} \circ ... \circ {\mathcal {B}}_{n} , \quad \text {with inverse} \quad {\widetilde{{\mathcal {B}}}}_{n}^{-1} = {\mathcal {B}}_{n}^{-1} \circ ... \circ {\mathcal {B}}_{1}^{-1} \circ {\mathcal {B}}_{0}^{-1} . \end{aligned}$$

#### Lemma 5.3

Let $\gamma \in (0,1)$ and $\tau >\nu -1$. There exists $\sigma := \sigma (\tau , \nu ) > 0$ large enough such that, for any $S > s_0 + \sigma$, there exist $\delta := \delta (S, \tau , \nu ) \in (0, 1)$ small enough, $N_0=N_0(S,\tau ,\nu )>0$ large enough and $\tau _{1}=\tau _{1}(\tau ,\nu )>0$ such that, if (4.2), (5.3) and (5.4) hold for $\sigma _{0}\geqslant s_0 +\sigma$, the following hold:

(*i*) For any $n \in {\mathbb {N}}_0$, we have

$$\begin{aligned} ({\widetilde{{\mathcal {B}}}}_{n} u)({\varphi },x):= h ({\varphi }, x+ {\varvec{\alpha }}_{n}({\varphi },x)) , \quad ({\widetilde{{\mathcal {B}}}}_{n}^{-1} h ) ({\varphi },y):= h ({\varphi }, y + \breve{{\varvec{\alpha }}}_{n}({\varphi },y)), \end{aligned}$$

for some functions ${\varvec{\alpha }}_{n}({\varphi },x)$, $\breve{{\varvec{\alpha }}}_{n}({\varphi },y)$ such that, for any $s\in [s_0,S-\sigma ]$,

$$\begin{aligned} \begin{aligned}&\Vert {\varvec{\alpha }}_{0}\Vert _{s}^{\textrm{Lip}(\gamma )}, \ \Vert \breve{{\varvec{\alpha }}}_{0}\Vert _{s}^{\textrm{Lip}(\gamma )} \lesssim _{s} N_0^{2\tau +1} \varepsilon \gamma ^{-1} \Vert w \Vert _{s+\sigma }^{\textrm{Lip}(\gamma )} , \\&\quad \Vert {\varvec{\alpha }}_{n} - {\varvec{\alpha }}_{n-1} \Vert _{s}^{\textrm{Lip}(\gamma )}, \ \Vert \breve{{\varvec{\alpha }}}_{n}- \breve{{\varvec{\alpha }}}_{n-1} \Vert _{s}^{\textrm{Lip}(\gamma )} \lesssim _{s} N_n^{2\tau +1} N_{n-1}^{-{\mathfrak {a}}} \varepsilon \gamma ^{-1} \Vert w \Vert _{s+\sigma }^{\textrm{Lip}(\gamma )} , \quad n\geqslant 1. \end{aligned} \end{aligned}$$

As a consequence, for any $s \in [s_0,S-\sigma ]$,

5.26 $$\begin{aligned} \Vert {\varvec{\alpha }}_{n}\Vert _{s}^{\textrm{Lip}(\gamma )} \lesssim _{s} N_0^{2\tau +1} \varepsilon \gamma ^{-1} \Vert w \Vert _{s+\sigma }^{\textrm{Lip}(\gamma )} . \end{aligned}$$

Furthermore, ${\varvec{\alpha }}_{n}$, $\breve{{\varvec{\alpha }}}_{n}$ are both quasi-periodic traveling waves. In addition, if we assume $\textbf{b}_{0}({\varphi },x)=\textrm{even}({\varphi },x)$, we have that ${\varvec{\alpha }}_{n}({\varphi },x)$ is $\textrm{odd}({\varphi },x)$, $\breve{{\varvec{\alpha }}}_{n}({\varphi },y)$ is $\textrm{odd}({\varphi },y)$;

(*ii*) For any $s \in [s_0,S-\sigma ]$, the sequence $({\varvec{\alpha }}_{n}({\varphi },x))_{n\in {\mathbb {N}}_0}$ (resp. $(\breve{{\varvec{\alpha }}}_{n}({\varphi },y))_{n\in {\mathbb {N}}_0}$) is a Cauchy sequence with respect to the norm $\Vert \,\cdot \,\Vert _{s}^{\textrm{Lip}(\gamma )}$ and it converges to some limit ${\varvec{\beta }}({\varphi },x)$ (resp. $\breve{{\varvec{\beta }}}({\varphi },y)$), with estimates, for any $n\in {\mathbb {N}}_0$,

$$\begin{aligned} \begin{aligned}&\Vert {\varvec{\beta }}- {\varvec{\alpha }}_{n} \Vert _{s}^{\textrm{Lip}(\gamma )} , \ \Vert \breve{{\varvec{\beta }}} - \breve{{\varvec{\alpha }}}_{n}\Vert _{s}^{\textrm{Lip}(\gamma )} \lesssim _{s} N_{n+1}^{2\tau +1} N_{n}^{1-{\mathfrak {a}}} \varepsilon \gamma ^{-1} \Vert w \Vert _{s+\sigma }^{\textrm{Lip}(\gamma )} , \\&\quad \Vert {\varvec{\beta }}\Vert _{s}^{\textrm{Lip}(\gamma )}, \ \Vert \breve{{\varvec{\beta }}} \Vert _{s}^{\textrm{Lip}(\gamma )}\lesssim _{s} N_0^{2\tau +1} \varepsilon \gamma ^{-1} \Vert w \Vert _{s+\sigma } . \end{aligned} \end{aligned}$$

Furthermore, ${\varvec{\beta }}$, $\breve{{\varvec{\beta }}}$ are both quasi-periodic traveling waves. In addition, if we assume $\textbf{b}_{0}({\varphi },x)=\textrm{even}({\varphi },x)$, we have that ${\varvec{\beta }}({\varphi },x)$ is $\textrm{odd}({\varphi },x)$, $\breve{{\varvec{\beta }}}({\varphi },y)$ is $\textrm{odd}({\varphi },y)$;

(*iii*) Define the operators

$$\begin{aligned} ({\mathcal {B}}u)({\varphi },x):= h ({\varphi }, x+ {\varvec{\beta }}({\varphi },x)) , \quad ({\mathcal {B}}^{-1} h ) ({\varphi },y):= h ({\varphi }, y + \breve{{\varvec{\beta }}}({\varphi },y)). \end{aligned}$$

Then, for any $s \in [s_0,S-\sigma ]$, the sequences $({\widetilde{{\mathcal {B}}}}_{n}^{\pm 1})_{n\in {\mathbb {N}}_0}$ converge strongly in $H^s({\mathbb {T}}^{\nu +2})$ to ${\mathcal {B}}^{\pm 1}$, namely $\lim _{n\rightarrow + \infty }\Vert ({\mathcal {B}}^{\pm 1}- {\widetilde{{\mathcal {B}}}}_{n}^{\pm 1})h \Vert _{s} =0$ for any $h \in H^s({\mathbb {T}}^{\nu +2})$. Furthermore, ${\mathcal {B}}^{\pm 1}$ are momentum preserving. In addition, if we assume $w({\varphi },x)=\textrm{odd}({\varphi },x)$, they are reversibility preserving.

#### Proof

See the proof of Lemma 4.4 in [7]. The property of the quasi-periodic traveling wave functions and momentum preserving operators follow from Lemma 5.2, since both the properties are closed under limit. $\square$

#### Lemma 5.4

The sequence $({\mathtt m}_{n})_{n\in {\mathbb {N}}_0}$ satisfies the bound

5.27 $$\begin{aligned} \sup _{\omega \in {\mathcal {O}}_{n}^\gamma } |{\mathtt m}_{n}(\omega )| \lesssim \varepsilon N_{n-1}^{-{\mathfrak {a}}} . \end{aligned}$$

Furthermore, we have that ${\mathtt D}{\mathtt C}(2\gamma ,\tau )\cap \Lambda _{o} \subseteq \cap _{n \geqslant 0} {\mathcal {O}}_{n}^\gamma$ (recalling (3.5), (4.2), (3.6), (5.11)).

#### Proof

We start with the proof of (5.27). By (5.25), using (5.10) and (5.15), we have

5.28 $$\begin{aligned} \omega \cdot \partial _{{\varphi }} + {\mathtt m}_{n} \cdot \nabla + \textbf{b}_{n} \cdot \nabla = {\widetilde{{\mathcal {T}}}}_{n} = {\widetilde{{\mathcal {B}}}}_{n-1}^{-1} {\widetilde{{\mathcal {T}}}}_{0} {\widetilde{{\mathcal {B}}}}_{n-1} \quad \forall \,\omega \in {\mathcal {O}}_{n}^\gamma , \end{aligned}$$

and, by Lemma (5.3)-(*i*), we compute explicitly

5.29 $$\begin{aligned} {\widetilde{{\mathcal {B}}}}_{n-1}^{-1} {\widetilde{{\mathcal {T}}}}_{0} {\widetilde{{\mathcal {B}}}}_{n-1} = \omega \cdot \partial _{{\varphi }} + {\widetilde{{\mathcal {B}}}}_{n-1}^{-1} \big ( \omega \cdot \partial _{{\varphi }} {\varvec{\alpha }}_{n-1} + \textbf{b}+ \textbf{b}\cdot \nabla {\varvec{\alpha }}_{n-1} \big ) \cdot \nabla . \end{aligned}$$

Since, clearly, ${\widetilde{{\mathcal {B}}}}_{n-1} [c]= c$ for any constant $c\in {\mathbb {R}}$, we have, by (5.28), (5.29),

5.30 $$\begin{aligned} {\mathtt m}_{n} + {\widetilde{{\mathcal {B}}}}_{n-1} \textbf{b}_{n} = \omega \cdot \partial _{{\varphi }} {\varvec{\alpha }}_{n-1}+ \textbf{b}+ \textbf{b}\cdot {\varvec{\alpha }}_{n-1} . \end{aligned}$$

We note that, by (4.8), (5.2),

5.31 $$\begin{aligned} \begin{aligned}&\int _{{\mathbb {T}}^{\nu +2}} \omega \cdot \partial _{{\varphi }}{\varvec{\alpha }}_{n-1} \,\textrm{d}{{\varphi }}\,\textrm{d}{x} = 0 , \quad \int _{{\mathbb {T}}^{\nu +2}} \textbf{b}({\varphi },x) \,\textrm{d}{{\varphi }}\,\textrm{d}{x} = 0, \\&\quad \int _{{\mathbb {T}}^{\nu +2}} \textbf{b}\cdot \nabla {\varvec{\alpha }}_{n-1} \,\textrm{d}{{\varphi }}\,\textrm{d}{x} = - \int _{{\mathbb {T}}^{\nu +2}} \textrm{div}(\textbf{b}) {\varvec{\alpha }}_{n-1} \,\textrm{d}{{\varphi }}\,\textrm{d}{x} = 0. \end{aligned} \end{aligned}$$

Taking the space-time average of the equation (5.30) and using (5.31), we deduce that

$$\begin{aligned} {\mathtt m}_{n} = - \int _{{\mathbb {T}}^{\nu +2}} {\widetilde{{\mathcal {B}}}}_{n-1} \textbf{b}_{n} \,\textrm{d}{{\varphi }}\,\textrm{d}{x} \quad \forall \,\omega \in {\mathcal {O}}_{n}^\gamma . \end{aligned}$$

The claimed estimate (5.27) follows by Lemmata 2.2, 2.14 and estimates (5.12), (5.26), (4.2) and (5.4).

We now prove the claim on the inclusion. We argue by induction, that is, we show that ${\mathtt D}{\mathtt C}(2\gamma ,\tau ) \cap \Lambda _{o} \subseteq {\mathcal {O}}_{n}^\gamma$ for any $n\in {\mathbb {N}}_0$. For $n=0$, we have that ${\mathcal {O}}_{n}^\gamma = \Lambda _{o}$, so the claim is trivially satisfied. Now we assume that ${\mathtt D}{\mathtt C}(2\gamma ,\tau ) \cap \Lambda _{o} \subseteq {\mathcal {O}}_{n}^\gamma$ for some $n\geqslant 0$ and we want to prove the inclusion with $n+1$. Let $\omega \in {\mathtt D}{\mathtt C}(2\gamma ,\tau )\cap \Lambda _{o}$. By induction hypothesis, $\omega \in {\mathcal {O}}_{n}^\gamma$. Therefore, by (5.27), we have $|{\mathtt m}_{n}| \lesssim \varepsilon N_{n-1}^{-{\mathfrak {a}}}$. Hence, for any $(\ell ,j)\in {\mathbb {Z}}^{\nu +2}$, with $0<|\ell | \leqslant N_{n}$ and $\pi ^\top (\ell )+j=0$ (which implies $|j|\lesssim N_{n}$), we have that

$$\begin{aligned} \begin{aligned} |\omega \cdot \ell +{\mathtt m}_{n}(\omega )\cdot j|&\geqslant |\omega \cdot \ell | - |{\mathtt m}_{n}(\omega )\cdot j| \\&\geqslant \frac{2\gamma }{{\langle {\ell }\rangle }^{\tau }} - \varepsilon C N_{n} N_{n-1}^{-{\mathfrak {a}}} \geqslant \frac{\gamma }{{\langle {\ell }\rangle }^\tau } , \end{aligned} \end{aligned}$$

for some constant $C>0$, provided that $C N_{n}^{1+\tau } N_{n-1}^{-{\mathfrak {a}}} \varepsilon \gamma ^{-1}\leqslant 1$. This holds for any $n\geqslant 0$, provided $C N_0^{1+\tau }\varepsilon \gamma ^{-1} \leqslant 1$, which is satisfied by the smallness condition (5.4) and by (5.9). Thus, by the definition of ${\mathcal {O}}_{n+1}^\gamma$ (see (5.11) with $n+1$), we have $\omega \in {\mathcal {O}}_{n+1}^\gamma$. The proof is concluded. $\square$

#### Proof of Proposition 5.1

For any $\omega \in {\mathtt D}{\mathtt C}(2\gamma ,\tau )\cap \Lambda _{o}$, by Lemma 5.4, ${\mathtt m}_{n}\rightarrow 0$ as $n\rightarrow \infty$. By (5.26), (5.12) and Lemma 2.2, we have $\Vert {\widetilde{{\mathcal {B}}}}_{n-1} \textbf{b}_{n} \Vert _{s_0} \lesssim \Vert \textbf{b}_{n}\Vert \rightarrow 0$ as $n\rightarrow \infty$. Moreover,

$$\begin{aligned} \Vert \partial _{{\varphi }} {\varvec{\alpha }}_{n-1} - \partial _{{\varphi }}{\varvec{\beta }}\Vert _{s_0}, \Vert \nabla {\varvec{\alpha }}_{n-1}-\nabla {\varvec{\beta }}\Vert _{s_0} \leqslant \Vert {\varvec{\alpha }}_{n-1}-{\varvec{\beta }}\Vert _{s_0+1} \rightarrow 0 \quad \text {as} \ \ n\rightarrow \infty . \end{aligned}$$

Hence, passing to the limit in the norm $\Vert \,\cdot \,\Vert _{s_0}$ in identity (5.30), we obtain the identity

5.32 $$\begin{aligned} \omega \cdot \partial _{{\varphi }} {\varvec{\beta }}+ \textbf{b}({\varphi },x) + \textbf{b}({\varphi },x) \cdot {\varvec{\beta }}=0 \end{aligned}$$

in $H^{s_0}({\mathbb {T}}^{\nu +1})$ and therefore pointwise for all $({\varphi },x) \in {\mathbb {T}}^{\nu +2}$, for any $\omega \in {\mathtt D}{\mathtt C}(2\gamma ,\tau )\cap \Lambda _{o}$. As a consequence,

$$\begin{aligned} {\mathcal {B}}^{-1} {\widetilde{{\mathcal {T}}}}{\mathcal {B}}= \omega \cdot \partial _{{\varphi }} + \big \{ {\mathcal {B}}^{-1} \big ( \omega \cdot \partial _{{\varphi }}{\varvec{\beta }}+ \textbf{b}+ \textbf{b}\cdot \nabla {\varvec{\beta }}\big ) \big \} \cdot \nabla = \omega \cdot \partial _{{\varphi }} \end{aligned}$$

for all $\omega \in {\mathtt D}{\mathtt C}(2\gamma ,\tau )\cap \Lambda _{o}$, which shows (5.6). Estimates (5.5), (5.7) follow by Lemma 5.3-(*ii*) and Lemma 2.2.

It remains to prove the estimates (5.8). Let $\textbf{b}_{i}:= \textbf{b}_{i}(w_i)$, $i=1,2$ satisfy (4.8) and assume that, for $s_1 > s_0$, $w_1,w_2$ satisfy (4.2), with $\sigma _0 \geqslant s_1 + \sigma$. Let ${\varvec{\beta }}_{i}$ and ${\mathcal {B}}_{i}$, $i=1,2$, be the corresponding functions and operators given by Lemma (5.3)-(*ii*),(*iii*). Then, by (5.32) and (5.6), for $i=1,2$, we have, for any $\omega \in {\mathtt D}{\mathtt C}(2\gamma ,\tau )\cap \Lambda _{o}(w_1)\cap \Lambda _{o}(w_2)$,

5.33 $$\begin{aligned} {\mathcal {B}}_{i}^{-1} (\omega \cdot \partial _{{\varphi }} + \textbf{b}_{i} \cdot \nabla ) {\mathcal {B}}_{i} = \omega \cdot \partial _{{\varphi }}, \quad (\omega \cdot \partial _{{\varphi }} + \textbf{b}_{i}\cdot \nabla )[{\varvec{\beta }}_{i}] + \textbf{b}_{i} = 0. \end{aligned}$$

Hence, with $\Delta _{12}{\varvec{\beta }}:= {\varvec{\beta }}_{1}(w_1)-{\varvec{\beta }}_{2}(w_2)$ and $\Delta _{12}\textbf{b}:= \textbf{b}_{1}(w_1)-\textbf{b}_{2}(w_2)$,

$$\begin{aligned} (\omega \cdot \partial _{{\varphi }} + \textbf{b}_{1}(w_1) \cdot \nabla )[\Delta _{12}{\varvec{\beta }}] + \mathfrak {g}= 0, \quad \mathfrak {g}:= \Delta _{12}\textbf{b}\cdot \nabla \textbf{b}_{2}(w_2) + \Delta _{12}\textbf{b}. \end{aligned}$$

By (5.33), we have $\omega \cdot \partial _{{\varphi }} + \textbf{b}_{1}(w_1)\cdot \nabla = {\mathcal {B}}_{1}^{-1} \omega \cdot \partial _{{\varphi }} {\mathcal {B}}_{1}$, and therefore

$$\begin{aligned} \omega \cdot \partial _{{\varphi }} {\mathcal {B}}_{1}^{-1}\Delta _{12}{\varvec{\beta }}+ {\mathcal {B}}_{1}^{-1} \mathfrak {g}= 0. \end{aligned}$$

Since ${\langle {{\mathcal {B}}_{1}^{-1} \mathfrak {g}}\rangle }_{{\varphi },x} = - {\langle {\omega \cdot \partial _{{\varphi }} {\mathcal {B}}_{1}^{-1}\Delta _{12}{\varvec{\beta }}}\rangle }_{{\varphi },x}=0$ for any $\omega \in {\mathtt D}{\mathtt C}(2\gamma ,\tau )\cap \Lambda _{o}(w_1)\cap \Lambda _{o}(w_2)$ and $\Delta _{12}{\varvec{\beta }}$, ${\mathcal {B}}_{1}^{-1}\Delta _{12}{\varvec{\beta }}= \textrm{odd}({\varphi },x)$ (by Lemma 5.3-(*ii*),(*iii*)), one has

$$\begin{aligned} \Delta _{12}{\varvec{\beta }}= - {\mathcal {B}}_{1} (\omega \cdot \partial _{{\varphi }})^{-1} {\mathcal {B}}_{1}^{-1} \mathfrak {g}, \quad {\forall \,\omega \in {\mathtt D}{\mathtt C}(2\gamma ,\tau )\cap \Lambda _{o}(w_1)\cap \Lambda _{o}(w_2)} . \end{aligned}$$

Then, having $\Vert w_{i}\Vert _{s_1+\sigma }\leqslant 1$, by (5.7), (5.5) and Lemma 2.2, recalling also that $\textbf{b}_{i} = \varepsilon {\mathfrak {B}}[w_i]$ by (5.2), (4.4), $i=1,2$, we get the estimate in (5.8) for $\Delta _{12}{\varvec{\beta }}$. The corresponding estimate for $\Delta _{12}\breve{{\varvec{\beta }}}$ can be proved by using that $\breve{{\varvec{\beta }}}_{i}=-{\mathcal {B}}_{i}^{-1}{\varvec{\beta }}_{i}$ and the mean value theorem. Finally, the estimates for $\Delta _{12}{\mathcal {B}}^{\pm 1}$ follow by the estimates for $\Delta _{12}{\varvec{\beta }}$, $\Delta _{12}\breve{{\varvec{\beta }}}$, the mean value theorem and Lemma 2.2. The proof is concluded. $\square$

We now compute the complete conjugation of the operator ${\mathcal {L}}: H_0^{s+1}({\mathbb {T}}^{\nu +2})\rightarrow H_0^s({\mathbb {T}}^{\nu +2})$ in (4.3) by means of the transformation in (5.1), with ${\varvec{\beta }}({\varphi },x)$ as in Proposition 5.1.

#### Proposition 5.5

Let $S>s_0+\sigma _{1}$, for some $\sigma _{1}=\sigma _{1}(\tau ,\nu ) \gg \sigma$ (where $\sigma$ is provided by Proposition 5.1). There exists $\delta (S,\tau ,\nu )\in (0,1)$ small enough such that, if (4.2) and (5.4) are fulfilled, the following holds:

(*i*) Let ${\mathcal {B}}_{\perp }:= \Pi _0^{\perp } {\mathcal {B}}\Pi _0^{\perp }$, where $\Pi _0^{\perp }$ is defined in (2.17) and the invertible map ${\mathcal {B}}$, with inverse ${\mathcal {B}}^{-1}$, is constructed in Proposition 5.1. Then, for any $s_{0} \leqslant s\leqslant S-\sigma$, with $\sigma$ as in Proposition (5.1), the operator ${\mathcal {B}}_{\perp }: H^s_0({\mathbb {T}}^{\nu + 2}) \rightarrow H^s_0({\mathbb {T}}^{\nu + 2})$ is invertible with bounded inverse given by ${\mathcal {B}}_{\perp }^{-1}:= \Pi _0^\perp {\mathcal {B}}^{-1}\Pi _0^\perp : H_0^{s}({\mathbb {T}}^{\nu +2})\rightarrow H_{0}^{s}({\mathbb {T}}^{\nu +2})$;

(*ii*) For any $\omega \in {\mathtt D}{\mathtt C}(2\gamma ,\tau )\cap \Lambda _{o}$, as in (3.5), one has

5.34 $$\begin{aligned} {\mathcal {L}}_{1}: ={\mathcal {B}}_{\perp }^{-1} {\mathcal {L}}\, {\mathcal {B}}_{\perp } = \lambda \, \omega \cdot \partial _{{\varphi }} + \beta \,{\mathtt L}+ {\mathcal {E}}_{1}, \end{aligned}$$

where, for any $s_0 \leqslant s \leqslant S - \sigma _1$,

the operator ${\mathcal {E}}_{1}\in {{\mathcal {O}}}{{\mathcal {P}}}{{\mathcal {M}}}_{s}^{-1}$ satisfies the estimate

5.35 $$\begin{aligned} | {\mathcal {E}}_{1} |_{-1,s}^{\textrm{Lip}(\gamma )} \lesssim _{s} \lambda ^{\theta } \Vert w \Vert _{s + \sigma _1}^{\textrm{Lip}(\gamma )}. \end{aligned}$$

Let $s_1 \geqslant s_0$ and assume that $w_1, w_2$ satisfy (4.2) with $\sigma _{0} \geqslant s_1 + \sigma _{1}$. Then for any $\omega \in {\mathtt D}{\mathtt C}(2\gamma ,\tau )\cap \Lambda _{o}(w_1)\cap \Lambda _{o}(w_2)$ one has

5.36 $$\begin{aligned} \begin{aligned}&| \Delta _{12} {\mathcal {E}}_{1} |_{-1,s_1} \lesssim _{s_1} \lambda ^{\theta } \Vert w_1 -w_2 \Vert _{s_1 +\sigma _{1}}. \end{aligned} \end{aligned}$$

Furthermore, the operators ${\mathcal {L}}_{1}$ and ${\mathcal {E}}_{1}$ are momentum preserving and, if we assume that $w({\varphi },x)= \textrm{odd}({\varphi },x)$, they are reversibility preserving.

#### Proof

Item (*i*) is proved in Lemma 4.2 in [27]. We now prove item (*ii*). By (4.3), (5.2), we write ${\mathcal {L}}= {\mathcal {T}}+ \beta \,{\mathtt L}+ \lambda ^{\theta } {\mathcal {E}}_{0}$. We have to compute

$$\begin{aligned} \begin{aligned} {\mathcal {B}}_{\perp }^{-1} {\mathcal {L}}\,{\mathcal {B}}_{\perp }&= {\mathcal {B}}_{\perp }^{-1} {\mathcal {T}}{\mathcal {B}}_{\perp } + \beta \, {\mathcal {B}}_{\perp }^{-1} {\mathtt L}\, {\mathcal {B}}_{\perp } +\lambda ^{\theta } {\mathcal {B}}_{\perp }^{-1} {\mathcal {E}}_{0}\, {\mathcal {B}}_{\perp } . \end{aligned} \end{aligned}$$

By Proposition 5.1, by item (*i*) and by (4.10), we have

$$\begin{aligned} {\mathcal {B}}_{\perp }^{-1} {\mathcal {T}}{\mathcal {B}}_{\perp } = \Pi _0^\perp {\mathcal {B}}^{-1} {\mathcal {T}}{\mathcal {B}}\Pi _0^{\perp } = \Pi _0^{\perp } \,\lambda \,\omega \cdot \partial _{{\varphi }} \Pi _0^{\perp } = \lambda \,\omega \cdot \partial _{{\varphi }} \textrm{Id}_0 \end{aligned}$$

where $\textrm{Id}_0$ is the identity on $L_0^2({\mathbb {T}}^{\nu +2})$. To compute the other terms, the main tool is Lemma 2.16-(*ii*), which provides the structure for the conjugation of the operator $(-\Delta )^{-1}$ as in (2.19). Recalling by (1.5) that ${\mathtt L}=-(-\Delta )^{-1} \partial _{x_1}$, which is invariant on the functions with zero average, we compute

5.37 $$\begin{aligned} \begin{aligned} {\mathcal {B}}_{\perp }^{-1} {\mathtt L}\, {\mathcal {B}}_{\perp }&= - \Pi _0^{\perp }\big ( {\mathcal {B}}^{-1} (-\Delta )^{-1} {\mathcal {B}}\big ) \Pi _0^{\perp } \big ( {\mathcal {B}}^{-1} \partial _{x_1} {\mathcal {B}}\big )\Pi _0^\perp \\&= - \big ( (-\Delta )^{-1} + {\mathcal {P}}_{-2} \big ) \Pi _0^\bot \{ {\mathcal {B}}^{-1}( 1+ {\varvec{\beta }}_{x_1} )\} \partial _{y_1} \\&= {\mathtt L}- (-\Delta )^{-1} \Pi _0^\perp \{{\mathcal {B}}^{-1}({\varvec{\beta }}_{x_1})\} \partial _{y_1} - {\mathcal {P}}_{-2}\, \Pi _0^\perp \{{\mathcal {B}}^{-1}( 1+ {\varvec{\beta }}_{x_1} )\} \partial _{y_1} . \end{aligned} \end{aligned}$$

In order to conjugate ${\mathcal {E}}_{0}$ we note that, by its definition in (4.4) and by (1.8), we have

$$\begin{aligned} {\mathcal {E}}_{0} = \nabla w \cdot {\mathfrak {B}}= w_{x_1} (-\Delta )^{-1} \partial _{x_2} - w_{x_2} (-\Delta )^{-1} \partial _{x_1}. \end{aligned}$$

Using the same computation in (5.37), we deduce that ${\mathcal {B}}_{\perp }^{-1} (-\Delta )^{-1} \partial _{x_m} {\mathcal {B}}_{\perp } \in {{\mathcal {O}}}{{\mathcal {P}}}{{\mathcal {M}}}_{s}^{-1}$, $m=1,2$, and consequently, using also (4.9), (4.10),

5.38 $$\begin{aligned} {\mathcal {B}}_{\perp }^{-1} {\mathcal {E}}_{0}\,{\mathcal {B}}_{\perp }&= \Pi _0^\perp \{{\mathcal {B}}^{-1} ( w_{x_1})\} {\mathcal {B}}_{\perp }^{-1} (-\Delta )^{-1} \partial _{x_2} {\mathcal {B}}_{\perp } - \Pi _0^\perp \{{\mathcal {B}}^{-1} ( w_{x_2})\} {\mathcal {B}}_{\perp }^{-1} (-\Delta )^{-1} \partial _{x_1} {\mathcal {B}}_{\perp }\nonumber \\&= \Pi _0^\perp \{{\mathcal {B}}^{-1}(\nabla w)\} \cdot {\mathcal {B}}_{\perp }^{-1} (-\Delta )^{-1} \nabla ^\perp {\mathcal {B}}_{\perp } \end{aligned}$$

is in ${{\mathcal {O}}}{{\mathcal {P}}}{{\mathcal {M}}}_{s}^{-1}$. We conclude that ${\mathcal {L}}_{1}:= {\mathcal {B}}_{\perp }^{-1} {\mathcal {L}}\,{\mathcal {B}}_{\perp }$ has the form (5.34), where ${\mathcal {E}}_{1}\in {{\mathcal {O}}}{{\mathcal {P}}}{{\mathcal {M}}}_{s}^{-1}$, recalling (5.37), (5.38), is given by

5.39 $$\begin{aligned} \begin{aligned} {\mathcal {E}}_{1}&:= \beta \big ( { -(-\Delta )^{-1} \Pi _0^\perp \{{\mathcal {B}}^{-1}({\varvec{\beta }}_{x_1})\} \partial _{y_1} - {\mathcal {P}}_{-2}\, \Pi _0^\perp \{{\mathcal {B}}^{-1}( 1+ {\varvec{\beta }}_{x_1} )\} \partial _{y_1} } \big ) \\&\quad + \lambda ^{\theta } \Pi _0^\perp \{{\mathcal {B}}^{-1}(\nabla w)\} \cdot {\mathcal {B}}_{\perp }^{-1} (-\Delta )^{-1} \nabla ^\perp {\mathcal {B}}_{\perp } . \end{aligned} \end{aligned}$$

The estimate (5.35) follows by (5.39), Lemma 2.2, Lemma 2.6-(*ii*), estimate (2.18), Lemma 2.16 and Proposition 5.1 with the smallness condition (5.4). The estimate (5.36) follows by similar arguments from the explicit expression (5.39) and we omit the details. Finally, by Lemma 2.9, 2.11 and Proposition 5.1, we obtain that ${\mathcal {L}}_{1}$ is real and momentum preserving. If *w* is $\textrm{odd}({\varphi }, x)$, then ${{\mathcal {L}}}_1$ is also reversible. This concludes the proof. $\square$

### Reduction to perturbative of the large remainder

The next goal is to conjugate the operator ${\mathcal {L}}_{1}$ in (5.34) through a series of transformation in order to reduce both the size and the order of the remainder ${\mathcal {E}}_{1}\in {{\mathcal {O}}}{{\mathcal {P}}}{{\mathcal {M}}}_{s}^{-1}$. We shall prove the following Proposition.

#### Proposition 5.6

Let $M\in \ {\mathbb {N}}$, $M > \frac{1-{\mathtt c}}{2(1-{\mathtt c}) - \alpha }$. There exist $\sigma _M= \sigma _{M}(\tau ,\nu ,M) \gg 0$ large enough such that, if $S>s_0 +\sigma _{M}$, there is $\delta \equiv \delta (S, M) \ll 1$ small enough such that, if $\lambda ^{\theta -1} \gamma ^{- 1} \leqslant \delta$ and if (4.2) is fullfilled with $\sigma _{0} = \sigma _M$, then the following hold. For any $\omega \in {\mathtt D}{\mathtt C}(2\gamma ,\tau )\cap \Lambda _{o}$, there exists a real, invertible operator $\mathbf{\Phi }_M \in {{\mathcal {O}}}{{\mathcal {P}}}{{\mathcal {M}}}_s^0$, $s_0 \leqslant s \leqslant S - \sigma _M$ satisfying

5.40 $$\begin{aligned} \begin{aligned}&|\mathbf{\Phi }_M^{\pm 1}|_{0, s}^{\textrm{Lip}(\gamma )} \lesssim _{s, M} 1 + \Vert {w} \Vert _{s + \sigma _M}^{\textrm{Lip}(\gamma )} , \quad \forall s_0 \leqslant s \leqslant S - \sigma _M, \\&\quad |\mathbf{\Phi }_M^{\pm 1}-\textrm{Id}|_{-M, s}^{\textrm{Lip}(\gamma )} \lesssim _{s, M} \Vert {w} \Vert _{s + \sigma _M}, \, \end{aligned} \end{aligned}$$

such that, for any $\omega \in {\mathtt D}{\mathtt C}(2\gamma ,\tau )\cap \Lambda _{o}$,

5.41 $$\begin{aligned} {{\mathcal {L}}}_M:= \mathbf{\Phi }_M^{- 1} {{\mathcal {L}}}_1 \mathbf{\Phi }_M = \lambda \, \omega \cdot \partial _\varphi + \beta \,{\mathtt L}+ {{\mathcal {Z}}}_M + {{\mathcal {E}}}_M, \end{aligned}$$

where ${{\mathcal {Z}}}_M \in {{\mathcal {O}}}{{\mathcal {P}}}{{\mathcal {M}}}^{- 1}_s$, $s \geqslant 0$, is a diagonal operator ${{\mathcal {Z}}}_M:= \textrm{diag}_{j \ne 0} z_M(j)$ and ${{\mathcal {E}}}_M \in {{\mathcal {O}}}{{\mathcal {P}}}{{\mathcal {M}}}^{- M}_s$, $s_0 \leqslant s \leqslant S - \sigma _M$ satisfy the estimates

5.42 $$\begin{aligned} \begin{aligned}&|{{\mathcal {Z}}}_M|_{- 1, s}^{\textrm{Lip}(\gamma )} \lesssim _{M} \lambda ^{\theta }, \quad \forall s \geqslant s_0, \quad \sup _{j \ne 0} |j| |z_M(j)|^{\textrm{Lip}(\gamma )} \lesssim _M \lambda ^{\theta }, \\&\quad |{{\mathcal {E}}}_M|_{- M, s}^{\textrm{Lip}(\gamma )} \lesssim _{s, M} \big ( \lambda ^{\theta -1}\gamma ^{-1} \big )^{M-1} \lambda ^{\theta } \Vert {w} \Vert _{s + \sigma _M}^{\textrm{Lip}(\gamma )} , \quad \forall s_0 \leqslant s \leqslant S - \sigma _M. \end{aligned} \end{aligned}$$

Let $$s_1 \geqslant s_0$$ and assume that $$ w_1, w_2$$ satisfy (4.2) with $$ \sigma _{0} \geqslant s_1 + \sigma _{M} $$. Then, for any $$\omega \in {\mathtt D}{\mathtt C}(2\gamma ,\tau )\cap \Lambda _{o}(w_1)\cap \Lambda _{o}(w_2)$$, one has

5.43 $$\begin{aligned} \begin{aligned}&|\Delta _{12} \mathbf{{\Phi }}_{M}^{\pm 1} |_{-M,s_1} \lesssim _{s_1, M} \Vert w_1 - w_2 \Vert _{s_1 + \sigma _{M}},\\&\quad | \Delta _{12} {\mathcal {E}}_{M}|_{-M,s_1}\lesssim _{s_1, M} \big ( \lambda ^{\theta -1}\gamma ^{-1} \big )^{M-1} \lambda ^{\theta } \Vert w_1 - w_2 \Vert _{s_1 + \sigma _{M}}. \end{aligned} \end{aligned}$$

Furthermore, the operators $\mathbf{{\Phi }}_{M}^{\pm 1}$ and ${\mathcal {L}}_{M}$ are real and momentum preserving. In addition, if we assume that $w({\varphi },x)=\textrm{odd}({\varphi },x)$, then the operators $\mathbf{{\Phi }}_{M}^{\pm 1}$ are reversibility preserving and ${\mathcal {L}}_{M}$ is reversible.

Proposition 5.6 is proved as a consequence of the following iterative procedure.

#### Lemma 5.7

Let $M\in \ {\mathbb {N}}$, $M > \frac{1-{\mathtt c}}{2(1-{\mathtt c}) - \alpha }$. There exist $\sigma _1< \sigma _2< \ldots < \sigma _M$ with $\sigma _{i}:=\sigma _{i}(\tau ,\nu )>0$ large enough such that, for any $S>s_0 +\sigma _{M}$, there exists $\delta \equiv \delta (S, M) \ll 1$ small enough such that, if $\lambda ^{\theta -1} \gamma ^{- 1} \leqslant \delta$ and if (4.2) is fullfilled with $\sigma _{0} = \sigma _M$, then the following holds. For any $m = 1, \ldots , M$, there exists a real operator ${{\mathcal {L}}}_m$ of the form

5.44 $$\begin{aligned} {{\mathcal {L}}}_m = \lambda \, \omega \cdot \partial _\varphi + \beta \,{\mathtt L}+ {{\mathcal {Z}}}_m + {{\mathcal {E}}}_m, \end{aligned}$$

where, for any $s_0 \leqslant s \leqslant S - \sigma _m$, ${{\mathcal {Z}}}_m \in {{\mathcal {O}}}{{\mathcal {P}}}{{\mathcal {M}}}^{- 1}_s$ is the diagonal operator ${{\mathcal {Z}}}_m = \textrm{diag}_{j \ne 0} z_m(j)$, ${{\mathcal {E}}}_m \in {{\mathcal {O}}}{{\mathcal {P}}}{{\mathcal {M}}}^{- m}_s$ and they satisfy the estimates

5.45 $$\begin{aligned} \begin{aligned}&|{{\mathcal {Z}}}_m|_{- 1, s}^{\textrm{Lip}(\gamma )} \lesssim _m \lambda ^{\theta } , \quad \sup _{j \ne 0} |j| |z_m(j)|^{\textrm{Lip}(\gamma )} \lesssim _m \lambda ^{\theta },\\&\quad |{{\mathcal {E}}}_m|_{- m, s}^{\textrm{Lip}(\gamma )} \lesssim _{s, m} \big ( \lambda ^{\theta -1} \gamma ^{- 1} \big )^{m - 1} \lambda ^{\theta } \Vert {w} \Vert _{s + \sigma _m}^{\textrm{Lip}(\gamma )} . \end{aligned} \end{aligned}$$

There exist real operators $\{ \Phi _{m} \}_{m=1}^{M - 1}$, with $\Phi _{m}:= \textrm{exp} ({\mathcal {X}}_{m})\in {{\mathcal {O}}}{{\mathcal {P}}}{{\mathcal {M}}}_{s}^{0}$, where

$$\begin{aligned} {\mathcal {X}}_{m}:= {\mathcal {X}}_{m}({\varphi }) = \sum _{\ell \in {\mathbb {Z}}^{\nu }\setminus \{0\}} {\widehat{{\mathcal {X}}}}_{m}(\ell )e^{\textrm{i}\,\ell \cdot {\varphi }} \in {{\mathcal {O}}}{{\mathcal {P}}}{{\mathcal {M}}}_{s}^{-m}, \end{aligned}$$

satisfying the estimates

5.46 $$\begin{aligned} \begin{aligned}&|{\mathcal {X}}_m|_{- m, s}^{\textrm{Lip}(\gamma )} \lesssim _{s, m} \big (\lambda ^{\theta -1} \gamma ^{- 1} \big )^m \Vert {w} \Vert _{s + \sigma _m}^{\textrm{Lip}(\gamma )}, \quad \forall s_0 \leqslant s \leqslant S - \sigma _m, \\&\quad |\Phi _m^{\pm 1}|_{0, s}^{\textrm{Lip}(\gamma )} \lesssim _{s, m} 1 + \Vert {w} \Vert _{s + \sigma _{m}}^{\textrm{Lip}(\gamma )}, \quad \forall s_0 \leqslant s \leqslant S - \sigma _m. \end{aligned} \end{aligned}$$

Moreover, for any $m = 2, \ldots , M - 1$ and any $\omega \in {\mathtt D}{\mathtt C}(2\gamma ,\tau )\cap \Lambda _{o}$, we have that

5.47 $$\begin{aligned} {\mathcal {L}}_{m}:= \Phi _{m-1}^{-1} {\mathcal {L}}_{m-1} \Phi _{m-1} . \end{aligned}$$

Let $s_1 \geqslant s_0$ and assume that $w_1,w_2$ satisfy (4.2) with $\sigma _{0} \geqslant s_1 + \sigma _{m}$. Then, for any $\omega \in {\mathtt D}{\mathtt C}(2\gamma ,\tau )\cap \Lambda _{o}(w_1)\cap \Lambda _{o}(w_2)$, we have

5.48 $$\begin{aligned} \begin{aligned}&|\Delta _{12} \Phi _{m}^{\pm 1} |_{-m,s_1} \lesssim _{s_1, m} \Vert w_1 - w_2 \Vert _{s_1 + \sigma _{m}} ,\\&\quad | \Delta _{12} {\mathcal {E}}_{m}|_{-m,s_1}\lesssim _{s_1, m} \big ( \lambda ^{\theta -1} \gamma ^{- 1} \big )^{m - 1} \lambda ^{\theta } \Vert w_1 - w_2 \Vert _{s_1 + \sigma _{m}}. \end{aligned} \end{aligned}$$

Furthermore, the operators $\mathbf{{\Phi }}_{m}^{\pm 1}$ and ${\mathcal {L}}_{m}$ are real and momentum preserving. In addition, if we assume that $w({\varphi },x)=\textrm{odd}({\varphi },x)$, then the operators $\mathbf{{\Phi }}_{m}^{\pm 1}$ are reversibility preserving and ${\mathcal {L}}_{m}$ is reversible.

#### Proof

We proceed by induction. The operator ${\mathcal {L}}_{1}$ in (5.34) is of the form (5.44) with ${\mathcal {Z}}_{1}=0$. By (5.35) and (5.36) we have that ${\mathcal {E}}_{1}$ satisfies (5.45) and (5.48). Finally, by Proposition 5.5 we have that ${\mathcal {L}}_{1}$ is reversible and momentum preserving.

We now assume that the claimed statements hold for $m = 1, \ldots , M - 1$ and we prove them for $m + 1$. We look for a trasformation of the form

$$\begin{aligned} \Phi _{m}:= \textrm{exp}({\mathcal {X}}_m) \in {{\mathcal {O}}}{{\mathcal {P}}}{{\mathcal {M}}}_{s}^{0}, \quad {\mathcal {X}}_{m}:= {\mathcal {X}}_{m}({\varphi }) = \sum _{\ell \in {\mathbb {Z}}^\nu \setminus \{0\}} {\widehat{{\mathcal {X}}}}_{m}(\ell ) e^{\textrm{i}\,\ell \cdot {\varphi }} \in {{\mathcal {O}}}{{\mathcal {P}}}{{\mathcal {M}}}_{s}^{-m}, \end{aligned}$$

where ${\widehat{{\mathcal {X}}}}_{m}$ has to be determined. To compute ${{\mathcal {L}}}_{m + 1} = \Phi _m^{- 1} {{\mathcal {L}}}_m \Phi _m$, we look separately at the conjugation of the three terms appearing in (5.44). By the standard Lie expansion, we get

5.49 $$\begin{aligned} \begin{aligned} \Phi _m^{- 1} \lambda \, \omega \cdot \partial _\varphi \Phi _m&= \lambda \, \omega \cdot \partial _\varphi + \lambda \, \omega \cdot \partial _\varphi {\mathcal {X}}_m(\varphi ) + {{\mathcal {Q}}}_m^{(1)}(\varphi ) , \\ {{\mathcal {Q}}}_m^{(1)}(\varphi )&= \int _0^1 (1 - \tau ) \textrm{exp}(- \tau {\mathcal {X}}_m) [\lambda \, \omega \cdot \partial _\varphi {\mathcal {X}}_m(\varphi ), {\mathcal {X}}_m(\varphi )] \textrm{exp}( \tau {\mathcal {X}}_m) \,\textrm{d}{\tau }, \\ \Phi _m^{- 1} ( \beta {\mathtt L}) \Phi _m&= \beta {\mathtt L}+ {{\mathcal {Q}}}_m^{(2)}, \\ {{\mathcal {Q}}}_m^{(2)}(\varphi )&:= \int _0^1 \textrm{exp}(- \tau {\mathcal {X}}_m(\varphi )) [\beta {\mathtt L}, {\mathcal {X}}_m(\varphi )] \textrm{exp}(\tau {\mathcal {X}}_m(\varphi )) \,\textrm{d}{\tau }, \\ \Phi _m^{- 1} {{\mathcal {Z}}}_m \Phi _m&= {{\mathcal {Z}}}_m + {{\mathcal {Q}}}_m^{(3)}, \\ {{\mathcal {Q}}}_m^{(3)}(\varphi )&:= \int _0^1 \textrm{exp}(- \tau {\mathcal {X}}_m(\varphi )) [{{\mathcal {Z}}}_m, {\mathcal {X}}_m(\varphi )] \textrm{exp}(\tau {\mathcal {X}}_m(\varphi )) \,\textrm{d}{\tau }, \\ \Phi _m^{- 1} {{\mathcal {E}}}_m \Phi _m&= {{\mathcal {E}}}_m + {{\mathcal {Q}}}_m^{(4)}, \\ {{\mathcal {Q}}}_m^{(4)}(\varphi )&:= \int _0^1 \textrm{exp}(- \tau {\mathcal {X}}_m(\varphi )) [{{\mathcal {E}}}_m(\varphi ), {\mathcal {X}}_m(\varphi )] \textrm{exp}(\tau {\mathcal {X}}_m(\varphi )) \,\textrm{d}{\tau }.\\ \end{aligned} \end{aligned}$$

The terms of order $- m$ in the expansion of ${{\mathcal {L}}}_{m + 1}= \Phi _m^{- 1} {{\mathcal {L}}}_m \Phi _m$ that we want to reduce are then given by $\lambda \, \omega \cdot \partial _\varphi {\mathcal {X}}_m(\varphi ) + {{\mathcal {E}}}_m(\varphi )$. Hence, we solve the homological equation

5.50 $$\begin{aligned} \lambda \, \omega \cdot \partial _\varphi {\mathcal {X}}_m(\varphi ) + {\mathcal E}_m(\varphi ) = \widehat{{\mathcal {E}}}_m(0), \quad \widehat{\mathcal E}_m(0):= \frac{1}{(2 \pi )^\nu } \int _{{\mathbb {T}}^\nu } {{\mathcal {E}}}_m(\varphi ) \,\textrm{d}{\varphi }, \end{aligned}$$

by defining, for any $\omega \in {\mathtt D}{\mathtt C}(2\gamma ,\tau )\cap \Lambda _{o}$,

$$\begin{aligned} {{\mathcal {X}}}_m(\varphi ) = - \sum _{\ell \ne 0} \frac{\widehat{\mathcal E}_m(\ell )}{\textrm{i}\lambda \, \omega \cdot \ell } e^{\textrm{i}\ell \cdot \varphi }. \end{aligned}$$

Hence the matrix elements of ${{\mathcal {X}}}_m$ are given by

$$\begin{aligned} \widehat{{\mathcal {X}}}_m(\ell )_j^{j'} = {\left\{ \begin{array}{ll} - \dfrac{\widehat{{\mathcal {E}}}_m(\ell )_j^{j'}}{\textrm{i}\lambda \,\omega \cdot \ell }, & \ell \ne 0, \quad j, j' \in {\mathbb {Z}}^2 \setminus \{ 0 \}, \quad \pi ^\top (\ell ) + j - j' = 0, \\ 0 & \text {otherwise.} \end{array}\right. } \end{aligned}$$

Then $\omega \in {\mathtt D}{\mathtt C}(2\gamma ,\tau )\cap \Lambda _{o}$, one has that

$$\begin{aligned} |\widehat{{\mathcal {X}}}_m(\ell )_j^{j'}| \lesssim \gamma ^{- 1} \lambda ^{- 1} \langle \ell \rangle ^\tau |\widehat{\mathcal E}_m(\ell )_j^{j'}| \end{aligned}$$

and if $\omega _1, \omega _2 \in {\mathtt D}{\mathtt C}(2\gamma ,\tau )\cap \Lambda _{o}$, one has

$$\begin{aligned} \begin{aligned} |\widehat{{\mathcal {X}}}_m(\ell ; \omega _1)_j^{j'} - \widehat{{\mathcal {X}}}_m(\ell ; \omega _2)_j^{j'}|&\lesssim \langle \ell \rangle ^{2 \tau + 1} \gamma ^{- 2} \lambda ^{- 1} |\widehat{{\mathcal {E}}}_m(\ell ; \omega _2)_j^{j'}| |\omega _1 - \omega _2| \\&\quad + \langle \ell \rangle ^\tau \gamma ^{- 1} \lambda ^{- 1} |\widehat{{\mathcal {E}}}_m(\ell ; \omega _1)_j^{j'} - \widehat{\mathcal E}_m(\ell ; \omega _2)_j^{j'}|. \end{aligned} \end{aligned}$$

By taking $S > s_0 + \sigma _{m} + 2 \tau + 1$, the latter two estimates, together with the Definition 2.5 imply that ${{\mathcal {X}}}_m \in {{\mathcal {O}}}{{\mathcal {P}}}{{\mathcal {M}}}_s^{- m}$ for any $s_0 \leqslant s \leqslant S - \sigma _{m} - 2 \tau - 1$ and

5.51 $$\begin{aligned} \begin{aligned}&|{{\mathcal {X}}}_m|_{- m, s}^{\textrm{Lip}(\gamma )} \lesssim _s \gamma ^{- 1} \lambda ^{- 1} |{{\mathcal {E}}}_m|_{- m, s + 2 \tau + 1}^{\textrm{Lip}(\gamma )} \\&\qquad \qquad {\mathop {\lesssim _{s, m}}\limits ^{(5.7)}} \lambda ^{- 1} \gamma ^{- 1} \big ( \lambda ^{\theta -1} \gamma ^{- 1} \big )^{m - 1} \lambda ^{\theta } \Vert {w} \Vert _{s + \sigma _m + 2 \tau + 1}^{\textrm{Lip}(\gamma )} \\&\qquad \qquad \lesssim _{s, m} \big ( \lambda ^{\theta -1} \gamma ^{- 1} \big )^{m } \Vert {w} \Vert _{s + \sigma _m + 2 \tau + 1}^{\textrm{Lip}(\gamma )}. \end{aligned} \end{aligned}$$

Together with Lemma 2.6-(*iv*) and using that $\lambda ^{\theta -1} \gamma ^{- 1} \ll 1$, we have that (5.51) implies, for any $s_0 \leqslant s \leqslant S - \sigma _m - 2 \tau - 1$,

5.52 $$\begin{aligned} \sup _{\tau \in [- 1, 1]}|\textrm{exp}(\tau {\mathcal {X}}_m)|_{0, s}^{\textrm{Lip}(\gamma )} \lesssim _{s, m} 1 + \Vert {w} \Vert _{s + \sigma _m + 2 \tau + 1}^{\textrm{Lip}(\gamma )} . \end{aligned}$$

By (5.49), we obtain that

5.53 $$\begin{aligned} \begin{aligned} {{\mathcal {L}}}_{m + 1}&= \Phi _m^{- 1} {{\mathcal {L}}}_m \Phi _m = \lambda \,\omega \cdot \partial _\varphi + \beta \, {\mathtt L}+ {{\mathcal {Z}}}_{m + 1} + {{\mathcal {E}}}_{m + 1} , \\ {{\mathcal {Z}}}_{m + 1}&:= {{\mathcal {Z}}}_m + \widehat{{\mathcal {E}}}_m(0), \\ {{\mathcal {E}}}_{m + 1}&:= {{\mathcal {Q}}}_m^{(1)} + {\mathcal Q}_m^{(2)} + {{\mathcal {Q}}}_m^{(3)} + {{\mathcal {Q}}}_m^{(4)}. \end{aligned} \end{aligned}$$

Properties and estimates of ${{\mathcal {Z}}}_{m + 1}$. Since ${{\mathcal {E}}}_m$ is momentum preserving, by Lemma 2.13 we have that the time-independent operator $\widehat{{\mathcal {E}}}(0)$ is diagonal and hence also the operator ${{\mathcal {Z}}}_{m+1} = \textrm{diag}_{j \ne 0} z_{m+1}(j)$ is a diagonal operator with $z_{m + 1}(j):= z_m(j) + \widehat{\mathcal E}_m(0)_j^{j}$, $j \in {\mathbb {Z}}^2 {\setminus } \{ 0 \}$. Furthermore, by Lemma 2.6-(*v*) and by the induction estimates (5.45) (using also the ansatz (4.2)), we get that the operator ${{\mathcal {Z}}}_{m + 1} \in {{\mathcal {O}}}{{\mathcal {P}}}{{\mathcal {M}}}^{- 1}_s$ for any $s \geqslant s_0$ and it satisfies, using also $\lambda ^{\theta -1} \gamma ^{- 1} \ll 1$ by (4.7),

5.54 $$\begin{aligned} \begin{aligned} |{{\mathcal {Z}}}_{m + 1}|_{- 1, s}^{\textrm{Lip}(\gamma )}&\lesssim |{{\mathcal {Z}}}_m|_{- 1, s}^{\textrm{Lip}(\gamma )} + |\widehat{{\mathcal {E}}}_m(0)|_{- m, s} \lesssim |{{\mathcal {Z}}}_m|_{- 1, s}^{\textrm{Lip}(\gamma )} + |{{\mathcal {E}}}_m|_{- m, s_0}^{\textrm{Lip}(\gamma )} \\&\lesssim _m \lambda ^{\theta } + \big ( \lambda ^{\theta -1} \gamma ^{- 1}\big )^{m - 1} \lambda ^{\theta } \lesssim _m \lambda ^{\theta }, \end{aligned} \end{aligned}$$

which implies that

$$\begin{aligned} \sup _{j \ne 0} |j| |z_{m + 1}(j)|^{\textrm{Lip}(\gamma )} \lesssim _m \lambda ^{\theta }. \end{aligned}$$

Properties and estimates of ${{\mathcal {E}}}_{m + 1}$. We now estimate the remainder ${{\mathcal {E}}}_{m + 1}$ in (5.53). We have to analyze the four terms ${{\mathcal {Q}}}_m^{(1)}, {{\mathcal {Q}}}_m^{(2)}, {{\mathcal {Q}}}_m^{(3)}, {{\mathcal {Q}}}_m^{(4)}$ in (5.49). First, by (5.50), we note that

$$\begin{aligned} {{\mathcal {Q}}}_m^{(1)} = \int _0^1 (1 - \tau ) \textrm{exp}(- \tau {\mathcal {X}}_m) { [{{\mathcal {Z}}}_{m + 1}-{{\mathcal {Z}}}_{m} - {{\mathcal {E}}}_m, {\mathcal {X}}_m] } \textrm{exp}( \tau {\mathcal {X}}_m) \,\textrm{d}{\tau }. \end{aligned}$$

By the estimates (5.45), (5.51), (5.54), using also (2.21) Lemma 2.6-(*ii*) the ansatz (4.2), we get that, taking $S > s_0 + \sigma _m + m + 2 \tau + 1$, the operators $[{{\mathcal {Z}}}_{m + 1}, {\mathcal {X}}_m] \,, {[{{\mathcal {Z}}}_{m}, {\mathcal {X}}_m] } \,,[{\mathtt L}, {\mathcal {X}}_m] \,, \in {{\mathcal {O}}}{{\mathcal {P}}}{{\mathcal {M}}}_s^{- m - 1}, [{\mathcal E}_m, {\mathcal {X}}_m] \in {{\mathcal {O}}}{{\mathcal {P}}}{{\mathcal {M}}}_s^{- 2\,m} {\subseteq } {{\mathcal {O}}}{{\mathcal {P}}}{{\mathcal {M}}}^{- m - 1}_s$ for any $s_0 \leqslant s \leqslant S - \sigma _m - m- 2 \tau - 1$ and they satisfy the following estimates, recalling also that $\lambda ^{\theta -1} \gamma ^{- 1} \ll 1$ by (4.7):

$$\begin{aligned} \begin{aligned} | [{{\mathcal {Z}}}_{m + 1}, {\mathcal {X}}_m]|_{- m - 1, s}^{\textrm{Lip}(\gamma )}&\lesssim _{s, m} \lambda ^{\theta } \big ( \lambda ^{\theta -1} \gamma ^{- 1} \big )^m \Vert {w} \Vert _{s + \sigma _m + m + 2 \tau + 1}^{\textrm{Lip}(\gamma )}, \\ { | [{{\mathcal {Z}}}_{m}, {\mathcal {X}}_m]|_{- m - 1, s}^{\textrm{Lip}(\gamma )} }&\lesssim _{s, m} \lambda ^{\theta } \big ( \lambda ^{\theta -1} \gamma ^{- 1} \big )^m \Vert {w} \Vert _{s + \sigma _m + m + 2 \tau + 1}^{\textrm{Lip}(\gamma )}, \\ |[{{\mathcal {E}}}_m, {\mathcal {X}}_m]|_{- m - 1, s}^{\textrm{Lip}(\gamma )}&\leqslant |[{{\mathcal {E}}}_m, {\mathcal {X}}_m]|_{- 2m, s}^{\textrm{Lip}(\gamma )} \\&\lesssim _{s, m} \lambda ^{\theta } \big ( \lambda ^{\theta -1} \gamma ^{- 1}\big )^{m - 1} \big ( \lambda ^{\theta -1} \gamma ^{- 1}\big )^{m} \Vert {w} \Vert _{s + \sigma _m + m + 2 \tau + 1}^{\textrm{Lip}(\gamma )} \\&\lesssim _{s, m} \lambda ^{\theta } \big ( \lambda ^{\theta -1} \gamma ^{- 1}\big )^{m} \Vert {w} \Vert _{s + \sigma _m + m + 2 \tau + 1}^{\textrm{Lip}(\gamma )}, \\ |[{\mathtt L}, {\mathcal {X}}_m]|_{- m - 1, s}^{\textrm{Lip}(\gamma )}&\lesssim _{s, m} \big ( \lambda ^{\theta -1} \gamma ^{- 1}\big )^{m} \Vert {w} \Vert _{s + \sigma _m + 2 \tau + 1}^{\textrm{Lip}(\gamma )} \\&\lesssim _{s, m} \lambda ^{\theta } \big ( \lambda ^{\theta -1} \gamma ^{- 1}\big )^{m} \Vert {w} \Vert _{s + \sigma _m + 2 \tau + 1}^{\textrm{Lip}(\gamma )}. \end{aligned} \end{aligned}$$

Together with (5.52), and Lemma 2.6-(*ii*), the latter estimates imply that, for $\sigma _{m + 1} \geqslant \sigma _m + m + 2 \tau + 2$, $\Vert {w} \Vert _{s_0 + \sigma _{m + 1}}^{\textrm{Lip}(\gamma )} \lesssim 1$ and $S > s_0 + \sigma _{m + 1}$, the operator ${{\mathcal {E}}}_{m + 1} \in {{\mathcal {O}}}{{\mathcal {P}}}{{\mathcal {M}}}_s^{- m - 1}$ for any $s_0 \leqslant s \leqslant S - \sigma _{m + 1}$ and it satisfies

$$\begin{aligned} \begin{aligned}&|{{\mathcal {E}}}_{m + 1}|_{- m - 1, s}^{\textrm{Lip}(\gamma )} \lesssim _{s, m} \lambda ^{\theta } \big ( \lambda ^{\theta -1} \gamma ^{- 1} \big )^m \Vert {w} \Vert _{s + \sigma _{m + 1}}^{\textrm{Lip}(\gamma )}. \end{aligned} \end{aligned}$$

The estimates in (5.48) follow by similar arguments and we omit the details. By Lemma 2.11 and the inductive hypotheses on ${\mathcal {L}}_{m}$ and $\Phi _{m}$, we obtain that $\Phi _{m+1}, {{\mathcal {L}}}_{m + 1}$ are momentum preserving. If $w = \textrm{odd}({\varphi }, x)$ then by Lemma 2.9, $\Phi _m$ is reversibility preserving and ${{\mathcal {L}}}_{m + 1}, {{\mathcal {E}}}_{m + 1}, {{\mathcal {Z}}}_{m + 1}$ are reversible operators. The proof of the claimed statements at the step $m + 1$ is then concluded. $\square$

#### Proof of Proposition 5.6

Let $\mathbf{\Phi }_{1}:=\textrm{Id}$ and $\mathbf{\Phi }_M:= \Phi _{1} \circ \Phi _{2} \circ \ldots \circ \Phi _{M - 1}$ for $M\geqslant 2$. By Lemma 5.7, Lemma 2.6-(*ii*) and the estimates (5.46), (5.48), we obtain that $\mathbf{{\Phi }}_{M}$ satisfies the estimates (5.40), (5.43). Moreover, if $\omega \in {\mathtt D}{\mathtt C}(2\gamma ,\tau )\cap \Lambda _{o}$, then the conjugation (5.41) holds with ${{\mathcal {Z}}}_M$ satisfying the estimate in (5.42), using also Lemma 2.6-(*v*), and ${{\mathcal {E}}}_M$ satisfying the estimate, for any $s_0 \leqslant s \leqslant S - \sigma _M$,

$$\begin{aligned} |{{\mathcal {E}}}_M|_{- M, s}^{\textrm{Lip}(\gamma )} \lesssim _{s, M} \big ( \lambda ^{\theta -1} \gamma ^{- 1} \big )^{M - 1} \lambda ^{\theta } \Vert {w} \Vert _{s + \sigma _M}^{\textrm{Lip}(\gamma )} . \end{aligned}$$

Then, the desired bound on ${{\mathcal {E}}}_M$ in (5.42) follows since, using $M > \frac{1-{\mathtt c}}{2(1-{\mathtt c}) - \alpha } = \frac{1-{\mathtt c}}{1-{\mathtt c}-\theta }$, $\lambda \gg 1$ large enough, and recalling (4.5), (4.6), we have, if we assume $\gamma =\lambda ^{-{\mathtt c}}$,

$$\begin{aligned} \big ( \lambda ^{\theta -1} \gamma ^{- 1} \big )^{M - 1} \lambda ^{\theta } = \lambda ^{M\theta -(M-1)(1-{\mathtt c})} < 1. \end{aligned}$$

Finally, by Lemma 2.11 and Proposition 5.5, we obtain that $\mathbf{{\Phi }}_{M}$, ${\mathcal L}_M$ are momentum preserving. Moreover, if $w = \textrm{odd}({\varphi }, x)$, then by Lemma 2.9, $\mathbf{{\Phi }}_{M}$ is reversibility preserving and ${{\mathcal {L}}}_M$ is reversible. The proof of the Proposition is then concluded. $\square$

### KAM perturbative reduction

We are now in position to reduce the operator ${\mathcal {L}}_{M}$ in (5.47) with a KAM reducibility iteration. To the purposes of the KAM reducibility, we define

5.55 $$\begin{aligned} \textbf{L}_{0}: ={\mathcal {L}}_{M} = \lambda \,\omega \cdot \partial _{{\varphi }} + \textbf{D}_{0} + \textbf{E}_{0} \end{aligned}$$

where:

$\bullet$ The diagonal operator $\textbf{D}_{0}$ is given by

$$\begin{aligned} \begin{aligned}&\textbf{D}_{0}:= \beta \,{\mathtt L}+ \textbf{Z}_{0} = {{\,\textrm{diag}\,}}_{j\in {\mathbb {Z}}^2\setminus \{0\}} \mu _{0}(j), \quad \mu _{0}(j) = \textrm{i}\,\beta {\mathtt L}(j) + {\mathtt z}_{0}(j) , \\&\quad \textbf{Z}_{0}:= {\mathcal {Z}}_{M}:= {{\,\textrm{diag}\,}}_{j\in {\mathbb {Z}}^2\setminus \{0\}} {\mathtt z}_{0}(j), \quad {\mathtt z}_{0} (j):= z_{M}(j) , \ \ j \in {\mathbb {Z}}^2\setminus \{0\}, \end{aligned} \end{aligned}$$

with ${\mathtt L}(j)\in {\mathbb {R}}$ and ${\mathtt z}_0 ( j )\in {\mathbb {C}}$ defined respectively in (1.5) and in Proposition 5.6 (if we assume reversibility conditions, then ${\mathtt z}_{0}(j)\in \textrm{i}{\mathbb {R}}$);

$\bullet$ For any $s\in [s_0,S-\sigma _{M}]$, the operator $\textbf{E}_{0}:= {\mathcal {E}}_{M} \in {{\mathcal {O}}}{{\mathcal {P}}}{{\mathcal {M}}}_{s}^{- M}$ satisfies the estimate (see (5.42))

$$\begin{aligned} | \textbf{E}_{0} |_{-M,s}^{\textrm{Lip}(\gamma )} \lesssim _{s, M} \lambda ^{M(\theta -1)+1} \gamma ^{-(M-1)} \Vert w \Vert _{s+\sigma _{M}} \end{aligned}$$

and if $$s_1 \geqslant s_0$$, $$ w_1, w_2$$ satisfy (4.2) with $$ \sigma _{0} \geqslant s_1 + \sigma _{M} $$, then (see (5.43))

$$\begin{aligned} |\Delta _{12} \textbf{E}_0|_{- M, s_1} \lesssim _{s_1, M} \lambda ^{M(\theta -1)+1} \gamma ^{-(M-1)} \Vert w_1 - w_2 \Vert _{s_1 + \sigma _{M}}. \end{aligned}$$

Note that, with $M > \frac{1-{\mathtt c}}{2(1-{\mathtt c})-\alpha }$ and assuming $\gamma =\lambda ^{-{\mathtt c}}$, recalling also (4.5), (4.6), the operator $\textbf{E}_{0}$ has small size with respect to $\lambda \gg 1$ large enough.

Given $\tau , {\mathtt N}_0 > 0$, we fix the constants

5.56 $$\begin{aligned} \begin{aligned}&M:= \max \{2 \tau , \tfrac{1-{\mathtt c}}{2(1-{\mathtt c})-\alpha } \}\ + 1, \quad {\mathtt a}:= \textrm{max}\{3(2 \tau \! + \!M \!+ 1) + 1,\, \chi (\tau + \tau ^2 + 2) \} , \quad {\mathtt b}:=\! {\mathtt a}+ 1 , \\&\quad \tau _{1}:= 4\tau +2 +M , \quad \Sigma ({\mathtt b}):= \sigma _{M} +{\mathtt b}, \quad S > s_0 + \Sigma ({\mathtt b}), \\&{\mathtt N}_{- 1}:= 1 , \quad {\mathtt N}_n:= {\mathtt N}_0^{\chi ^n}, \quad n \geqslant 0, \quad \chi := 3/2 , \end{aligned} \end{aligned}$$

where *M*, $\sigma _{M }$ are introduced in Proposition 5.6.

#### Remark 5.8

Let us describe of the parameters introduced in (5.56) and their role in the following Proposition 5.9. The parameter ${\mathtt a}>0$ appears in the negative exponent of the first estimate in (5.64) and measures how the low regularity norm of the remainder is fastly decreasing at each iteration. The parameter ${\mathtt b}>0$ appears in the regularity index $s+{\mathtt b}$ of the second estimate in (5.64), which is the high regularity norm of the remainder that is allowed to diverge. The parameter $\tau _1>0$ appears as an exponent in the smallness condition (5.59). The parameter $\Sigma ({\mathtt b})>0$ accounts for the loss of derivatives of the KAM iteration.

By Proposition 5.6, replacing *s* by $s + {\mathtt b}$ in (5.42) and having $\textbf{Z}_{0} = \textrm{diag}_{j \in {\mathbb {Z}}^2 {\setminus } \{ 0 \}} {\mathtt z}_{0}(j)$ diagonal, and by the ansatz (4.2), one gets the initialization conditions for the KAM reducibility, for any $s_0\leqslant s\leqslant S-\Sigma ({\mathtt b})$,

5.57 $$\begin{aligned} \begin{aligned}&\sup _{j \in {\mathbb {Z}}^2 \setminus \{ 0 \}} |j| |{\mathtt z}_{0}(j)|^{\textrm{Lip}(\gamma )} \lesssim \lambda ^{\theta }, \\&\quad |\textbf{E}_{0}|_{- M, s + {\mathtt b}}^{\textrm{Lip}(\gamma )} \lesssim _{s} \lambda ^{M(\theta -1)+1} \gamma ^{-(M-1)} \Vert w \Vert _{s + \Sigma ({\mathtt b})}^{\textrm{Lip}(\gamma )}, \\&\quad |\textbf{E}_{0}|_{- M, s_0 + {\mathtt b}}^{\textrm{Lip}(\gamma )} \lesssim _{s} \lambda ^{M(\theta -1)+1} \gamma ^{-(M-1)} , \\&\quad \text {and if} \quad w_1, w_2 \quad \text { satisfy } (4) \text { with} \quad \sigma _{0} = \Sigma ({\mathtt b}) \quad \text {then} \\&\quad |\Delta _{12} \textbf{E}_0 |_{- M, s_0 + \mathtt b} \lesssim \lambda ^{M(\theta -1)+1} \gamma ^{-(M-1)} \Vert w_1 - w_2 \Vert _{s_0 + \Sigma (\mathtt b)}. \end{aligned} \end{aligned}$$

By the definition of *M* in (5.56), one has that $M>\frac{1-{\mathtt c}}{2(1-{\mathtt c})-\alpha }> \frac{1}{1-\theta }$. We work in the regime $\lambda \gg 1$ and recalling (4.5), (4.6), we define the small parameters (recalling (5.2))

5.58 $$\begin{aligned} \varepsilon := \lambda ^{\theta -1}, \quad \varepsilon _{M}:= \lambda ^{M(\theta -1)+1} = \varepsilon ^{M-1} \lambda ^{\theta } . \end{aligned}$$

#### Proposition 5.9

(**Reducibility**) Let $S > s_0 + \Sigma ({\mathtt b})$, with the notation of (5.56). There exist ${\mathtt N}_{0}:= {\mathtt N}_{0}(S, \tau ,\nu ) > 0$ large enough and $\delta := \delta (S, \tau ,\nu ) \in (0, 1)$ small enough such that, if (4.2) holds with $\sigma = \Sigma ({\mathtt b})$ and

5.59 $$\begin{aligned} {\mathtt N}_{0}^{\tau _1} \varepsilon ^{M}\gamma ^{-M} \leqslant \delta , \end{aligned}$$

then the following statements hold for any integer $n \geqslant 0$.

$\mathbf{(S1)}_n$ There exists a real, momentum preserving operator

5.60 $$\begin{aligned} \begin{aligned}&\textbf{L}_{n}:= \lambda \,\omega \cdot \partial _\varphi + \textbf{D}_{n} + \textbf{E}_{n}: H^{s + 1}_0 \rightarrow H^s_0 , \\&\quad \textbf{D}_{n}:= \beta \, {\mathtt L}+ \textbf{Z}_{n}= \textrm{diag}_{j \in {\mathbb {Z}}^2 \setminus \{ 0 \}} \mu _{n}(j) , \\&\quad \textbf{Z}_{n} = \textrm{diag}_{j \in {\mathbb {Z}}^2 \setminus \{ 0 \}} {\mathtt z}_{n}(j), \quad \mu _{n}(j):= \textrm{i}\, \beta {\mathtt L}(j)+ {\mathtt z}_{n}(j), \end{aligned} \end{aligned}$$

defined for any $\omega \in \Omega _{n}^\gamma$, where we define $\Omega _{0}^\gamma := \Omega _{0}^\gamma (w):= {\mathtt D}{\mathtt C}(2\gamma , \tau )\cap \Lambda _{o}$ for $n=0$ and, for $n \geqslant 1$,

5.61 $$\begin{aligned} \begin{aligned}&\Omega _{n}^\gamma :=\Omega _{n}^\gamma (w):= \Big \{ \omega \in \Omega _{n - 1}^\gamma : \, |\textrm{i}\,\lambda \,\omega \cdot \ell + \mu _{n - 1}(j) - \mu _{n - 1}(j') | \geqslant \frac{\lambda \, \gamma }{\langle \ell \rangle ^\tau |j'|^\tau }, \\&\qquad \ \forall \,\ell \in {\mathbb {Z}}^\nu \setminus \{ 0 \} , \ \ j,j' \in {\mathbb {Z}}^2 \setminus \{ 0 \}, \ \ \pi ^\top ( \ell ) + j-j' =0, \ \ |\ell |\leqslant {\mathtt N}_{n-1} \Big \}. \end{aligned} \end{aligned}$$

For any $j \in {\mathbb {Z}}^2 \setminus \{ 0 \}$, the eigenvalues $\mu _{n}(j)= \mu _{n}(j;\omega )$ satisfy the estimates

5.62 $$\begin{aligned}&|{\mathtt z}_{n}(j)|^{\textrm{Lip}(\gamma )} \lesssim \lambda ^{\theta } |j|^{-1}\,, \quad | {\mathtt z}_{n}(j)- {\mathtt z}_{0}(j) |^{\textrm{Lip}(\gamma )} \lesssim \varepsilon _{M} \gamma ^{-(M-1)} |j|^{- M} \,, \end{aligned}$$

5.63 $$\begin{aligned}&| {\mathtt z}_{n}(j) - {\mathtt z}_{n - 1}(j)|^{\textrm{Lip}(\gamma )} \lesssim \varepsilon _{M} \gamma ^{-(M-1)} \, {\mathtt N}_{n - 2}^{- {\mathtt a}}\, |j|^{- M} \quad \ \text {when } \quad n\geqslant 1 \,. \end{aligned}$$

The operator $\textbf{E}_{n}$ is real and momentum preserving, satisfying, for any $s_0\leqslant s\leqslant S-\Sigma ({\mathtt b})$,

5.64 $$\begin{aligned} \begin{aligned}&|\textbf{E}_{n} |_{- M, s}^{\textrm{Lip}(\gamma )} \leqslant C_*(s) \varepsilon _{M} \gamma ^{-(M-1)} \, {\mathtt N}_{n - 1}^{- {\mathtt a}} \Vert w \Vert _{s + \Sigma ({\mathtt b})}^{\textrm{Lip}(\gamma )} , \\&\quad |\textbf{E}_{n} |_{-M, s + {\mathtt b}}^{\textrm{Lip}(\gamma )} \leqslant C_*(s) \varepsilon _{M} \gamma ^{-(M-1)} \, {\mathtt N}_{n - 1} \Vert w \Vert _{s + \Sigma ({\mathtt b})}^{\textrm{Lip}(\gamma )} , \end{aligned} \end{aligned}$$

for some constant $C_* (s) > 0$ large enough.

When $n \geqslant 1$, there exists an invertible, real, and momentum preserving map $\Phi _{n -1} = \textrm{exp}(\Psi _{n - 1})$, such that, for any $\omega \in \Omega _{n}^\gamma$,

5.65 $$\begin{aligned} \textbf{L}_{n} = \Phi _{n - 1}^{- 1} \textbf{L}_{n-1} \Phi _{n - 1}. \end{aligned}$$

Moreover, for any $s_0\leqslant s\leqslant S-\Sigma ({\mathtt b})$, the map $\Psi _{n - 1}: H^s_0 \rightarrow H^s_0$ satisfies

5.66 $$\begin{aligned} \begin{aligned} |\Psi _{n - 1}|_{0, s}^{\textrm{Lip}(\gamma )}&\lesssim _s \varepsilon ^{M} \gamma ^{- M} {\mathtt N}_{n - 1}^{2 \tau + 1} {\mathtt N}_{n - 2}^{- {\mathtt a}} \Vert w \Vert _{s + \Sigma ({\mathtt b})}^{\textrm{Lip}(\gamma )}, \\ |\Psi _{n - 1} |_{0, s + {\mathtt b}}^{\textrm{Lip}(\gamma )}&\lesssim _s \varepsilon ^{M} \gamma ^{- M} {\mathtt N}_{n - 1}^{2 \tau + 1} {\mathtt N}_{n - 2} \Vert w \Vert _{s + \Sigma ({\mathtt b})}^{\textrm{Lip}(\gamma )}. \end{aligned} \end{aligned}$$

In addition, if we assume that $w({\varphi },x)=\textrm{odd}({\varphi },x)$, then the operators $\textbf{L}_{n}$, $\textbf{E}_{n}$ are reversible, the eigenvalues $\mu _{n}(j)= \mu _{n}(j;\omega )$, for any $j \in {\mathbb {Z}}^2 {\setminus } \{ 0 \}$, are purely imaginary, satisfying

5.67 $$\begin{aligned} \begin{aligned}&\mu _n(j) = - \mu _n(- j) = - \overline{\mu _n( j)} , \ \ \text {or equivalently} \\&\quad {\mathtt z}_{n}(j) = - {\mathtt z}_{n}(- j)= - \overline{{\mathtt z}_{n}( j)}, \end{aligned} \end{aligned}$$

and, for $n\geqslant 1$, the map $\Phi _{n - 1}$ is reversibility preserving.

$\mathbf{(S2)}_n$ For all $j \in {\mathbb {Z}}^2 \setminus \{ 0 \}$, there exists a Lipschitz extension of the eigenvalues $\mu _n(j;\,\cdot \,):\Omega _{n}^\gamma \rightarrow {\mathbb {C}}$ to the set ${\mathtt D}{\mathtt C}(2\gamma , \tau )\cap \Lambda _{o}$, denoted by $\widetilde{\mu }_n(j;\,\cdot \,): {{\mathtt D}{\mathtt C}(2\gamma , \tau ) \cap \Lambda _{o}}\rightarrow {\mathbb {C}}$, satisfying, for $n \geqslant 1$,

5.68 $$\begin{aligned} |{{\widetilde{\mu }}}_n(j) - {{\widetilde{\mu }}}_{n - 1}(j) |^{\textrm{Lip}(\gamma )} \lesssim |j|^{- M} |\textbf{E}_{n - 1}|_{-M,s_0}^{\textrm{Lip}(\gamma )} \lesssim \varepsilon _{M} \gamma ^{-(M-1)} \, {\mathtt N}_{n - 2}^{- {\mathtt a}}\, |j|^{- M}. \end{aligned}$$

$\mathbf{(S3)_{n}}$ Let $w_1(\omega )$, $w_2(\omega )$ satisfying the ansatz (4.2) be such that $\textbf{E}_0(w_1)$, $\textbf{E}_0(w_2 )$ satisfy (5.57). Then for all $\omega \in \Omega _n^{\gamma _1}(w_1) \cap \Omega _n^{\gamma _2}(w_2)$ with $\gamma _1, \gamma _2 \in [\gamma /2, 2 \gamma ]$, the following estimates hold:

5.69 $$\begin{aligned} \begin{aligned}&|\Delta _{12} \textbf{E}_n |_{- M, s_0} \leqslant C_*(s) {\mathtt N}_{n - 1}^{- {\mathtt a}} \Vert w_1 - w_2\Vert _{s_0 + \Sigma ({\mathtt b})} , \\&\quad |\Delta _{12} \textbf{E}_n |_{- M, s_0 + {\mathtt b}} \leqslant C_*(s) {\mathtt N}_{n - 1}  \Vert w_1 - w_2\Vert _{s_0 + \Sigma ({\mathtt b})} . \end{aligned} \end{aligned}$$

Moreover for $n \geqslant 1$, for all $j \in {\mathbb {Z}}^2 \setminus \{ 0 \}$,

5.70 $$\begin{aligned} \begin{aligned} \big |\Delta _{12}(\mu _n(j) - \mu _{n - 1}(j)) \big |&= \big |\Delta _{12}({\mathtt z}_n(j) - {\mathtt z}_{n - 1}(j)) \big | \\&\lesssim \varepsilon _{M} \gamma ^{-(M-1)} \, {\mathtt N}_{n - 2}^{- {\mathtt a}} \, |j|^{- M} \Vert w_1 - w_2 \Vert _{ s_0 + \Sigma ({\mathtt b})}, \\ |\Delta _{12} \mu _n(j)| = |\Delta _{12} {\mathtt z}_{n}(j)|&\lesssim \lambda ^{\theta } |j|^{- 1} \Vert w_1 - w_2 \Vert _{ s_0 + \Sigma ({\mathtt b})}. \end{aligned} \end{aligned}$$

$\mathbf{(S4)_{n}}$ Let $w_1$, $w_2$ be like in $\mathbf{(S3)_{n}}$ and $0 < \rho \leqslant \gamma /2$. Then:

5.71 $$\begin{aligned} K \varepsilon \rho ^{- 1} {\mathtt N}_{n - 1}^{\tau + \tau ^2} \Vert w_1 - w_2 \Vert _{s_0 + \Sigma ({\mathtt b})} \leqslant 1 \quad \Longrightarrow \quad \Omega _n^\gamma (w_1) \subseteq \Omega _n^{\gamma - \rho }(w_2) \,. \end{aligned}$$

#### Proof

Proof of $\mathbf{(S1)}_0-\mathbf{(S4)}_0$. The claimed properties follow directly from Proposition 5.6, recalling (5.55), (5.57), (5.58) and the definition of $\Omega _{0}^\gamma := {\mathtt D}{\mathtt C}(2\gamma , \tau )\cap \Lambda _{o}$.

By induction, we assume the the claimed properties $\mathbf{(S1)}_n$-$\mathbf{(S4)}_n$ hold for some $n \geqslant 0$ and we prove them at the step $n + 1$.

Proof of $\mathbf{(S1)}_{n+1}$. Let $\Phi _n = \textrm{exp}( \Psi _n)$ where $\Psi _n$ is an operator of order $- M$ to be determined. By the Lie expansion, we compute

5.72 $$\begin{aligned} \begin{aligned} \textbf{L}_{n + 1}&= \Phi _n^{- 1}\textbf{L}_{n} \Phi _n = \lambda \, \omega \cdot \partial _\varphi + \textbf{D}_n + \lambda \, \omega \cdot \partial _\varphi \Psi _n + [\textbf{D}_n, \Psi _n] + \Pi _{{\mathtt N}_n} \textbf{E}_n + \textbf{E}_{n + 1} , \\ \textbf{E}_{n + 1}&:= \Pi _{{\mathtt N}_n}^\bot \textbf{E}_n + \int _0^1 (1 - \tau ) \textrm{exp}(- \tau \Psi _n) \,[\lambda \,\omega \cdot \partial _\varphi \Psi _n + [\textbf{D}_n, \Psi _n], \Psi _n] \, \textrm{exp}(\tau \Psi _n) \,\textrm{d}{\tau }\\&\quad + \int _0^1 \textrm{exp}(- \tau \Psi _n) [\textbf{E}_n, \Psi _n] \textrm{exp}(\tau \Psi _n) \,\textrm{d}{\tau }, \end{aligned}\nonumber \\ \end{aligned}$$

where $\textbf{D}_{n}:= \beta \,{\mathtt L}+ \textbf{Z}_{n}$ and the projectors $\Pi _{N}$, $\Pi _{N}^\bot$ are defined in (2.16). Our purpose is to find a map $\Psi _n$ solving the *homological equation*

5.73 $$\begin{aligned} \lambda \,\omega \cdot \partial _\varphi \Psi _n + [\textbf{D}_{n}, \Psi _n] + \Pi _{{\mathtt N}_{n}} \textbf{E}_{n} = {\widehat{\textbf{E}}}_n(0) , \end{aligned}$$

where ${\widehat{\textbf{E}}}_n(0):= \frac{1}{(2 \pi )^\nu } \int _{{\mathbb {T}}^\nu } \textbf{E}_n(\varphi ) \,\textrm{d}{\varphi }$ is a diagonal operator by Lemma 2.13, since $\textbf{E}_{n}({\varphi })$ is a momentum preserving operator by induction assumption.

By (2.9) and (5.60), the homological equation (5.73) is equivalent to

5.74 $$\begin{aligned} \begin{aligned}&\big ( \textrm{i}\,\lambda \, \,\omega \cdot \ell + \mu _{n}(j) - \mu _{n}(j') \big ) {{\widehat{\Psi }}}_{n}(\ell )_j^{j'} + \widehat{\textbf{E}_{n}}(\ell )_j^{j'} = 0, \\&\quad \ell \in {\mathbb {Z}}^\nu \setminus \{0\}, \ \ |\ell | \leqslant {\mathtt N}_n,\, \ \ j, j' \in {\mathbb {Z}}^2 \setminus \{ 0 \}, \ \ \pi ^\top (\ell ) + j - j' = 0. \end{aligned} \end{aligned}$$

Therefore, we define the linear operator $\Psi _{n}$ by

5.75 $$\begin{aligned} {{\widehat{\Psi }}}_{n} (\ell )_j^{j'}:= {\left\{ \begin{array}{ll} - \dfrac{\widehat{\textbf{E}_{n}}(\ell )_j^{j'} }{ \textrm{i}\,\lambda \, \omega \cdot \ell + \mu _{n}(j) - \mu _{n}(j') }, &  \begin{matrix} \ell \in {\mathbb {Z}}^{\nu }\setminus \{0\} , \quad j,j'\in {\mathbb {Z}}^2\setminus \{0\}, \\ |\ell | \leqslant {\mathtt N}_{n}, \quad \pi ^\top (\ell ) + j-j'=0, \end{matrix} \\ 0 &  \text {otherwise}. \end{array}\right. } \end{aligned}$$

which is a solution of (5.73)-(5.74).

#### Lemma 5.10

The operator $\Psi _n$ in (5.75), defined for any $\omega \in \Omega _{n + 1}^\gamma$, satisfies, for any $s_0\leqslant s\leqslant S-\Sigma ({\mathtt b})$,

5.76 $$\begin{aligned} \begin{aligned}&|\Psi _n|_{0, s}^{\textrm{Lip}(\gamma )} \lesssim _{s} {\mathtt N}_{n}^{2 \tau + 1} \lambda ^{- 1} \gamma ^{- 1} |\textbf{E}_{n}|_{- M, s}^{\textrm{Lip}(\gamma )}, \\&\quad |\Psi _n|_{0, s + \eta }^{\textrm{Lip}(\gamma )} \lesssim _{s} {\mathtt N}_{n}^{2 \tau + 1 + \eta } \lambda ^{- 1}\gamma ^{- 1} |\textbf{E}_{n}|_{- M, s}^{\textrm{Lip}(\gamma )}, \quad \forall \, \eta > 0 . \end{aligned} \end{aligned}$$

Assume that $w_1,\, w_2$ satisfy (4.2) with $\sigma = \Sigma ({\mathtt b})$, as in (5.56). Then for any $\omega \in \Omega _{n + 1}^{\gamma _1}(w_1) \cap \Omega _{n + 1}^{\gamma _2}(w_2)$, $\gamma /2 \leqslant \gamma _1, \gamma _2 \leqslant \gamma$, one has

5.77 $$\begin{aligned} \begin{aligned} |\Delta _{12} \Psi _n|_{0, s_0}&\lesssim \varepsilon ^{M} \gamma ^{-M} {\mathtt N}_{n}^{2 \tau } {\mathtt N}_{n - 1}^{- {\mathtt a}} \Vert w_1 - w_2 \Vert _{s_1 + \Sigma ({\mathtt b})} , \\ |\Delta _{12} \Psi _n |_{0, s_0 + {\mathtt b}}&\lesssim \varepsilon ^{M} \gamma ^{-M} {\mathtt N}_{n}^{2 \tau } {\mathtt N}_{n - 1}\Vert w_1 - w_2 \Vert _{s_1 + \Sigma ({\mathtt b})} , \\ |\Delta _{12} \Psi _n|_{0, s_0 + \eta }&\lesssim \varepsilon ^{M} \gamma ^{-M} {\mathtt N}_{n}^{2 \tau + \eta } {\mathtt N}_{n - 1}^{- {\mathtt a}} \Vert w_1 - w_2 \Vert _{s_1 + \Sigma ({\mathtt b})} , \quad \forall \, \eta> 0 ,\\ |\Delta _{12} \Psi _n |_{0, s_0 + {\mathtt b}+ \eta }&\lesssim \varepsilon ^{M} \gamma ^{-M} {\mathtt N}_{n}^{2 \tau + \eta } {\mathtt N}_{n - 1}\Vert w_1 - w_2 \Vert _{s_1 + \Sigma ({\mathtt b})} , \quad \forall \, \eta > 0. \end{aligned} \end{aligned}$$

Moreover, $\Psi _n$ is real and momentum preserving. In addition, if we assume $w({\varphi },x)=\textrm{odd}({\varphi },x)$, the map $\Psi _n$ is also reversibility preserving.

#### Proof

To simplify notations, along this proof we drop the index *n*.

**Proof of** (5.76).

Let ${\widehat{\Psi }}(\ell )_{j}^{j'}={\widehat{\Psi }}(\ell ;\omega )_{j}^{j'}$ as in (5.75), with $\ell \in {\mathbb {Z}}^\nu$, $j, j' \in {\mathbb {Z}}^2 {\setminus } \{ 0 \}$, with $0 < |\ell | \leqslant {\mathtt N}$ and $\pi ^\top (\ell ) + j - j' = 0$. For any $\omega \in \Omega _{n + 1}^\gamma$ (see (5.61)), we immediately get the estimate

5.78 $$\begin{aligned} |{{\widehat{\Psi }}} (\ell ;\omega )_j^{j'}| \lesssim \lambda ^{- 1}\gamma ^{- 1} \langle \ell \rangle ^\tau | j' |^\tau |{\widehat{\textbf{E}}}(\ell ;\omega )_j^{j'}| \lesssim \lambda ^{- 1} \gamma ^{- 1} {\mathtt N}^\tau | j' |^\tau |{\widehat{\textbf{E}}}(\ell ;\omega )_j^{j'}| . \end{aligned}$$

We define $\delta _{\ell j j'}(\omega ):=\textrm{i}\,\lambda \, \omega \cdot \ell +\mu (j;\omega ) - \mu (j';\omega )$. Let $\omega _{1}, \omega _{2} \in \Omega _{n + 1}^\gamma$. By (5.60), (5.61), (5.62), we have

$$\begin{aligned} \begin{aligned}&\big | \big ( \mu (j;\omega _1) - \mu (j';\omega _1) \big ) - \big ( \mu (j;\omega _2) - \mu (j';\omega _2)\big ) \big | \\&\quad \leqslant \big |{\mathtt z}(j; \omega _1) - {\mathtt z}(j; \omega _2)\big | + \big |{\mathtt z}(j'; \omega _1) - {\mathtt z}(j'; \omega _2)\big | \\&\quad \lesssim \lambda ^{\theta } \gamma ^{- 1} \big ( |j|^{-1} + |j'|^{-1} \big ) |\omega _1 - \omega _2| \lesssim \lambda ^{\theta } \gamma ^{- 1} |\omega _1 - \omega _2| , \end{aligned} \end{aligned}$$

and therefore, using that $\lambda ^{\theta -1}\gamma ^{-1}\ll 1$ by (4.7),

5.79 $$\begin{aligned} \begin{aligned} |\delta _{\ell j j'}(\omega _1) - \delta _{\ell j j'}(\omega _2)|&\lesssim (\lambda \, |\ell |+\lambda ^{\theta }\gamma ^{-1}) |\omega _1 - \omega _2| \lesssim \lambda \, \langle \ell \rangle |\omega _1 - \omega _2|. \end{aligned} \end{aligned}$$

Then, estimate (5.79), together with the fact that $\omega _1, \omega _2 \in \Omega _{n + 1}^\gamma$, implies that

5.80 $$\begin{aligned} \begin{aligned} \Big | \frac{1}{ \delta _{\ell j j'}(\omega _{1})} - \frac{1}{\delta _{\ell j j'}(\omega _{2})} \Big |&\leqslant \dfrac{|\delta _{\ell j j'}(\omega _{1}) - \delta _{\ell j j'}(\omega _{2})|}{|\delta _{\ell j j'}(\omega _{1})| |\delta _{\ell j j'}(\omega _{2})|} \\&\lesssim \lambda (\lambda \gamma )^{-2} \langle \ell \rangle ^{2 \tau + 1} |j'|^{2 \tau } |\omega _{1} - \omega _{2} | \\&\lesssim \lambda ^{- 1} \gamma ^{- 2} \langle \ell \rangle ^{2 \tau + 1} |j'|^{2 \tau } |\omega _{1} - \omega _{2} |. \end{aligned} \end{aligned}$$

Therefore, by (5.75) and (5.80), for any $\omega _{1},\omega _{2}\in \Omega _{n + 1}^\gamma$ we have that

5.81 $$\begin{aligned} \begin{aligned} \big |{{\widehat{\Psi }}} (\ell ;\omega _{1})_j^{j'} - {{\widehat{\Psi }}} (\ell ;\omega _{2})_j^{j'}\big |&\lesssim \langle \ell \rangle ^\tau |j'|^\tau \lambda ^{- 1} \gamma ^{- 1} \big |{{\widehat{\textbf{E}}}}(\ell ;\omega _{1})_j^{j'} - {{\widehat{\textbf{E}}}}(\ell ;\omega _{2})_j^{j'}\big | \\&\ \ + \langle \ell \rangle ^{2 \tau + 1} |j'|^{2 \tau } \lambda ^{- 1} \gamma ^{-2} \big |{\widehat{\textbf{E}}}(\ell ;\omega _{2})_j^{j'}\big | |\omega _{1}-\omega _{2} | \\&\lesssim {\mathtt N}^\tau |j'|^\tau \lambda ^{- 1} \gamma ^{- 1} \big |{{\widehat{\textbf{E}}}}(\ell ;\omega _{1})_j^{j'} - {{\widehat{\textbf{E}}}}(\ell ;\omega _{2})_j^{j'}\big | \\&\ \ + {\mathtt N}^{2 \tau + 1} |j'|^{2 \tau } \lambda ^{- 1} \gamma ^{-2} \big |{\widehat{\textbf{E}}}(\ell ;\omega _{2})_j^{j'}\big | |\omega _{1}-\omega _{2} |. \end{aligned} \end{aligned}$$

Since $M > 2 \tau$ by (5.56), recalling Definition 2.5 and collecting the estimates (5.78), (5.81), we obtain the bounds, for any $s\in [s_0,S+\Sigma ({\mathtt b})]$,

$$\begin{aligned} \begin{aligned}&|\Psi |_{0, s}^\textrm{sup} \lesssim {\mathtt N}^{ \tau } \lambda ^{- 1}\gamma ^{- 1} |{\textbf{E}}|_{- M, s}^\textrm{sup}, \\&\quad |\Psi |_{0, s}^\textrm{lip} \lesssim {\mathtt N}^{2 \tau + 1} \lambda ^{- 1}\gamma ^{- 2} |{\textbf{E}}|_{- M, s}^\textrm{sup} + {\mathtt N}^{\tau } \lambda ^{- 1} \gamma ^{- 1} |{\textbf{E}}|_{- M, s}^\textrm{lip}. \end{aligned} \end{aligned}$$

Similarly, using also that $|\ell | \leqslant {\mathtt N}$ and $\pi ^\top (\ell ) + j - j' = 0$ imply that $|j - j'| \lesssim |\ell | \lesssim {\mathtt N}$, by analogous arguments we obtain that, for any $\eta > 0$,

$$\begin{aligned} \begin{aligned}&|\Psi |_{0, s + \eta }^\textrm{sup} \lesssim {\mathtt N}^{\tau + \eta } \gamma ^{- 1} \lambda ^{- 1} |{\textbf{E}}|_{- M, s}^\textrm{sup}, \\&\quad |\Psi |_{0, s+ \eta }^\textrm{lip} \lesssim {\mathtt N}^{2 \tau + \eta +1} \gamma ^{- 2} \lambda ^{- 1} |{\textbf{E}}|_{- M, s}^\textrm{sup} + {\mathtt N}^{\tau + \eta } \lambda ^{- 1} \gamma ^{- 2} |{\textbf{E}}|_{- M, s}^\textrm{sup}. \end{aligned} \end{aligned}$$

Hence, we conclude the claimed bounds in (5.76).

**Proof of** (5.77). Let $\ell \in {\mathbb {Z}}^{\nu }$, $j, j' \in {\mathbb {Z}}^2 {\setminus } \{ 0\}$, with $0 < |\ell | \leqslant {\mathtt N}$ and $\pi ^\top (\ell ) + j - j' = 0$. Recalling (5.60), we set

$$\begin{aligned} \begin{aligned}&\delta _{\ell j j'}(w_i):= \textrm{i}\, \lambda \,\omega \cdot \ell + \mu (j; w_i) - \mu (j'; w_i) , \quad i = 1,2, \\&\quad \mu (j; w_i):= \textrm{i}\, \beta \,{\mathtt L}(j)+ {\mathtt z}(j; w_i). \end{aligned} \end{aligned}$$

By (5.70), we get that

$$\begin{aligned} \begin{aligned} |\Delta _{12} \delta _{\ell j j'}|&\leqslant |\Delta _{12} {\mathtt z}(j)| + |\Delta _{12} {\mathtt z}(j')| \lesssim \lambda ^{\theta }\big (|j|^{- 1} + |j'|^{- 1} \big ) \Vert w_1 - w_2 \Vert _{s_1 + \Sigma ({\mathtt b})} \\&\lesssim \lambda ^{\theta } \Vert w_1 - w_2 \Vert _{s_1 + \Sigma ({\mathtt b})} . \end{aligned} \end{aligned}$$

Therefore, by (5.75), (5.96) and using that $\lambda ^{\theta -1}\gamma ^{-1}\ll 1$, we get, for any $\omega \in \Omega _{n + 1}^{\gamma _1}(w_1) \cap \Omega _{n + 1}^{\gamma _1}(w_2)$ with $\gamma /2 \leqslant \gamma _1, \gamma _2 \leqslant \gamma$,

$$\begin{aligned} \begin{aligned} \big |\Delta _{12} {\widehat{\Psi }}(\ell )_j^{j'}\big |&\leqslant \frac{\big |\Delta _{12} {{\widehat{\textbf{E}}}}(\ell )_j^{j'}\big |}{|\delta _{\ell j j'}(w_1)|} + \frac{\big |{{\widehat{\textbf{E}}}}(\ell )_j^{j'}(w_2\big )| |\Delta _{12} \delta _{\ell j j'}|}{|\delta _{\ell j j'}(w_1)| |\delta _{\ell j j'}(w_2)|} \\&\lesssim {\mathtt N}^\tau \lambda ^{- 1} \gamma ^{- 1} |j'|^\tau \big |\Delta _{12} {{\widehat{\textbf{E}}}}(\ell )_j^{j'}\big | \\&\quad + {\mathtt N}^{2 \tau } \lambda ^{\theta } \lambda ^{- 2} \gamma ^{- 2} |j'|^{2 \tau }\big |{{\widehat{\textbf{E}}}}(\ell )_j^{j'}(w_2)\big | \Vert w_1 - w_2 \Vert _{s_1 + \Sigma ({\mathtt b})} \\&\lesssim {\mathtt N}^{2 \tau } \lambda ^{- 1} \gamma ^{- 1} |j'|^{2 \tau } \big ( \big |\Delta _{12} {{\widehat{\textbf{E}}}}(\ell )_j^{j'}\big | + \big |{{\widehat{\textbf{E}}}}(\ell )_j^{j'}(w_2)\big | \Vert w_1 - w_2 \Vert _{s_1 + \Sigma ({\mathtt b})}\big ) . \end{aligned} \end{aligned}$$

Since $M > 2 \tau$ by (5.56), recalling Definition 2.5 and the induction estimates (5.64), (5.69), the claimed bounds follow easily. Finally, since ${\textbf{E}}$ is real, reversible and momentum preserving, by (5.75), Lemma 2.9 and Lemma 2.13 we have that $\Psi$ is real, reversibility preserving and momentum preserving. This concludes the proof. $\square$

By Lemma 5.10, the induction assumption on the estimates (5.64) and by (5.58), we obtain, for any $s_0\leqslant s\leqslant S-\Sigma ({\mathtt b})$,

5.82 $$\begin{aligned} \begin{aligned} |\Psi _n|_{0, s}^{\textrm{Lip}(\gamma )}&\lesssim _{s} {\mathtt N}_n^{2 \tau + 1} \lambda ^{- 1}\gamma ^{- 1} |{\textbf{E}}_n |_{- M, s}^{\textrm{Lip}(\gamma )} \lesssim _{s } {\mathtt N}_n^{2 \tau + 1} {\mathtt N}_{n - 1}^{- {\mathtt a}} \varepsilon ^M \gamma ^{- M} \Vert w \Vert _{s + \Sigma ({\mathtt b})}^{\textrm{Lip}(\gamma )} , \\ |\Psi _n|_{0, s + {\mathtt b}}^{\textrm{Lip}(\gamma )}&\lesssim _{s} {\mathtt N}_n^{2 \tau + 1} \lambda ^{- 1} \gamma ^{- 1} |{\textbf{E}}_n |_{-M, s + {\mathtt b}}^{\textrm{Lip}(\gamma )} \lesssim _{s } {\mathtt N}_n^{2 \tau + 1} {\mathtt N}_{n - 1} \varepsilon ^{M}\gamma ^{- M} \Vert w \Vert _{s + \Sigma ({\mathtt b})}^{\textrm{Lip}(\gamma )}, \end{aligned} \end{aligned}$$

which are the estimates (5.66) at the step $n + 1$. Moreover, setting $\eta =M$ in (5.76), by the same arguments we also have

5.83 $$\begin{aligned} \begin{aligned} |\Psi _n|_{0, s + M}^{\textrm{Lip}(\gamma )} ,\,&\lesssim _{s} {\mathtt N}_n^{2 \tau + 1 + M} \gamma ^{- 1} \lambda ^{- 1} |{\textbf{E}}_n |_{- M, s}^{\textrm{Lip}(\gamma )} \\&\lesssim _{s } {\mathtt N}_n^{2 \tau + 1 + M} {\mathtt N}_{n - 1}^{- {\mathtt a}} \varepsilon ^M \gamma ^{- M} \Vert w \Vert _{s + \Sigma ({\mathtt b})}^{\textrm{Lip}(\gamma )} , \\ |\Psi _n|_{0, s + {\mathtt b}+ M}^{\textrm{Lip}(\gamma )}&\lesssim _{s} {\mathtt N}_n^{2 \tau + 1 + M} \lambda ^{- 1}\gamma ^{- 1} |{\textbf{E}}_n |_{-M, s + {\mathtt b}}^{\textrm{Lip}(\gamma )} \\&\lesssim _{s } {\mathtt N}_n^{2 \tau + M + 1} {\mathtt N}_{n - 1} \varepsilon ^M \gamma ^{- M} \Vert w \Vert _{s + \Sigma ({\mathtt b})}^{\textrm{Lip}(\gamma )} . \end{aligned} \end{aligned}$$

In particular, by (5.56), (4.2) and by the smallness condition (5.59), we deduce, setting $s=s_0$ in (5.83), for ${\mathtt N}_0>0$ large enough,

5.84 $$\begin{aligned} \begin{aligned} |\Psi _n|_{0, s_0 + M}^{\textrm{Lip}(\gamma )}&\lesssim {\mathtt N}_n^{2 \tau +1 + M} {\mathtt N}_{n - 1}^{- {\mathtt a}} \varepsilon ^M \gamma ^{- M} \Vert w \Vert _{s_0 + \Sigma ({\mathtt b})}^{\textrm{Lip}(\gamma )} \ \leqslant \delta < 1. \end{aligned} \end{aligned}$$

Therefore, by Lemma 2.6-(*iv*) and estimates (5.83), (5.84), we have the estimates, for any $s_0 \leqslant s \leqslant S - \Sigma ({\mathtt b})$,

5.85 $$\begin{aligned} \begin{aligned}&\sup _{\tau \in [- 1, 1]}|\textrm{exp}(\tau \Psi _n)|_{0, s + M}^{\textrm{Lip}(\gamma )} \lesssim _s 1 + |\Psi _n|_{0, s + M}^{\textrm{Lip}(\gamma )}, \quad \sup _{\tau \in [- 1, 1]}|\textrm{exp}(\tau \Psi _n)|_{0, s_0 + M}^{\textrm{Lip}(\gamma )} \lesssim 1, \\&\quad \sup _{\tau \in [- 1, 1]}|\textrm{exp}(\tau \Psi _n)|_{0, s + {\mathtt b}+ M}^{\textrm{Lip}(\gamma )} \lesssim _s 1 + |\Psi _n|_{0, s + {\mathtt b}+ M}^{\textrm{Lip}(\gamma )}. \end{aligned} \end{aligned}$$

Then, by recalling (5.72) and by using that $\Psi _n$ solves the equation (5.73), we conclude that

5.86 $$\begin{aligned} \begin{aligned} {\textbf{L}}_{n + 1}&\,=\lambda \, \omega \cdot \partial _\varphi + {\textbf{D}}_{n + 1} + {\textbf{E}}_{n + 1}, \\ {\textbf{D}}_{n + 1}&:= -\beta {\mathtt L}+ {\textbf{Z}}_{n + 1}, \qquad {\textbf{Z}}_{n + 1}: = {\textbf{Z}}_n + {\widehat{\textbf{E}}}_n(0), \\ {\textbf{E}}_{n + 1}&\,=\Pi _{{\mathtt N}_n}^\bot \textbf{E}_n + \int _0^1 (1 - \tau ) \textrm{exp}(- \tau \Psi _n) \,[{\widehat{\textbf{E}}}_n(0) - \Pi _{{\mathtt N}_n} \textbf{E}_n, \Psi _n] \, \textrm{exp}(\tau \Psi _n)\,\textrm{d}{\tau }\\&\quad + \int _0^1 \textrm{exp}(- \tau \Psi _n) [\textbf{E}_n, \Psi _n] \textrm{exp}(\tau \Psi _n) \,\textrm{d}{\tau }. \end{aligned}\nonumber \\ \end{aligned}$$

All the operators in (5.86)

are defined for any $\omega \in \Omega _{n + 1}^\gamma$. Moreover, by (5.72), (5.73), for $\omega \in \Omega ^\gamma _{n+1}$ one has the identity $\Phi _n^{-1} \textbf{L}_n \Phi _n = \textbf{L}_{n+1}$, which is (5.65) at the step $n+1$. Recalling that ${\widehat{\textbf{E}}}_{n}(0)$ is a diagonal operator and by (5.86), we have that

5.87 $$\begin{aligned} \begin{aligned} {\textbf{Z}}_{n + 1}&= \textrm{diag}_{j \in {\mathbb {Z}}^2 \setminus \{ 0 \}} {\mathtt z}_{n + 1}(j), \quad {\mathtt z}_{n + 1}(j):= {\mathtt z}_n(j) + {\widehat{\textbf{E}}}_n(0)_j^j, \\ {\textbf{D}}_{n + 1}&= \textrm{diag}_{j \in {\mathbb {Z}}^2 \setminus \{ 0 \}} \mu _{n + 1}(j), \quad \mu _{n + 1}(j):= \textrm{i}\, \beta \, {\mathtt L}(j)+ {\mathtt z}_{n + 1}(j). \end{aligned} \end{aligned}$$

By Lemma 2.6-(*v*)

5.88 $$\begin{aligned} \begin{aligned} | \mu _{n + 1}(j) - \mu _n(j)|^{\textrm{Lip}(\gamma )}&= | {\mathtt z}_{n + 1}(j) - {\mathtt z}_n(j)|^{\textrm{Lip}(\gamma )} \\&\leqslant | {\widehat{\textbf{E}}}_n(0)_j^j |^{\textrm{Lip}(\gamma )} \lesssim |{\textbf{E}}_n|_{- M, s_0}^{\textrm{Lip}(\gamma )} \langle j \rangle ^{- M}. \end{aligned} \end{aligned}$$

Then, (5.88), together with the estimate (5.64),

implies (5.63) at the step $n + 1$. The estimate (5.62) at the step $n + 1$ follows, as usual, by a telescoping argument, using the fact that $\sum _{n \geqslant 0} {\mathtt N}_{n - 1}^{- {\mathtt a}}$ is convergent since ${\mathtt a}> 0$ (see (5.56)). Now we prove the estimates (5.64) at the step $n + 1$. By (5.86), estimates (5.82), (5.84), (5.85), the induction estimates (5.64), Lemma 2.6-(*ii*), (*v*) and Lemma 2.7, we get, for any $s_0\leqslant s \leqslant S-\Sigma ({\mathtt b})$,

5.89 $$\begin{aligned} \begin{aligned}&|{\textbf{E}}_{n + 1}|_{- M, s}^{\textrm{Lip}(\gamma )} \lesssim _{s} {\mathtt N}_n^{- {\mathtt b}} |{\textbf{E}}_n|_{- M, s + {\mathtt b}}^{\textrm{Lip}(\gamma )} + {\mathtt N}_n^{2 \tau + 1 + M}\lambda ^{- 1} \gamma ^{- 1} |{\textbf{E}}_n|_{- M, s_0}^{\textrm{Lip}(\gamma )} |{\textbf{E}}_n|_{- M, s}^{\textrm{Lip}(\gamma )} , \\&\quad |{\textbf{E}}_{n + 1}|_{- M, s + {\mathtt b}}^{\textrm{Lip}(\gamma )} \lesssim _{s} |{\textbf{E}}_n|_{- M, s +{\mathtt b}}^{\textrm{Lip}(\gamma )} \\&\qquad + {\mathtt N}_{n}^{2 \tau + 1 + M} \lambda ^{- 1}\gamma ^{-1}\big ( |{\textbf{E}}_n|_{- M, s_0}^{\textrm{Lip}(\gamma )} |{\textbf{E}}_n|_{- M, s+{\mathtt b}}^{\textrm{Lip}(\gamma )} + |{\textbf{E}}_n|_{- M, s}^{\textrm{Lip}(\gamma )} |{\textbf{E}}_n|_{- M, s_0+{\mathtt b}}^{\textrm{Lip}(\gamma )} \big ) . \end{aligned} \end{aligned}$$

We now verify the estimates (5.64) at the step $n + 1$. By (5.89), the induction assumption on the estimate (5.64), (5.58) and using the ansatz (4.2) with $\sigma = \Sigma ({\mathtt b})$, we get, for any $s_0 \leqslant s \leqslant S - \Sigma ({\mathtt b})$,

5.90 $$\begin{aligned} \begin{aligned} |{\textbf{E}}_{n + 1}|_{- M, s +{\mathtt b}}^{\textrm{Lip}(\gamma )}&\leqslant C(s) C_*(s){\mathtt N}_{n - 1} \varepsilon _{M} \gamma ^{-(M-1)} \Vert w\Vert _{s+\Sigma ({\mathtt b})}^{\textrm{Lip}(\gamma )} \\&+ 2C(s)C_*^2(s) C_0 \,{\mathtt N}_n^{2 \tau + M + 1} {\mathtt N}_{n - 1}^{- {\mathtt a}}\varepsilon ^{M} \gamma ^{- M} \varepsilon _{M} \gamma ^{-(M-1)} \Vert w \Vert _{s + \Sigma ({\mathtt b})}^{\textrm{Lip}(\gamma )} \\&\leqslant C_*(s) {\mathtt N}_n \varepsilon _{M} \gamma ^{-(M-1)} \Vert w \Vert _{s + \Sigma ({\mathtt b})}^{\textrm{Lip}(\gamma )} , \end{aligned}\nonumber \\ \end{aligned}$$

provided

5.91 $$\begin{aligned} C(s) {\mathtt N}_{n}^{-1} {\mathtt N}_{n - 1} \leqslant \frac{1}{2} , \quad 2 C(s)C_*(s) C_0\, {\mathtt N}_n^{2 \tau + M } {\mathtt N}_{n - 1}^{ - {\mathtt a}}\varepsilon ^{M} \gamma ^{- M} \leqslant \frac{1}{2}, \end{aligned}$$

and

5.92 $$\begin{aligned} \begin{aligned} |{\textbf{E}}_{n + 1}|_{- M, s}^{\textrm{Lip}(\gamma )}&\leqslant C(s) C_*(s) {\mathtt N}_n^{- {\mathtt b}} {\mathtt N}_{n - 1} \varepsilon _{M} \gamma ^{-(M-1)} \Vert w \Vert _{s+ \Sigma ({\mathtt b})}^{\textrm{Lip}(\gamma )} \\&+ C(s) C_*^2(s) C_0\, {\mathtt N}_n^{2 \tau + M + 1} {\mathtt N}_{n - 1}^{- 2 {\mathtt a}} \varepsilon ^{-M} \gamma ^{-M} \varepsilon _{M} \gamma ^{-(M-1)} \Vert w \Vert _{s+ \Sigma ({\mathtt b})}^{\textrm{Lip}(\gamma )} \\&\leqslant C_*(s) {\mathtt N}_n^{- {\mathtt a}} \varepsilon _{M} \gamma ^{-(M-1)} \Vert w \Vert _{s + \Sigma ({\mathtt b})}^{\textrm{Lip}(\gamma )} , \end{aligned} \end{aligned}$$

provided

5.93 $$\begin{aligned} C(s) {\mathtt N}_n^{{\mathtt a}- {\mathtt b}} {\mathtt N}_{n - 1} \leqslant \frac{1}{2} , \quad C(s) C_*(s)C_0 \, {\mathtt N}_n^{2 \tau + {\mathtt a}+ M + 1} {\mathtt N}_{n - 1}^{- 2 {\mathtt a}}\varepsilon ^{M} \gamma ^{- M} \leqslant \frac{1}{2} ; \end{aligned}$$

note that the constant *C*(*s*) comes from estimates (5.89), the constant $C_*(s)$ comes from the estimates (5.64) and the constant $C_0$ comes from impositing the ansatz (4.2). The conditions (5.91), (5.93) are verified by (5.56), (5.59), taking ${\mathtt N}_0, \lambda \gg 0$ large enough. Therefore, the estimates (5.90) and (5.92) imply that the estimates (5.64) hold at the step $n + 1$. Finally, since $\Psi _n, \Phi _n, \Phi _n^{- 1}$ are real and momentum preserving by Lemma 5.73 and ${\textbf{D}}_n, {\textbf{E}}_n$ are real and momentum preserving by the induction assumption, then ${\textbf{D}}_{n + 1}$, ${\textbf{E}}_{n + 1}$ are real and momentum preserving operators, by (5.86) and Lemmata 2.9, 2.11, 2.13. Morover if $w = \textrm{odd}({\varphi }, x)$, since $\Psi _n, \Phi _n, \Phi _n^{- 1}$ are reversibility preserving by Lemma 5.73 and ${\textbf{D}}_n, {\textbf{E}}_n$ are reversible by the induction assumption, then ${\textbf{D}}_{n + 1}$, ${\textbf{E}}_{n + 1}$ are reversible by (5.86) and Lemma 2.9 and hence (5.67) is verified at the step $n + 1$.

Proof of $\mathbf{(S2)}_{n + 1}$. We now construct a Lipschitz extension for the eigenvalues $\mu _{n + 1}(j,\,\cdot \,): \Omega _{n + 1}^\gamma \rightarrow \,{\mathbb {C}}$. By the induction hypothesis, there exists a Lipschitz extension of $\mu _n(j;\omega )$, denoted by ${{\widetilde{\mu }}}_{n}(j;\omega )$, to the whole set ${\mathtt D}{\mathtt C}(2\gamma , \tau )$ that satisfies $\mathbf{(S2)}_n$. By (5.87), we have $\mu _{n + 1}(j) = \mu _n(j) + {\mathtt r}_n(j)$, where ${\mathtt r}_n(j, \omega )={\mathtt r}_n(j,\omega ):= {\widehat{\textbf{E}}}_n(0;\omega )_j^j$ satisfies $|{\mathtt r}_n(j)|^{\textrm{Lip}(\gamma )} \lesssim {\mathtt N}_{n - 1}^{- {\mathtt a}} |j|^{- M}$.

Hence, by the Kirszbraun Theorem (see Lemma M.5 [39]) there exists a Lipschitz extension ${{\widetilde{{\mathtt r}}}}_n(j,\,\cdot \,): {{\mathtt D}{\mathtt C}(2\gamma , \tau ) \cap \Lambda _{o}}\rightarrow {\mathbb {C}}$ of ${\mathtt r}_n(j,\,\cdot \,): \Omega _{n + 1}^\gamma \rightarrow {\mathbb {R}}$ satisfying $|{{\widetilde{{\mathtt r}}}}_n(j)|^{\textrm{Lip}(\gamma )} \lesssim |{\mathtt r}_n(j)|^{\textrm{Lip}(\gamma )} \lesssim {\mathtt N}_{n - 1}^{- {\mathtt a}} |j|^{- M}$. The claimed statement then follows by defining ${{\widetilde{\mu }}}_{n + 1}(j):= {{\widetilde{\mu }}}_n(j) + {{\widetilde{{\mathtt r}}}}_n(j)$.

Proof of $\mathbf{(S3)}_{n + 1}$. Let $w_1, w_2$ satisfy the ansatz (4.2) with $\sigma = \Sigma ({\mathtt b})$ and let $\omega \in \Omega _{n + 1}^{\gamma _1}(w_1) \cap \Omega _{n + 1}^{\gamma _2}(w_2)$, $\gamma _1, \gamma _2 \in [\gamma /2\,,\, 2 \gamma ]$. Let

$$\begin{aligned} \Delta _{12}{{\mathcal {F}}}_\tau := {{\mathcal {F}}}_\tau (w_1) - {\mathcal F}_\tau (w_2) , \quad {{\mathcal {F}}}_\tau := \textrm{exp}(\tau \Psi _n), \quad \tau \in [-1, 1]. \end{aligned}$$

By Lemmata 2.6-(*iv*), 5.10 with $s_1=s_0$, together with $\Delta _{12} {\mathcal {F}}_{\tau } = (\textrm{exp } (\tau \Delta _{12} \Psi _{n} )-\textrm{Id}) {\mathcal {F}}_{\tau } (w_2)$, we have

5.94 $$\begin{aligned} \begin{aligned}&|\Delta _{12} {{\mathcal {F}}}_\tau |_{0, s_0} \lesssim {\mathtt N}_{n}^{2 \tau } {\mathtt N}_{n - 1}^{- {\mathtt a}}\varepsilon ^{M} \gamma ^{-M} \Vert w_1 - w_2 \Vert _{s_0 + \Sigma ({\mathtt b})}, \\&\quad |\Delta _{12} {{\mathcal {F}}}_\tau |_{0, s_0 + M} \lesssim {\mathtt N}_{n}^{2 \tau + M} {\mathtt N}_{n - 1}^{- {\mathtt a}} \varepsilon ^{M} \gamma ^{-M}\Vert w_1 - w_2 \Vert _{s_0 + \Sigma ({\mathtt b})}, \\&\quad |\Delta _{12} {{\mathcal {F}}}_\tau |_{0, s_0 + \beta } \lesssim {\mathtt N}_{n}^{2 \tau } {\mathtt N}_{n - 1} \varepsilon ^{M} \gamma ^{-M}\Vert w_1 - w_2 \Vert _{s_0 + \Sigma ({\mathtt b})} , \\&\quad |\Delta _{12} {{\mathcal {F}}}_\tau |_{0, s_0 + M + \beta } \lesssim {\mathtt N}_{n}^{2 \tau + M} {\mathtt N}_{n - 1} \varepsilon ^{M} \gamma ^{-M}\Vert w_1 - w_2 \Vert _{s_0 + \Sigma ({\mathtt b})}. \end{aligned} \end{aligned}$$

We now shortly describe how to estimate $\Delta _{12} \textbf{E}_{n + 1}$ (recall the expression given in (5.86)). By Lemma 2.7 and the estimates in (5.69), we have that

5.95 $$\begin{aligned} \begin{aligned} |\Delta _{12} \Pi _{{\mathtt N}_n}^\bot \textbf{E}_n|_{- M, s_0}&\lesssim {\mathtt N}_n^{- {\mathtt b}} |\Delta _{12} \textbf{E}_n|_{- M, s_0 + {\mathtt b}} \lesssim {\mathtt N}_n^{- {\mathtt b}} {\mathtt N}_{n - 1} \Vert w_1 - w_2 \Vert _{s_0 + \Sigma ({\mathtt b})}, \\ |\Delta _{12} \Pi _{{\mathtt N}_n}^\bot \textbf{E}_n|_{- M, s_0+ {\mathtt b}}&\leqslant |\Delta _{12} \textbf{E}_n|_{- M, s_0+ {\mathtt b}} \lesssim {\mathtt N}_{n - 1} \Vert w_1 - w_2\Vert _{s_0 + \Sigma ({\mathtt b})}. \end{aligned} \end{aligned}$$

All the terms appearing in the two integrals in the formula of $\textbf{E}_{n + 1}$ (see (5.86)) can be all estimated in a similar way. Hence, for sake of simplicity in the exposition, we concentrate on the estimate of the term

5.96 $$\begin{aligned} \textbf{B}:= \int _0^1 \textbf{V}_{\tau } \,\textrm{d}{\tau }, \quad \textbf{V}_{\tau }:= {{\mathcal {F}}}_{- \tau } \textbf{E}_n \Psi _n {{\mathcal {F}}}_\tau . \end{aligned}$$

Clearly it is enough to estimate $\textbf{V}_{\tau }$ uniformly with respect to $\tau \in [0, 1]$. By applying the estimates (5.64), (5.69), (5.77), (5.82), (5.85), (5.94) (using also that, by the ansatz (4.2), $\Vert w_i \Vert _{s_0 + \Sigma ({\mathtt b})}\leqslant 1$, $i = 1,2$) plus a repeated use of the triangular inequality and Lemma 2.6-(*ii*), one can show that $\Delta _{12} \textbf{B}$ satisfies the estimates

5.97 $$\begin{aligned} \begin{aligned}&|\Delta _{12} \textbf{B}|_{- M, s_0} \lesssim {\mathtt N}_n^{2 \tau + 1 + M} {\mathtt N}_{n - 1}^{- 2 {\mathtt a}} \varepsilon ^{M} \gamma ^{-M} \varepsilon _{M} \gamma ^{- (M-1)} \Vert w_1 - w_2 \Vert _{s_0 + \Sigma ({\mathtt b})}, \\&\quad |\Delta _{12} \textbf{B}|_{- M, s_0 + {\mathtt b}} \lesssim {\mathtt N}_n^{2 \tau + 1 + M} {\mathtt N}_{n - 1}^{1 - {\mathtt a}} \varepsilon ^{M} \gamma ^{-M} \varepsilon _{M} \gamma ^{- (M-1)} \Vert w_1 - w_2 \Vert _{s_0 + \Sigma ({\mathtt b})} . \end{aligned} \end{aligned}$$

Collecting the estimates (5.95), (5.97), we obtain that

$$\begin{aligned} \begin{aligned}&| \Delta _{12} \textbf{E}_{n + 1} |_{- M, s_0} \lesssim \Big ( {\mathtt N}_n^{- {\mathtt b}} {\mathtt N}_{n - 1} + {\mathtt N}_n^{2 \tau + 1 + M} {\mathtt N}_{n - 1}^{- 2 {\mathtt a}} \varepsilon ^{M} \gamma ^{- M}\Big ) \varepsilon _{M} \gamma ^{-(M-1)} \Vert w_1 - w_2 \Vert _{s_0 + \Sigma ({\mathtt b})}, \\&\quad | \Delta _{12} \textbf{E}_{n + 1} |_{- M, s_0 + {\mathtt b}} \lesssim \Big ( {\mathtt N}_{n - 1} + {\mathtt N}_n^{2 \tau + 1 + M} {\mathtt N}_{n - 1}^{1 - {\mathtt a}} \varepsilon ^{M} \gamma ^{-M} \ \Big ) \varepsilon _{M} \gamma ^{-(M-1)} \Vert w_1 - w_2 \Vert _{s_0 + \Sigma ({\mathtt b})}, \end{aligned} \end{aligned}$$

which imply the claimed bounds (5.69) at the step $n + 1$, arguing as in (5.90)-(5.93).

We now verify the estimates (5.70) at the step $n + 1$. Recalling (5.87) and by Lemma 2.6-(*v*), together with the estimate (5.69), we have, for any $j \in {\mathbb {Z}}^2 {\setminus } \{ 0 \}$,

$$\begin{aligned} \begin{aligned} |\Delta _{12}({\mathtt z}_{n + 1}(j) - {\mathtt z}_n(j))|&= |\Delta _{12} {\widehat{\textbf{E}}}_n(0)_j^j| \lesssim |j|^{- M} |\Delta _{12} \textbf{E}_n|_{- M, s_0} \\&\lesssim |j|^{- M} {\mathtt N}_{n - 1}^{- {\mathtt a}} \varepsilon _{M} \gamma ^{-(M-1)} \Vert w_1 - w_2 \Vert _{s_0 + \Sigma ({\mathtt b})} , \end{aligned} \end{aligned}$$

which is the first estimate in (5.70) at the step $n + 1$. Moreover, using that ${\mathtt z}_{n + 1}(j) = {\mathtt z}_0(j) + \sum _{k = 0}^n {\mathtt z}_{k + 1} - {\mathtt z}_k$ and recalling also that $|\Delta _{12} {\mathtt z}_0| \lesssim \lambda ^{\theta } |j|^{- 1} \Vert w_1 - w_2 \Vert _{s_0 + \Sigma ({\mathtt b})}$, we have,

$$\begin{aligned} \begin{aligned} |\Delta _{12} {\mathtt z}_{n + 1}(j)|&\leqslant | \Delta _{12}{\mathtt z}_0(j)| + \sum _{k = 0}^n | \Delta _{12}({\mathtt z}_{k + 1} - {\mathtt z}_k) | \\&\lesssim \Big ( \lambda ^{\theta } |j|^{- 1} + |j|^{- M} \sum _{k = 0}^n {\mathtt N}_{k - 1}^{- {\mathtt a}} \varepsilon _{M} \gamma ^{-(M-1)} \Big ) \Vert w_1 - w_2 \Vert _{s_0 + \Sigma ({\mathtt b})} \\&\lesssim \lambda ^{\theta } |j|^{- 1} \Vert w_1 - w_2 \Vert _{s_0 + \Sigma ({\mathtt b})} , \end{aligned} \end{aligned}$$

by using (5.58), the fact that $M > 1$, and that the series $\sum _{k \geqslant 0} {\mathtt N}_{k - 1}^{- {\mathtt a}} < \infty$ is convergent and $\varepsilon \gamma ^{-1}\ll 1$. Hence, the second estimate in (5.70) at the step $n + 1$ is proved and the proof of $\mathbf{(S3)}_{n + 1}$ is completed.

Proof of $\mathbf{(S4)}_{n + 1}$. Let $\omega \in \Omega _{n+1}^{\gamma }(w_1)$. By (5.61) and the induction hypothesis (5.71) (i.e. $\mathbf{(S4)}_n$), we have that $\Omega _{n+1}^{\gamma }(w_1) \subseteq \Omega _{n}^{\gamma }(w_1) \subseteq \Omega _{n}^{\gamma - \rho }(w_2)$. Moreover, $\omega \in \Omega _n^{\gamma - \rho }(w_2) \subseteq \Omega _n^{\gamma /2}(w_2)$ because $\rho \leqslant \gamma /2$. Thus $\Omega _{n + 1}^\gamma (w_1) \subseteq \Omega _n^{\gamma - \rho }(w_2) \subseteq \Omega _n^{\gamma /2}(w_2)$. We also have that (5.60) holds for any $\omega \in \mathtt \Omega _{n+1}^{\gamma }(w_1)$, as well estimate (5.70) for any $j \in {\mathbb {Z}}^2 \setminus \{ 0 \}$, namely, for any $\omega \in \Omega _{n + 1}^\gamma (w_1)$

5.98 $$\begin{aligned} |\Delta _{12} \mu _n(j; \omega )| = |\Delta _{12} {\mathtt z}_n(j; \omega )| \lesssim \lambda ^{\theta } |j|^{- 1} \Vert w_1 - w_2 \Vert _{ s_0 + \Sigma ({\mathtt b})}^\textrm{sup}. \end{aligned}$$

Let $\ell \in {\mathbb {Z}}^{\nu }\setminus \{0\}$ and $j,j'\in {\mathbb {Z}}^2\setminus \{0\}$, with $|\ell |\leqslant {\mathtt N}_{n}$ and $\pi ^\top (\ell )+j-j' =0$. We distinguish two regimes:

5.99 $$\begin{aligned} \textrm{min}\{ |j|, |j'| \} \geqslant {\mathtt N}_n^\tau \quad \text {and} \quad \textrm{min}\{ |j|, |j'| \} \leqslant {\mathtt N}_n^\tau . \end{aligned}$$

We then organize the rest of the proof in two claims.

**Claim 1.** Let $\omega \in \Omega _{n+1}^{\gamma }(w_1)$. If $\min \{|j|, |j'|\} \geqslant {\mathtt N}_{n}^\tau$, having $\lambda ^{\theta -1} \gamma ^{- 1} \ll 1$ small enough, then

$$\begin{aligned} |\textrm{i}\,\lambda \, \omega \cdot \ell + \mu _n(j;w_2) - \mu _n(j'; w_2)| \geqslant \frac{\lambda (\gamma - \rho )}{\langle \ell \rangle ^\tau |j'|^\tau }. \end{aligned}$$

**Proof of the Claim 1.** By (5.60) and the estimates (5.62), we have, for any $j\in {\mathbb {Z}}^2 {\setminus }\{0\}$, that $|\mu _n(j; w_2)| \leqslant C \lambda ^{\theta } |j|^{- 1}$ for some constant $C > 0$. Therefore, using that $\textrm{min}\{ |j|, |j'| \} \geqslant {\mathtt N}_n^\tau$ and recalling that $\omega \in {\mathtt D} {\mathtt C}(2 \gamma , \tau )$, we obtain

5.100 $$\begin{aligned} \begin{aligned} |\textrm{i}\, \lambda \, \omega \cdot \ell + \mu _n(j; w_2) - \mu _n(j';w_2)|&\geqslant \lambda |\omega \cdot \ell | - C \lambda ^{\theta } \big ( |j|^{- 1} + |j'|^{- 1} \big ) \\&\geqslant \frac{2 \lambda \, \gamma }{\langle \ell \rangle ^\tau } - \frac{2 C \lambda ^{\theta }}{\textrm{min}\{ |j|, |j'| \}} \\&\geqslant \frac{2\lambda \,\gamma }{\langle \ell \rangle ^\tau } - \frac{2 C \lambda ^{\theta }}{{\mathtt N}_n^\tau } \geqslant \frac{\lambda \,\gamma }{\langle \ell \rangle ^\tau } , \end{aligned} \end{aligned}$$

provided that

$$\begin{aligned} 2C \lambda ^{\theta -1} \gamma ^{- 1} \langle \ell \rangle ^\tau {\mathtt N}_n^{- \tau } \leqslant 1 , \quad \forall \, \ell \in {\mathbb {Z}}^\nu , \quad 0 < |\ell | \leqslant {\mathtt N}_n. \end{aligned}$$

The latter condition is verified by taking $\lambda ^{\theta -1} \gamma ^{- 1} \ll 1$ small enough, by (4.7). The claimed statement then follows directly by (5.100) since $\gamma \geqslant \gamma - \rho$ and $\langle \ell \rangle ^\tau \leqslant \langle \ell \rangle ^\tau |j'|^\tau$.

We now analyze the other regime in (5.99), namely when $\textrm{min} \{ |j|, |j'| \} \leqslant {\mathtt N}_n^\tau$.

**Claim 2.** Let $\omega \in \Omega _{n+1}^{\gamma }(w_1)$. If $\min \{|j|, |j'|\} \leqslant {\mathtt N}_{n}^\tau$ and ${\mathtt N}_n^{\tau + \tau ^2} \lambda ^{\theta -1} \rho ^{- 1} \Vert w_1 - w_2 \Vert _{s_0 + \Sigma ({\mathtt b})}\ll 1$ is small enough, then

$$\begin{aligned} |\textrm{i}\,\lambda \, \omega \cdot \ell + \mu _n(j; w_2) - \mu _n(j'; w_2)| \geqslant \frac{\gamma - \rho }{\langle \ell \rangle ^\tau |j'|^\tau }. \end{aligned}$$

**Proof of the Claim 2.** By (5.98), and using that $\omega \in \Omega _{n+1}^{\gamma }(w_1)$ and $\min \{|j|, |j'|\} \leqslant {\mathtt N}_{n}^\tau$, we have that

5.101 $$\begin{aligned} \begin{aligned}&|\textrm{i}\, \lambda \, \omega \cdot \ell + \mu _n(j; w_2) - \mu _n(j'; w_2)| \geqslant |\textrm{i}\,\lambda \, \omega \cdot \ell + \mu _n(j; w_1) - \mu _n(j'; w_1)| \\&\qquad - |\Delta _{12} \mu _n(j)| - |\Delta _{12} \mu _{n}(j')| \\&\quad \geqslant \frac{\lambda \gamma }{\langle \ell \rangle ^\tau |j'|^{\tau }} - C \lambda ^{\theta } \big (|j|^{- 1} + |j'|^{- 1}\big ) \Vert w_1 - w_2 \Vert _{s_0 + \Sigma ({\mathtt b})} \\&\quad \geqslant \frac{\lambda \gamma }{\langle \ell \rangle ^\tau |j'|^{\tau }} - 2 C \lambda ^{\theta } \Vert w_1 - w_2 \Vert _{s_0 + \Sigma ({\mathtt b})}\geqslant \frac{\lambda (\gamma - \rho )}{\langle \ell \rangle ^\tau |j'|^{\tau }} , \end{aligned} \end{aligned}$$

provided the following condition holds

5.102 $$\begin{aligned} 2 C \lambda ^{\theta -1} \rho ^{- 1} \langle \ell \rangle ^\tau |j'|^\tau \Vert w_1 - w_2 \Vert _{s_0 + \Sigma ({\mathtt b})} \leqslant 1. \end{aligned}$$

Since $\langle \ell \rangle \leqslant {\mathtt N}_n$ and the momentum conservation $\pi ^\top (\ell ) + j - j' = 0$ implies that $|j - j'| \lesssim |\ell | \lesssim {\mathtt N}_n$, one has that

$$\begin{aligned} |j'| \leqslant \textrm{min} \{ |j|, |j'| \} + |j - j'| \lesssim {\mathtt N}_n^\tau + {\mathtt N}_n \lesssim {\mathtt N}_n^\tau . \end{aligned}$$

Hence the condition (5.102) is implied by

$$\begin{aligned} K \lambda ^{\theta -1} \rho ^{- 1} {\mathtt N}_n^{\tau + \tau ^2} \Vert w_1 - w_2 \Vert _{s_0 + \Sigma ({\mathtt b})} \leqslant 1 \end{aligned}$$

for some large constant $K \gg 1$, and the claimed estimate (5.101) holds.

Finally, Claims 1 and 2 imply that, if $\omega \in \Omega _{n + 1}^\gamma (w_1)$, then, for any $\ell \in {\mathbb {Z}}^\nu {\setminus }\{0\}$ and $j, j' \in {\mathbb {Z}}^2 {\setminus } \{ 0 \}$, with $|\ell | \leqslant {\mathtt N}_n$ and $\pi ^\top (\ell ) + j - j' = 0$, we have that

$$\begin{aligned} | \lambda \, \omega \cdot \ell + \mu _n(j; w_2) - \mu _n(j'; w_2) | \geqslant \frac{\lambda (\gamma - \rho )}{\langle \ell \rangle ^\tau |j'|^\tau } , \end{aligned}$$

which is exactly the fact that $\omega \in \Omega _{n + 1}^{\gamma - \rho }(w_2)$. The proof of $\mathbf{(S4)}_{n+1}$ is then concluded. This concludes also the proof of Proposition 5.9. $\square$

### KAM reducibility: convergence

The eigenvalues of the normal form $\textbf{D}_{n}$ in (5.60) are Cauchy sequences and so they converge to some limit.

#### Lemma 5.11

For any $j \in {\mathbb {Z}}^2 \setminus \{ 0 \}$, the sequence $\{ \widetilde{\mu }_n(j) \}_{n\in {\mathbb {N}}}$, defined in (5.60), converges to some limit

5.103 $$\begin{aligned} \mu _\infty (j) = \textrm{i}\, \beta \,{\mathtt L}(j) + {\mathtt z}_\infty (j), \quad \mu _\infty (j)= \mu _\infty (j;\,\cdot \,):{{\mathtt D}{\mathtt C}(2\gamma ,\tau )\cap \Lambda _{o}}\rightarrow {\mathbb {C}},\nonumber \\ \end{aligned}$$

satisfying the following estimates

5.104 $$\begin{aligned} \begin{aligned}&| \mu _\infty (j) - {{\widetilde{\mu }}}_n(j) |^{\textrm{Lip}(\gamma )} = | {\mathtt z}_\infty (j) - {{\widetilde{{\mathtt z}}}}_n(j) |^{\textrm{Lip}(\gamma )} \lesssim {\mathtt N}_{n - 1}^{- {\mathtt a}} \varepsilon _{M} \gamma ^{-(M-1)} |j|^{- M} , \\&\quad | {\mathtt z}_\infty (j)|^{\textrm{Lip}(\gamma )} \lesssim \lambda ^{\theta } |j|^{- 1} . \end{aligned}\nonumber \\ \end{aligned}$$

In addition, if we assume $w({\varphi },x)=\textrm{odd}({\varphi },x)$, then $\mu _\infty (j;\,\cdot \,):{{\mathtt D}{\mathtt C}(2\gamma ,\tau )\cap \Lambda _{o}}\rightarrow \textrm{i}\,{\mathbb {R}}$ for any $j \in {\mathbb {Z}}^2 {\setminus } \{ 0 \}$.

#### Proof

By Proposition 5.9 and by (5.68), we have that the sequence $\{\widetilde{\mu }_n(j;\omega )\}_{n\in {\mathbb {N}}}\subset {\mathbb {C}}$ is Cauchy on the closed set ${\mathtt D}{\mathtt C}(2\gamma ,\tau )\cap \Lambda _{o}$, therefore it is convergent for any $\omega \in {\mathtt D}{\mathtt C}(2\gamma ,\tau )\cap \Lambda _{o}$. The estimates in (5.104) follow then by a telescoping argument with (5.68). Finally, if we assume $w({\varphi },x)=\textrm{odd}({\varphi },x)$, then $\{\widetilde{\mu }_n(j;\omega )\}_{n\in {\mathbb {N}}}\subset \textrm{i}\,{\mathbb {R}}$ for any $j \in {\mathbb {Z}}^2 {\setminus } \{ 0 \}$, by (5.67), implying that $\mu _\infty (j;\omega ) \in \textrm{i}\,{\mathbb {R}}$ for any $j \in {\mathbb {Z}}^2 {\setminus } \{ 0 \}$. $\square$

We define the set $\Omega _\infty ^\gamma$ of the non-resonance conditions for the final eigenvalues as

5.105 $$\begin{aligned} \begin{aligned}&\Omega _\infty ^\gamma := \Omega _\infty ^\gamma (w):= \Big \{ \omega \in {{\mathtt D}{\mathtt C}(2 \gamma ,\tau ) \cap \Lambda _{o}} \,: \, |\textrm{i}\, \lambda \, \omega \cdot \ell + \mu _\infty (j) - \mu _\infty (j')| \geqslant \frac{2 \lambda \,\gamma }{\langle \ell \rangle ^\tau | j' |^\tau } , \\&\quad \ \ \forall \ell \in {\mathbb {Z}}^\nu \setminus \{ 0 \}, \ \ j,j' \in {\mathbb {Z}}^2 \setminus \{ 0 \}, \ \ \pi ^\top (\ell ) + j-j' =0\Big \}. \end{aligned}\nonumber \\ \end{aligned}$$

#### Lemma 5.12

We have $$\Omega _\infty ^\gamma \subseteq \cap _{n \geqslant 0} \, \Omega _n^\gamma $$.

#### Proof

We prove by induction that $\Omega _\infty ^\gamma \subseteq \Omega _n^\gamma$ for any integer $n \geqslant 0$. The statement is trivial for $n=0$, since $\Omega _0^\gamma := {\mathtt D}{\mathtt C}(2 \gamma ,\tau )\cap \Lambda _{o}$ (see Proposition 5.9).

We now assume by induction that $\Omega _\infty ^\gamma \subseteq \Omega _n^\gamma$ for some $n \geqslant 0$ and we show that $\Omega _\infty ^\gamma \subseteq \Omega _{n + 1}^\gamma$. Let $\omega \in \Omega _\infty ^\gamma$, $\ell \in {\mathbb {Z}}^\nu {\setminus } \{ 0 \}$, $j, j' \in {\mathbb {Z}}^2 {\setminus } \{ 0 \}$, with $\pi ^\top ( \ell ) +j-j'=0$ and $|\ell | \leqslant {\mathtt N}_n$. By (5.104), (5.105), we compute

$$\begin{aligned} \begin{aligned} |\textrm{i}\, \lambda \,\omega \cdot \ell + \mu _n(j) - \mu _n(j')|&\geqslant |\textrm{i}\, \lambda \,\omega \cdot \ell + \mu _\infty (j) - \mu _\infty (j')| - |\mu _\infty (j) - \mu _n(j)| \\&\quad - |\mu _\infty (j') - \mu _n(j')| \\&\geqslant \frac{2\lambda \,\gamma }{\langle \ell \rangle ^\tau | j' |^\tau } - C {\mathtt N}_{n - 1}^{- {\mathtt a}} \varepsilon _{M} \gamma ^{-(M-1)}\big ( |j|^{- M} + |j'|^{- M} \big ) \\&\geqslant \frac{\lambda \,\gamma }{\langle \ell \rangle ^\tau | j' |^\tau } , \end{aligned} \end{aligned}$$

for some positive constant $C>0$, provided

5.106 $$\begin{aligned} C {\mathtt N}_{n - 1}^{- {\mathtt a}}\varepsilon ^{M}\gamma ^{- M}\langle \ell \rangle ^\tau \big ( | j' |^\tau |j|^{- M} + |j'|^{\tau - M} \big ) \leqslant 1. \end{aligned}$$

Recalling that $M > \tau$ by (5.56), we have that $|j'|^{\tau - M} \leqslant 1$. Moreover, using that $|\ell | \leqslant {\mathtt N}_n$ and the momentum condition $\pi ^\top (\ell ) + j-j'=0$, we have that

$$\begin{aligned} \begin{aligned}&|j'| \leqslant |j| + |j - j'| \leqslant |j| + {\mathtt N}_n \lesssim {\mathtt N}_n |j|, \end{aligned} \end{aligned}$$

and therefore, using again that $M > \tau$, we have

$$\begin{aligned} | j' |^\tau |j|^{- M} \lesssim {\mathtt N}_n^\tau |j|^{\tau - M} \lesssim {\mathtt N}_n^\tau . \end{aligned}$$

We then deduce that

$$\begin{aligned} C {\mathtt N}_{n - 1}^{- {\mathtt a}}\varepsilon ^{M}\gamma ^{- M}\langle \ell \rangle ^\tau \Big ( | j' |^\tau |j|^{- M} + |j'|^{\tau - M} \Big ) \leqslant C_0 {\mathtt N}_n^{2 \tau } {\mathtt N}_{n - 1}^{- {\mathtt a}} \varepsilon ^{M} \gamma ^{- M} \end{aligned}$$

for some large constant $C_0 \gg 0$. Hence, the condition (5.106) is verified since

$$\begin{aligned} C_0 {\mathtt N}_n^{2 \tau } {\mathtt N}_{n - 1}^{- {\mathtt a}} \varepsilon ^{M} \gamma ^{- M} \leqslant 1, \end{aligned}$$

recalling (5.56), $\varepsilon \gamma ^{-1} = \lambda ^{\theta -1}\gamma ^{-1}\ll 1$ in (4.7) and the smallness condition (5.59) We conclude that $\omega \in \Omega _{n + 1}^\gamma$ and the claim is proved. $\square$

Now we define the sequence of invertible maps

5.107 $$\begin{aligned} {{\widetilde{\Phi }}}_n:= \Phi _0 \circ \Phi _1 \circ \ldots \circ \Phi _n , \quad n \in {\mathbb {N}}. \end{aligned}$$

#### Proposition 5.13

Let $S > s_0 + \Sigma ({\mathtt b})$. There exists $\delta := \delta (S, \tau ,\nu ) > 0$ such that, if (4.2) (5.59) are verified, then the following holds. For any $\omega \in \Omega _\infty ^\gamma$, the sequence $({{\widetilde{\Phi }}}_n)_{n\in {\mathbb {N}}}$ converges in norm $| \cdot |_{0, s}^{\textrm{Lip}(\gamma )}$ to an invertible map $\Phi _\infty$, satisfying, for any $s_0\leqslant s\leqslant S-\Sigma ({\mathtt b})$,

5.108 $$\begin{aligned} \begin{aligned}&|\Phi _\infty ^{\pm 1} - {{\widetilde{\Phi }}}_n^{\pm 1}|_{0, s}^{\textrm{Lip}(\gamma )} \lesssim _{s} {\mathtt N}_{n + 1}^{2 \tau + 1} {\mathtt N}_n^{- {\mathtt a}} \varepsilon ^{M} \gamma ^{- M} \Vert w \Vert _{s + \Sigma ({\mathtt b})}^{\textrm{Lip}(\gamma )} , \\&\quad |\Phi _\infty ^{\pm 1} - \textrm{Id}|_{0, s}^{\textrm{Lip}(\gamma )} \lesssim _{s} {\mathtt N}_{0}^{2\tau +1}\varepsilon ^{M} \gamma ^{- M} \Vert w \Vert _{s + \Sigma ({\mathtt b})}^{\textrm{Lip}(\gamma )} . \end{aligned} \end{aligned}$$

The operators $\Phi _\infty ^{\pm 1}: H^s_0 \rightarrow H^s_0$ are real and momentum preserving. Moreover, for any $\omega \in \Omega _\infty ^\gamma$, one has the conjugation

5.109 $$\begin{aligned} {\textbf{L}}_{\infty }:= \Phi _\infty ^{- 1} {\textbf{L}}_{0} \Phi _\infty =\lambda \, \omega \cdot \partial _\varphi + {\textbf{D}}_\infty , \quad {\textbf{D}}_\infty := \textrm{diag}_{j \in {\mathbb {Z}}^2 \setminus \{ 0 \}} \mu _\infty (j) , \end{aligned}$$

where the operator $\textbf{L}_{0}$ is given in (5.55)

and the final eigenvalues $\mu _\infty (j)$ are given in Lemma 5.11. In addition, if we assume that $w({\varphi },x)=\textrm{odd}({\varphi },x)$, the operators $\Phi _{\infty }^{\pm 1}$ are reversibility preserving and $\textbf{D}_\infty$ is a reversible operator.

#### Proof

The existence of the invertible map $\Phi _\infty ^{\pm 1}$ and the estimates (5.108) follow by (5.66), (5.107), arguing as in Corollary 4.1 in [6]. By (5.107), Lemma 5.12 and Proposition 5.9, one has ${{\widetilde{\Phi }}}_n^{- 1} {\textbf{L}}_0 {{\widetilde{\Phi }}}_n =\lambda \, \omega \cdot \partial _\varphi + {\textbf{D}}_n + {\textbf{E}}_n$ for all $n \geqslant 0$. The claimed statement then follows by passing to the limit as $n \rightarrow \infty$, by using (5.64), (5.108) and Lemma 5.11. $\square$

## Inversion of the Linearized Operator ${{\mathcal {L}}}$

In this section we show that the linearized operator ${{\mathcal {L}}}$ in (4.3) is invertible and we prove tame estimates for its inverse. By Propositions 5.1, 5.5, 5.6, 5.13, using also Lemma 2.6-(*i*), for any $\omega \in \Omega _\infty ^\gamma$, we define

6.1 $$\begin{aligned} {{\mathcal {W}}}_\infty := {\mathcal {B}}_{\perp } \circ \mathbf{\Phi }_M \circ \Phi _\infty \end{aligned}$$

and we have that

$$\begin{aligned} {{\mathcal {W}}}_\infty ^{- 1} {{\mathcal {L}}} {{\mathcal {W}}}_\infty = {\textbf{L}}_{\infty } , \quad \forall \,\omega \in \Omega _\infty ^\gamma , \end{aligned}$$

with the maps ${{\mathcal {W}}}_\infty \,,\, {{\mathcal {W}}}_\infty ^{- 1}: H^s_0({\mathbb {T}}^{\nu + 2}) \rightarrow H^s_0({\mathbb {T}}^{\nu + 2})$ are real and momentum preserving and, if (4.2) holds with $\sigma _0 = \Sigma (\mathtt b)$, they satisfy for any $s_0 \leqslant s \leqslant S-\Sigma ({\mathtt b})$, the bounds

6.2 $$\begin{aligned} \Vert {{\mathcal {W}}}_\infty ^{\pm 1} h \Vert _s^{\textrm{Lip}(\gamma )} \lesssim _s \Vert h \Vert _s^{\textrm{Lip}(\gamma )} + \Vert w \Vert _{s + \Sigma ({\mathtt b})}^{\textrm{Lip}(\gamma )} \Vert h \Vert _{s_0}^{\textrm{Lip}(\gamma )}. \end{aligned}$$

In addition, if $w = \textrm{odd}({\varphi }, x)$, then ${{\mathcal {W}}}_\infty , {{\mathcal {W}}}_\infty ^{- 1}$ are reversibility preserving.

Now we define the set $\Lambda _\infty ^\gamma$ as

6.3 $$\begin{aligned} \begin{aligned}&\Lambda _\infty ^\gamma := \Lambda _\infty ^\gamma (w):= \Big \{ \omega \in {{\mathtt D}{\mathtt C}(2 \gamma , \tau ) \cap \Lambda _{o}} \,: \, |\textrm{i}\,\lambda \,\omega \cdot \ell + \mu _\infty (j) | \geqslant \frac{2 \lambda \, \gamma }{\langle \ell \rangle ^\tau } , \\&\quad \ \forall \, \ell \in {\mathbb {Z}}^\nu \setminus \{0\} ,\ \ j \in {\mathbb {Z}}^2 \setminus \{ 0 \}, \ \ \pi ^\top (\ell ) + j = 0 \Big \}. \end{aligned} \end{aligned}$$

First, we discuss the invertibility of the diagonal operator $\textbf{L}_\infty$ given in (5.109).

### Lemma 6.1

For any $\omega \in \Lambda _\infty ^\gamma$, the linear operator $\textbf{L}_\infty (\omega )$ is invertible and its inverse $\textbf{L}_\infty ^{- 1}$ satisfies the bound, for any $s\geqslant 0$,

6.4 $$\begin{aligned} \Vert \textbf{L}_\infty ^{- 1} h \Vert _s^{\textrm{Lip}(\gamma )} \lesssim \lambda ^{- 1} \gamma ^{- 1} \Vert h \Vert _{s + 2 \tau + 1}^{\textrm{Lip}(\gamma )} . \end{aligned}$$

In addition, if $w = \textrm{odd}({\varphi }, x)$, then $\textbf{L}_\infty ^{- 1}$ is reversible.

### Proof

If $\omega \in \Lambda _\infty ^\gamma$, the inverse $\textbf{L}_\infty (\omega )^{- 1}$ is defined by

6.5 $$\begin{aligned} \textbf{L}_\infty (\omega )^{- 1} h(\varphi , x) = \sum _{ \ell \in {\mathbb {Z}}^\nu \setminus \{0\},\, j \in {\mathbb {Z}}^2 \setminus \{ 0 \} \atop \pi ^\top (\ell ) + j = 0 } \frac{1}{\eta _{\ell j}(\omega )} \widehat{h}(\ell , j) e^{\textrm{i}\ell \cdot \varphi } e^{\textrm{i}j \cdot x} , \end{aligned}$$

where $\eta _{\ell j }(\omega ) = \textrm{i}\,\lambda \,\omega \cdot \ell + \mu _\infty (j;\omega )$ and we used the momentum condition $\pi ^\top (\ell ) + j = 0$ to get the restriction $\ell \ne 0$, since we already have $j \ne 0$. This clearly implies the bound, by (6.3), for any $s\geqslant 0$,

6.6 $$\begin{aligned} \Vert \textbf{L}_\infty (\omega )^{- 1} h \Vert _s \lesssim \lambda ^{- 1} \gamma ^{- 1} \Vert h \Vert _{s + \tau }. \end{aligned}$$

Moreover, given $\omega _1, \omega _2 \in \Lambda _\infty ^\gamma$, by (6.3) Lemma 5.11 and using $\lambda ^{\theta -1}\gamma ^{-1}\leqslant 1$ in (4.7), we have that

$$\begin{aligned} \begin{aligned} \Big | \frac{1}{\eta _{\ell j}(\omega _1)} - \frac{1}{\eta _{\ell j}(\omega _2)} \Big |&\lesssim \frac{\lambda \,|\ell | |\omega _1 - \omega _2| + |\mu _\infty (j; \omega _1) - \mu _\infty (j; \omega _2)|}{|\eta _{\ell j}(\omega _1)| |\eta _{\ell j}(\omega _2)|} \\&\lesssim \langle \ell \rangle ^{2 \tau }\lambda ^{- 2} \gamma ^{- 2} \big ( \lambda \,|\ell | + \lambda ^{\theta } \gamma ^{- 1} \big ) |\omega _1 - \omega _2| \\&\lesssim \lambda ^{- 1} \gamma ^{- 2} \langle \ell \rangle ^{2 \tau + 1} |\omega _1 - \omega _2| , \end{aligned} \end{aligned}$$

which implies that, for any $s\geqslant 0$,

6.7 $$\begin{aligned} \big \Vert \big ( \textbf{L}_\infty (\omega _1)^{- 1} - \textbf{L}_\infty (\omega _2)^{- 1}\big ) h \big \Vert _s \lesssim \lambda ^{- 1} \gamma ^{- 2} \Vert h \Vert _{s + 2 \tau + 1} . \end{aligned}$$

Now, using that, for any $h(\omega ) \in H^{s + 2 \tau + 1}_0$ and any $\omega _1, \omega _2 \in \Lambda _\infty ^\gamma$,

$$\begin{aligned} \begin{aligned}&\Vert \textbf{L}_\infty (\omega _1)^{- 1} h(\omega _1) - \textbf{L}_\infty (\omega _2)^{- 1} h(\omega _2) \Vert _s \\&\quad \leqslant \Vert \textbf{L}_\infty (\omega _1)^{- 1}\big ( h(\omega _1) - h(\omega _2) \big ) \Vert _s + \Vert \big ( \textbf{L}_\infty (\omega _1)^{- 1} - \textbf{L}_\infty (\omega _2)^{- 1} \big ) h(\omega _2) \Vert _s , \end{aligned} \end{aligned}$$

then, together with the bounds (6.6), (6.7), we obtain the claimed estimate (6.4). Finally, if we assume that $w = \textrm{odd}({\varphi },x)$, then $\textbf{L}_{\infty }, \textbf{L}_\infty ^{- 1}$ are reversible operators, by (6.5), Lemma 5.11 and Lemma 2.9. $\square$

We are now in position to state the following proposition on the invertibility of the linearized operator ${\mathcal {L}}$ in (4.3).

### Proposition 6.2

Let ${\overline{\sigma }}:= \Sigma ({\mathtt b}) + 2 \tau + 1$, with $\Sigma ({\mathtt b})$ given in (5.56). Let $S > s_0 + {\overline{\sigma }}$. There exists $\delta := \delta (S, \tau , \nu ) > 0$ such that, if (4.2) (5.59) are verified (with $\sigma = {\overline{\sigma }}$), then the following holds. For any $\omega \in \Omega _\infty ^\gamma \cap \Lambda _\infty ^\gamma$ the linear operator ${\mathcal {L}}={{\mathcal {L}}}(\omega )$, defined (4.3), is invertible on the subspace of the quasi-periodic traveling waves $\tau _\varsigma h({\varphi })=h({\varphi }-\pi (\varsigma ))$, $\varsigma \in {\mathbb {R}}$, and its inverse ${\mathcal {L}}^{-1}={{\mathcal {L}}}(\omega )^{- 1}$ satisfies the tame bound, for any $s_0 \leqslant s \leqslant S-{\overline{\sigma }}$ and any $h\in H^{s + {\overline{\sigma }}}_0({\mathbb {T}}^{\nu +2})$

6.8 $$\begin{aligned} \Vert {{\mathcal {L}}}^{- 1} h \Vert _s^{\textrm{Lip}(\gamma )} \lesssim _s \lambda ^{- 1} \gamma ^{- 1}\Big ( \Vert h \Vert _{s + {\overline{\sigma }}}^{\textrm{Lip}(\gamma )} + \Vert w \Vert _{s + {\overline{\sigma }}}^{\textrm{Lip}(\gamma )} \Vert h \Vert _{s_0 + \overline{\sigma }}^{\textrm{Lip}(\gamma )} \Big ). \end{aligned}$$

Moreover, ${\mathcal {L}}^{-1}h$ is a quasi-periodic traveling wave. In addition, if $w = \textrm{odd}({\varphi }, x)$, then ${{\mathcal {L}}}^{- 1}$ is reversible.

### Proof

For any $\omega \in \Omega _\infty ^\gamma \cap \Lambda _\infty ^\gamma$, one has that ${{\mathcal {L}}}^{- 1} = {\mathcal W}_\infty ^{- 1} \textbf{L}_\infty ^{- 1} {{\mathcal {W}}}_\infty$. Hence, the estimate (6.8) follows by (6.2) and Lemma 6.1, using also the ansatz (4.2) with $\sigma = {\overline{\sigma }}$. Since $\textbf{L}_{\infty }^{\pm 1}$ are momentum preserving by (5.109), (6.5), and the maps $\mathcal {W}_{\infty }^{\pm 1}$ are momentum preserving by (6.1) and Propositions 5.1, 5.6, 5.13, then, by Lemmata 2.12, 2.13, we conclude that ${\mathcal {L}}^{-1}h$ is a quasi-periodic traveling wave, whenever is *h*. Finally, if we assume that $w = \textrm{odd}({\varphi },x)$, then ${\mathcal {L}}^{-1}$ is reversible by Lemma 6.1 and by the fact that $\mathcal {W}_{\infty }^{\pm 1}$ are reversibility preserving. $\square$

## The Nash Moser scheme

In this section we construct the solution of the equation ${\mathcal F}(v) = 0$ in (3.1) by means of a Nash Moser nonlinear iteration. We denote by $\Pi _n$ the orthogonal projector $\Pi _{{\mathtt N}_n}$ (on quasi-periodic traveling waves, see (2.6)) on the finite dimensional space

$$\begin{aligned} {{\mathcal {H}}}_n:= \big \{ w \in L_0^2({\mathbb {T}}^{\nu + 2}, {\mathbb {R}}) \, \ \text { such that } (2.1.1) \text { holds } \, \,: \, \ w = { \Pi }_n w \big \} , \end{aligned}$$

and $\Pi _n^\bot := \textrm{Id} - \Pi _n$, where $L_0^2({\mathbb {T}}^{\nu +2})=H_0^0({\mathbb {T}}^{\nu +2})$, see (1.10). The projectors $\Pi _n$, $\Pi _n^\bot$ satisfy the usual smoothing properties in Lemma 2.4, namely

7.1 $$\begin{aligned} \Vert \Pi _{n} v \Vert _{s+b}^{\textrm{Lip}(\gamma )} \leqslant {\mathtt N}_{n}^{b} \Vert v \Vert _{s}^{\textrm{Lip}(\gamma )} , \quad \ \Vert \Pi _{n}^\bot v \Vert _{s}^{\textrm{Lip}(\gamma )} \leqslant {\mathtt N}_n^{- b} \Vert v \Vert _{s + b}^{\textrm{Lip}(\gamma )} , \quad \ s,b \geqslant 0 . \end{aligned}$$

Given $\tau , {\mathtt N}_{0}>0$, we define the constants

7.2 $$\begin{aligned} \begin{aligned}&{\mathtt N}_{n}:= {\mathtt N}_{0}^{\chi ^n}, \quad n \geqslant 0, \quad {\mathtt N}_{- 1}:= 1, \quad \chi = 3/2, \quad \kappa := 12( {\overline{\sigma }} + 1) + 1, \\&\quad \mathtt a_1:= \textrm{max}\{ 6 {\overline{\sigma }} + 13,\, \chi ^2(\tau + \tau ^2 + 1) + \chi (2 {\overline{\sigma }} + 1) + 1 \} , \\&\quad {\overline{\tau }}:= 2 {\overline{\sigma }} + 4 + \mathtt a_1 + \chi (\tau + \tau ^2 + 1)+\tau _{1}, \quad {\mathtt b}_1:= 2 {\overline{\sigma }} + 4 +\chi ^{-1} ( \mathtt a_1 + \kappa ). \end{aligned} \end{aligned}$$

where ${\overline{\sigma }} > 1$ is given in Proposition 6.2 and $\tau _{1}>1$ is fixed in (5.56).

### Remark 7.1

Let us describe of the parameters in (7.2) and their role in the following Proposition 7.3. The parameter ${\mathtt a}_{1}>0$ appears in the negative exponent of the estimate in (7.8) and measures how the low regularity norm of the nonlinear functional is fastly decreasing to 0 at each approximate solution of the iteration. The parameter ${\mathtt b}_{1}>0$ appears in the regularity index $s_0+{\mathtt b}_{1}$ of the second estimate in (7.9), which is the high regularity norms of the approximate solution and the functional evaluated at it that are allowed to diverge, with exponent rate $\kappa >0$. The parameter ${\overline{\tau }}>0$ appears as an exponent in the smallness condition (7.4). The parameter ${\overline{\sigma }}>0$ accounts for the loss of derivatives of the KAM iteration. The role of the parameters in (7.2) is comparable with the ones in (5.56) for the KAM reducibility of the linearized operators (see also Remark 5.8). The differences are in their definitions, due to the nonlinear iteration and the loss of derivative ${\overline{\sigma }}>\Sigma ({\mathtt b})>0$ (see Proposition 6.2), and the exponent $\kappa >0$ in the divergence of the high regularity norms.

Note that Proposition 3.3 provides an approximate solution $v_{\textrm{app},N}$ which is a quasi periodic traveling wave such that, for any $s\geqslant 0$, $\Vert v_{\textrm{app},N}\Vert _{s}^{\textrm{Lip}(\gamma )}$ is of size 1 and $\Vert {\mathcal F}(v_{\textrm{app},N}) \Vert _s^{\textrm{Lip}(\gamma )}$ is uniformly bounded with respect to $\lambda \gg 1$. We state these properties as follows.

### Lemma 7.2

(**Initialization of the Nash-Moser iteration**). Let $N\in {\mathbb {N}}$ and $\gamma \in (0,1)$ as in (3.15). For any $s \geqslant 0$, there exists a constant $C(s)>0$ such that

7.3 $$\begin{aligned} \begin{aligned}&\Vert {{\mathcal {F}}}(v_{\textrm{app},N}) \Vert _s^{\textrm{Lip}(\gamma )} \leqslant C(s),\quad \Vert v_{\textrm{app},N} \Vert _s^{\textrm{Lip}(\gamma )} \leqslant C(s), \\&\quad \inf _{\omega \in \Omega _\gamma } \Vert v_{\textrm{app, N}}(\cdot ; \omega )\Vert _s \geqslant K(s) \lambda ^{- \mathtt c} \end{aligned} \end{aligned}$$

uniformly in $\lambda \geqslant {\overline{\lambda }} \gg 1$ sufficiently large, for some constants $C(s), K(s) > 0$. In addition, if the forcing term *f* in (3.1) is $\textrm{even}({\varphi }, x)$, then $v_{\textrm{app, N}} = \textrm{odd}({\varphi }, x)$.

The lower bound in (7.3), that has been proved in Proposition 3.3, is actually not needed in the nonlinear iteration. It will be used to prove Theorem 1.3 in the next section.

We now prove the following proposition.

### Proposition 7.3

(**Nash-Moser**). Let $\tau > 0$, and let $\Omega _{\gamma }$ be defined in (3.6) With the notation in (7.2), there exist $\delta \in (0, 1)$, $C_* > 0$ such that if

7.4 $$\begin{aligned} \begin{aligned}&{\mathtt N}_{0}^{{\overline{\tau }}} \lambda ^{- 1} \leqslant \delta , \quad {\mathtt N}_{0}:= \gamma ^{- 1}, \quad \gamma := \lambda ^{- \mathtt c}, \quad \text {for some} \ \ \ 0< \mathtt c < \textrm{min} \{ {\overline{\tau }}\,^{-1}, \tfrac{1}{3}(2-\alpha ) \}, \end{aligned}\nonumber \\ \end{aligned}$$

then the following properties hold for all $n \geqslant 0$:

$({{\mathcal {P}}}1)_{n}$ There exist a constant $C_0 > 0$ large enough and $w_n - w_0: {{\mathcal {G}}}_n \rightarrow {{\mathcal {H}}}_{n - 1}$, $n \geqslant 1$, $w_0:= v_{\textrm{app},N}$, ${{\mathcal {H}}}_{- 1}:= \{ 0 \}$, satisfying

7.5 $$\begin{aligned} \Vert w_{n} \Vert _{s_0 + {\overline{\sigma }}}^{\textrm{Lip}(\gamma )} \leqslant C_0 . \end{aligned}$$

If $$n \geqslant 1$$, the difference $$ h_n:= w_n - w_{n - 1}$$ satisfies $$\Vert h_1 \Vert _{s_0 + {\overline{\sigma }}}^{\textrm{Lip}(\gamma )} \lesssim \lambda ^{- 1} \gamma ^{-1}$$ and

7.6 $$\begin{aligned} \Vert h_n \Vert _{s_0 + {\overline{\sigma }}}^{\textrm{Lip}(\gamma )} \leqslant C_* {\mathtt N}_{n-1}^{2 {\overline{\sigma }} + 1} {\mathtt N}_{n-2}^{-\mathtt a_1} \lambda ^{- 1} . \end{aligned}$$

The sets $\{ {{\mathcal {G}}}_n\}_{n \geqslant 0}$ are defined as follows. For $n\in {\mathbb {N}}_{0}$ we define ${{\mathcal {G}}}_0:= \Omega _\gamma$ and

7.7 $$\begin{aligned} {{\mathcal {G}}}_{n + 1}:= {{\mathcal {G}}}_n \cap \big ( \Omega _\infty ^{\gamma _n}(w_n) \cap \Lambda _\infty ^{\gamma _n}(w_n) \big ), \end{aligned}$$

where $\gamma _{n}:=\gamma (1 + 2^{-n})$ and the sets $\Omega _\infty ^{\gamma _n}(w_n)$, $\Lambda _\infty ^{\gamma _n}(w_n)$ are defined in (5.105), (6.3), with $\Lambda _{o}:=\mathcal {G}_{n}$. Moreover, the function $w_n({\varphi },x;\omega )$ is a quasi-periodic traveling wave. In addition, if the forcing term *f* in (3.1) is $\textrm{even}({\varphi }, x)$, then $w_n$ is $\textrm{odd}({\varphi }, x)$;

$({{\mathcal {P}}}2)_{n}$ On the set ${{\mathcal {G}}}_n$, we have the estimate

7.8 $$\begin{aligned} \Vert {{\mathcal {F}}}(w_n) \Vert _{s_{0}}^{\textrm{Lip}(\gamma )} \leqslant C_* {\mathtt N}_{n - 1 }^{- \mathtt a_1}\,; \end{aligned}$$

$({{\mathcal {P}}}3)_{n}$ On the set ${{\mathcal {G}}}_n$, we have the estimate

7.9 $$\begin{aligned} \Vert w_n \Vert _{s_0 + \mathtt b_1}^{\textrm{Lip}(\gamma )}+ \Vert {\mathcal {F}}(w_n) \Vert _{s_0 + \mathtt b_1}^{\textrm{Lip}(\gamma )} \leqslant C_* {\mathtt N}_{n-1}^{\kappa } . \end{aligned}$$

### Proof

To simplify notations, in this proof we write $\Vert \cdot \Vert _s$ instead of $\Vert \cdot \Vert _s^{\textrm{Lip}(\gamma )}$.

Proof of $({{\mathcal {P}}}1, 2, 3)_0$. By (7.3) with $s=s_0,s_0+{\mathtt b}_{1}$, we have $\Vert w_0\Vert _{s} = \Vert v_{\textrm{app},N} \Vert _{s} \leqslant C(s)$ and $\Vert {{\mathcal {F}}} (w_0 ) \Vert _s = \Vert {{\mathcal {F}}} (v_{\textrm{app},N} ) \Vert _s \leqslant C(s)$. Then (7.5), (7.8) and (7.9) hold taking $\tfrac{1}{2} C_0, \tfrac{1}{2}C_*(s) \geqslant C(s)$ sufficiently large. In particular, we have

7.10 $$\begin{aligned} \Vert w_0 \Vert _{s_0+{\overline{\sigma }}} \leqslant \tfrac{1}{2} C_0 \leqslant C_0. \end{aligned}$$

Assume that $({{\mathcal {P}}}1,2,3)_n$ hold for some $n \geqslant 0$, and prove $({{\mathcal {P}}}1,2,3)_{n+1}$. Let $\Lambda _{o}:=\mathcal {G}_{n}$. By $({{\mathcal {P}}}1)_n$, one has $\Vert w_n\Vert _{s_0 + {\overline{\sigma }}} \leqslant C_0$, for some $C_0 \gg 0$ large enough, independent of $n\in {\mathbb {N}}_0$. The assumption (7.4) implies the smallness condition (5.59) of Proposition 5.9. Indeed, by the definition of ${\overline{\tau }}$ in (7.2), we have that

$$\begin{aligned} {\overline{\tau }} > \tau _{1} +1 , \quad \text {that is} \quad \tau _{1}<{\overline{\tau }}-1 . \end{aligned}$$

Moreover, by (7.4), (5.58), (4.5), we have that $\varepsilon \gamma ^{-1}=(\lambda ^{-1})^{2(1-{\mathtt c})-\alpha }\ll 1$, which implies, using that $M>\frac{1-{\mathtt c}}{2(1-{\mathtt c})-\alpha }$ by (5.56),

$$\begin{aligned} \varepsilon ^M \gamma ^{-M} = (\lambda ^{-1})^{(2(1-{\mathtt c})-\alpha )M} < (\lambda ^{-1})^{1-{\mathtt c}} . \end{aligned}$$

We conclude that, if ${\mathtt N}_{0}>1$ satisfies (7.4) for $\lambda \gg 1$ large enough, then

$$\begin{aligned} {\mathtt N}_{0}^{\tau _{1}} \varepsilon ^M \gamma ^{-M}< {\mathtt N}_{0}^{{\overline{\tau }}} {\mathtt N}_{0}^{-1} \varepsilon ^M \gamma ^{-M} < {\mathtt N}_{0}^{{\overline{\tau }}} {\mathtt N}_{0}^{-1}(\lambda ^{-1})^{1-{\mathtt c}} = {\mathtt N}_{0}^{{\overline{\tau }}} \lambda ^{-{\mathtt c}} \lambda ^{{\mathtt c}-1} = {\mathtt N}_{0}^{{\overline{\tau }}} \lambda ^{-1} \leqslant \delta , \end{aligned}$$

that is, (5.59) is satisfied as well, as claimed. Then Proposition 6.2 applies to the linearized operator

$$\begin{aligned} {{\mathcal {L}}}_n \equiv {{\mathcal {L}}}(w_n): = \textrm{d}_{v} {\mathcal F}(w_n) . \end{aligned}$$

This implies that, for any $\omega \in {{\mathcal {G}}}_{n + 1}$, the operator ${{\mathcal {L}}}_n(\omega )$ admits a right inverse ${\mathcal L}_n(\omega )^{- 1}$ satisfying the tame estimates, for any $s_0 \leqslant s \leqslant s_0 + {\mathtt b}_{1}$,

7.11 $$\begin{aligned} \Vert {{\mathcal {L}}}_n^{-1} h \Vert _s \lesssim _s \lambda ^{- 1}\gamma ^{-1} \big ( \Vert h \Vert _{s + {\overline{\sigma }}} + \Vert w_n \Vert _{s + \overline{\sigma }} \, \Vert h \Vert _{s_0 + {\overline{\sigma }}} \big ), \end{aligned}$$

using the bound $\gamma _n = \gamma (1 + 2^{- n}) \in [\gamma , 2\gamma ]$. By (7.11) for $s= s_0$ and (7.5), one has

7.12 $$\begin{aligned} \Vert {{\mathcal {L}}}_n^{- 1} h \Vert _{s_0} \lesssim \lambda ^{- 1}\gamma ^{- 1} \Vert h \Vert _{s_0 + {\overline{\sigma }}}. \end{aligned}$$

We define the successive approximation

7.13 $$\begin{aligned} w_{n + 1}:= w_n + h_{n + 1} , \quad {h}_{n + 1}:= - \Pi _n {{\mathcal {L}}}_n^{-1} \Pi _n {{\mathcal {F}}}(w_n) \in {{\mathcal {H}}}_{n} , \end{aligned}$$

defined for any $\omega \in {{\mathcal {G}}}_{n + 1}$, and the remainder

$$\begin{aligned} Q_n:= {{\mathcal {F}}}(w_{n+1}) - {{\mathcal {F}}}(w_n) - {{\mathcal {L}}}_n h_{n+1}. \end{aligned}$$

We now estimate $h_{n + 1}$. Note that for any $s, \mu \geqslant 0$,

7.14 $$\begin{aligned} \begin{aligned}&\Vert w_n \Vert _{s+\mu } \leqslant \Vert w_0 \Vert _{s + \mu } + \Vert w_n - w_0 \Vert _{s + \mu } {\mathop {\lesssim _{s, \mu }}\limits ^{w_0 = v_{\textrm{app, N}},\, (7.2)}} 1 + {\mathtt N}_{n - 1}^\mu \Vert w_n - w_0 \Vert _s \\&\quad \lesssim _{s, \mu } {\mathtt N}_{n - 1}^\mu (1 + \Vert w_n \Vert _s)\, \quad \text {and hence by } (7), \\&\quad \Vert {{\mathcal {L}}}_n^{- 1} h \Vert _{s_0 + 1} \lesssim {\mathtt N}_{n - 1}\lambda ^{- 1}\gamma ^{- 1} \Vert h \Vert _{s_0 + {\overline{\sigma }} + 1} \end{aligned} \end{aligned}$$

By the estimates (7.11), (7.12), (7.5), (7.1), (7.2), (7.4), (7.8), (7.14), by Lemma (3.1)-(*i*), we get that

7.15 $$\begin{aligned} \begin{aligned} \Vert h_{n + 1} \Vert _{s_0}&\lesssim \lambda ^{- 1}\gamma ^{- 1} \Vert \Pi _n {{\mathcal {F}}}(w_n) \Vert _{s_0 + {\overline{\sigma }}} \lesssim {\mathtt N}_n^{{\overline{\sigma }}} \lambda ^{- 1} \gamma ^{- 1} \Vert {{\mathcal {F}}}(w_n) \Vert _{s_0} , \\ \Vert h_{n + 1} \Vert _{s_0 + {\overline{\sigma }}}&\lesssim {\mathtt N}_n^{{\overline{\sigma }}} \Vert {{\mathcal {L}}}_n^{-1} \Pi _n {{\mathcal {F}}}(w_n) \Vert _{s_0} \lesssim {\mathtt N}_n^{2 {\overline{\sigma }}} \lambda ^{- 1} \gamma ^{- 1} \Vert {\mathcal F}(w_n) \Vert _{s_0}. \end{aligned} \end{aligned}$$

and

7.16 $$\begin{aligned} \begin{aligned} \Vert h_{n + 1} \Vert _{s_0 + \mathtt b_1}&\lesssim \Vert {\mathcal {L}}_{n}^{-1} \Pi _{n} {\mathcal {F}}(w_n) \Vert _{s_0+{\mathtt b}_{1}} \\&\lesssim \lambda ^{- 1} \gamma ^{- 1} \Big ( \Vert \Pi _n {{\mathcal {F}}}(w_n) \Vert _{s_0 + \mathtt b_1 + {\overline{\sigma }}} + \Vert w_n \Vert _{s_0 + \mathtt b_1 + {\overline{\sigma }}} \Vert \Pi _n {{\mathcal {F}}}(w_n) \Vert _{s_0 + {\overline{\sigma }}} \Big ) \\&\lesssim \lambda ^{- 1} \gamma ^{- 1} {\mathtt N}_n^{2 {\overline{\sigma }} } \Big ( \Vert {{\mathcal {F}}}(w_n) \Vert _{s_0 + \mathtt b_1} + \Vert w_n \Vert _{s_0 + \mathtt b_1 } \Vert {{\mathcal {F}}}(w_n) \Vert _{s_0} \Big ) \\&\lesssim \lambda ^{- 1} {\mathtt N}_n^{2 {\overline{\sigma }} +1} \Big ( \Vert {{\mathcal {F}}}(w_n) \Vert _{s_0 + \mathtt b_1} +C_* {\mathtt N}_{n-1}^{-{\mathtt a}_{1}} \Vert w_n \Vert _{s_0 + \mathtt b_1 } \Big ) \\&\lesssim \lambda ^{- 1} {\mathtt N}_n^{2 {\overline{\sigma }} +1} \Big ( \Vert {{\mathcal {F}}}(w_n) \Vert _{s_0 + \mathtt b_1} + \Vert w_n \Vert _{s_0 + \mathtt b_1 } \Big ). \end{aligned} \end{aligned}$$

Moreover, (7.13) and estimate (7.16), using that $\lambda ^{-1}\ll 1$, imply that

7.17 $$\begin{aligned} \begin{aligned} \Vert w_{n + 1} \Vert _{s_0 + \mathtt b_1}&\leqslant \Vert w_n \Vert _{s_0 + \mathtt b_1} + \Vert h_{n + 1} \Vert _{s_0 + \mathtt b_1} \\&\lesssim {\mathtt N}_{n}^{2 {\overline{\sigma }} + 1} \Big ( \Vert {{\mathcal {F}}}(w_n) \Vert _{s_0 + \mathtt b_1} + \Vert w_n \Vert _{s_0 + \mathtt b_1 } \Big ). \end{aligned} \end{aligned}$$

Next, we estimate $\Vert {{\mathcal {F}}}(w_{n + 1}) \Vert _{s_0}$. By the definition of $h_{n+1}$ in (7.13), recalling that $\Pi _{n} + \Pi _{n}^\perp =\textrm{Id}$ and $\Pi _{n}^\perp \Pi _{n} = 0$, we obtain that, for any $\omega \in {{\mathcal {G}}}_{n + 1}$,

7.18 $$\begin{aligned} \begin{aligned} {{\mathcal {F}}}(w_{n + 1})&= {{\mathcal {F}}}(w_n) + {{\mathcal {L}}}_n h_{n + 1} + Q_n \\&= {\mathcal {F}}(w_n) - {\mathcal {L}}_{n} (\textrm{Id} - \Pi _{n}^\perp ) {\mathcal {L}}_{n}^{-1} \Pi _{n} {\mathcal {F}}(w_n) + Q_n \\&= \Pi _n^\bot {{\mathcal {F}}}(w_n) + {{\mathcal {L}}}_n \Pi _n^\bot {{\mathcal {L}}}_n^{- 1} \Pi _n {{\mathcal {F}}}(w_n) + Q_n \\&= \Pi _n^\bot {{\mathcal {F}}}(w_n) + [{{\mathcal {L}}}_n, \Pi _n^\bot ]{{\mathcal {L}}}_n^{- 1} \Pi _n {{\mathcal {F}}}(w_n) + Q_n . \end{aligned} \end{aligned}$$

We estimate separately the three terms in (7.18). Note that, by (7.4), we have $\gamma ^{- 1} = {\mathtt N}_{0} \leqslant {\mathtt N}_{n}$. By (7.1), Lemma 3.1-(*i*), (7.5) and (7.4), we have

7.19 $$\begin{aligned} \begin{aligned}&\Vert \Pi _n^\bot {{\mathcal {F}}}(w_n) \Vert _{s_0} \leqslant {\mathtt N}_{n}^{- \mathtt b_1} \Vert {{\mathcal {F}}}(w_n) \Vert _{s_0 + \mathtt b_1 } , \quad \Vert \Pi _n^\bot {{\mathcal {F}}}(w_n) \Vert _{s_0+{\mathtt b}_{1}} \leqslant \Vert {\mathcal F}(w_n) \Vert _{s_0 + \mathtt b_1 } . \end{aligned} \end{aligned}$$

By (7.5), Lemma 3.1-(*i*),(*ii*), (7.1), (7.11), (7.4), (7.8), (7.14), we have

7.20 $$\begin{aligned}&\Vert [{{\mathcal {L}}}_n, \Pi _n^\bot ] {{\mathcal {L}}}_n^{- 1} \Pi _n {{\mathcal {F}}}(w_n) \Vert _{s_0} \nonumber \\&\quad \lesssim \lambda ^{\theta } \,{\mathtt N}_{n}^{1-{\mathtt b}_{1}}\Big ( \Vert {\mathcal {L}}_{n}^{- 1} \Pi _n {\mathcal {F}}(w_n) \Vert _{s_0 + {\mathtt b}_{1}} + \Vert w_n \Vert _{s_0 + {\mathtt b}_{1}} \Vert {\mathcal {L}}_{n}^{- 1} \Pi _n {\mathcal {F}}(w_n) \Vert _{s_0 + 1} \Big ) \nonumber \\&\quad \lesssim \lambda ^{\theta -1}\gamma ^{-1} \,{\mathtt N}_{n}^{1- {\mathtt b}_{1}} \Big ( \Vert \Pi _n {\mathcal {F}}(w_n) \Vert _{s_0 + {\mathtt b}_{1}+{\overline{\sigma }}} +{\mathtt N}_{n} \Vert w_n \Vert _{s_0 + {\mathtt b}_{1}+{\overline{\sigma }}} \Vert \Pi _n {\mathcal {F}}(w_n) \Vert _{s_0 + {\overline{\sigma }}} \Big ) \nonumber \\&\quad \lesssim \lambda ^{\theta -1}\,{\mathtt N}_{n}^{2- {\mathtt b}_{1}} \Big ( {\mathtt N}_{n}^{{\overline{\sigma }}} \Vert {\mathcal {F}}(w_n) \Vert _{s_0 + {\mathtt b}_{1}} +{\mathtt N}_{n}^{1+2{\overline{\sigma }}} \Vert w_n \Vert _{s_0 + {\mathtt b}_{1}} \Vert {\mathcal {F}}(w_n) \Vert _{s_0} \Big ) \nonumber \\&\quad \lesssim \lambda ^{\theta -1}\,{\mathtt N}_{n}^{2{\overline{\sigma }} +3- {\mathtt b}_{1}} \Big ( \Vert {\mathcal {F}}(w_n) \Vert _{s_0 + {\mathtt b}_{1}} + C_* {\mathtt N}_{n-1}^{-{\mathtt a}_{1}} \Vert w_n \Vert _{s_0 + {\mathtt b}_{1}} \Big ) \nonumber \\&\quad \lesssim \lambda ^{\theta -1}\,{\mathtt N}_{n}^{2{\overline{\sigma }} +3- {\mathtt b}_{1}} \Big ( \Vert {\mathcal {F}}(w_n) \Vert _{s_0 + {\mathtt b}_{1}} + \Vert w_n \Vert _{s_0 + {\mathtt b}_{1}} \Big ) , \end{aligned}$$

7.21 $$\begin{aligned}&\Vert [{{\mathcal {L}}}_n, \Pi _n^\bot ] {{\mathcal {L}}}_n^{- 1} \Pi _n {{\mathcal {F}}}(w_n) \Vert _{s_0+{\mathtt b}_{1}} = \Vert [\Pi _n,{\mathcal {L}}_{n}] {{\mathcal {L}}}_n^{- 1} \Pi _n {{\mathcal {F}}}(w_n) \Vert _{s_0+{\mathtt b}_{1}} \nonumber \\&\quad \lesssim \lambda ^{\theta } \,{\mathtt N}_{n} \Big ( \Vert {\mathcal {L}}_{n}^{- 1} \Pi _n {\mathcal {F}}(w_n) \Vert _{s_0 + {\mathtt b}_{1}} + \Vert w_n \Vert _{s_0 + {\mathtt b}_{1}+1} \Vert {\mathcal {L}}_{n}^{- 1} \Pi _n {\mathcal {F}}(w_n) \Vert _{s_0 + 1} \Big ) \nonumber \\&\quad \lesssim \lambda ^{\theta -1}\gamma ^{-1} \,{\mathtt N}_{n}  \Big ( \Vert \Pi _n {\mathcal {F}}(w_n) \Vert _{s_0 + {\mathtt b}_{1}+{\overline{\sigma }}} +{\mathtt N}_{n} \Vert w_n \Vert _{s_0 + {\mathtt b}_{1}+{\overline{\sigma }}} \Vert \Pi _n {\mathcal {F}}(w_n) \Vert _{s_0 + {\overline{\sigma }}} \Big ) \nonumber \\&\quad \lesssim \lambda ^{\theta -1}\,{\mathtt N}_{n}^{2} \Big ( {\mathtt N}_{n}^{{\overline{\sigma }}} \Vert {\mathcal {F}}(w_n) \Vert _{s_0 + {\mathtt b}_{1}} +{\mathtt N}_{n}^{1+2{\overline{\sigma }}} \Vert w_n \Vert _{s_0 + {\mathtt b}_{1}} \Vert {\mathcal {F}}(w_n) \Vert _{s_0} \Big ) \nonumber \\&\quad \lesssim \lambda ^{\theta -1}\,{\mathtt N}_{n}^{2{\overline{\sigma }} +3} \Big ( \Vert {\mathcal {F}}(w_n) \Vert _{s_0 + {\mathtt b}_{1}} + C_* {\mathtt N}_{n-1}^{-{\mathtt a}_{1}} \Vert w_n \Vert _{s_0 + {\mathtt b}_{1}} \Big ) \nonumber \\&\quad \lesssim \lambda ^{\theta -1}\,{\mathtt N}_{n}^{2{\overline{\sigma }} +3} \Big ( \Vert {\mathcal {F}}(w_n) \Vert _{s_0 + {\mathtt b}_{1}} + \Vert w_n \Vert _{s_0 + {\mathtt b}_{1}} \Big ). \end{aligned}$$

By Lemma 3.1-(*iii*), (7.1), (7.5), (7.15) and (7.4), (7.16), (7.8), using also that $\lambda ^{\theta -1}=\lambda ^{\alpha -2+{\mathtt c}}<1$ for $\lambda \gg 1$, we have

7.22 $$\begin{aligned} \begin{aligned} \Vert Q_n \Vert _{s_0}&\lesssim \lambda ^{\theta } {\mathtt N}_{n}^2 \Vert h_{n + 1} \Vert _{s_0}^2 \lesssim \lambda ^{\theta } {\mathtt N}_{n}^{2 {\overline{\sigma }} + 2} \lambda ^{- 2} \gamma ^{- 2} \Vert {{\mathcal {F}}}(w_n) \Vert _{s_0}^2 \\&\lesssim \lambda ^{\theta -2} {\mathtt N}_{n}^{2 {\overline{\sigma }} + 4} \Vert {{\mathcal {F}}}(w_n) \Vert _{s_0}^2 \lesssim {\mathtt N}_{n}^{2 {\overline{\sigma }} + 4} \lambda ^{- 1} \Vert {{\mathcal {F}}}(w_n) \Vert _{s_0}^2 , \\ \Vert Q_n \Vert _{s_0+{\mathtt b}_{1}}&\lesssim \lambda ^{\theta } \Vert h_{n+1}\Vert _{s_0+{\mathtt b}_{1}+1} \Vert h_{n+1} \Vert _{s_0+1} \lesssim \lambda ^{\theta } {\mathtt N}_{n}^{2} \Vert h_{n+1}\Vert _{s_0+{\mathtt b}_{1}} \Vert h_{n+1} \Vert _{s_0} \\&\lesssim \lambda ^{\theta -1}\gamma ^{-1} {\mathtt N}_{n}^{4{\overline{\sigma }}+3} \Big ( \Vert {\mathcal {F}}(w_n) \Vert _{s_0+{\mathtt b}_{1}} + \Vert w_{n} \Vert _{s_0+{\mathtt b}_{1}} \Big ) \Vert {\mathcal {F}}(w_n) \Vert _{s_0} \\&\lesssim \lambda ^{\theta -1}\gamma ^{-1} {\mathtt N}_{n}^{4{\overline{\sigma }}+3} \Big ( \Vert {\mathcal {F}}(w_n) \Vert _{s_0+{\mathtt b}_{1}} + \Vert w_{n} \Vert _{s_0+{\mathtt b}_{1}} \Big ) C_{*} {\mathtt N}_{n-1}^{-{\mathtt a}_{1}} \\&\lesssim \ {\mathtt N}_{n}^{4{\overline{\sigma }}+4} \Big ( \Vert {\mathcal {F}}(w_n) \Vert _{s_0+{\mathtt b}_{1}} + \Vert w_{n} \Vert _{s_0+{\mathtt b}_{1}} \Big ) . \end{aligned} \end{aligned}$$

Therefore, by (7.17) and estimating (7.18) with (7.19), (7.20), (7.21), (7.22), we have proved the following inductive inequalities for any $n \geqslant 0$ and on the set ${{\mathcal {G}}}_{n + 1}$:

7.23 $$\begin{aligned} \begin{aligned}&\Vert w_{n + 1} \Vert _{s_0 + \mathtt b_1} + \Vert {\mathcal {F}}(w_{n+1})\Vert _{s_0+{\mathtt b}_{1}} \lesssim {\mathtt N}_{n}^{4 {\overline{\sigma }} + 4} \Big ( \Vert {{\mathcal {F}}}(w_n) \Vert _{s_0 + \mathtt b_1} + \Vert w_n \Vert _{s_0 + \mathtt b_1 } \Big ), \\&\quad \Vert {\mathcal {F}}(w_{n+1}) \Vert _{s_0} \lesssim {\mathtt N}_{n}^{2 {\overline{\sigma }} + 3 - \mathtt b_1} \Big ( \Vert {{\mathcal {F}}}(w_{n}) \Vert _{s_0 + \mathtt b_1} + \Vert w_n \Vert _{s_0 + \mathtt b_1 } \Big )\ + {\mathtt N}_{n}^{2 {\overline{\sigma }} + 4} \lambda ^{- 1} \Vert {{\mathcal {F}}}(w_n) \Vert _{s_0}^2. \end{aligned} \end{aligned}$$

**Proof of** $({{\mathcal {P}}}1)_{n + 1}$. By (7.15), (7.8), (7.4) and (7.2), we obtain

$$\begin{aligned} \Vert h_{n+1} \Vert _{s_0+{\overline{\sigma }}} \lesssim {\mathtt N}_{n}^{2{\overline{\sigma }}} {\mathtt N}_{n-1}^{-{\mathtt a}_{1}} \gamma ^{-1} \lambda ^{-1} \lesssim {\mathtt N}_{n}^{2{\overline{\sigma }}+1} {\mathtt N}_{n-1}^{-{\mathtt a}_{1}} \lambda ^{-1}, \end{aligned}$$

which proves (7.6) at the step $n+1$. By (7.10), by the definition of the constants in (7.2) and by the smallness condition in (7.4), the estimate (7.5) at the step $n + 1$ follows since

$$\begin{aligned} \begin{aligned} \Vert w_{n + 1} \Vert _{s_0 + {\overline{\sigma }}} \leqslant \Vert w_0 \Vert _{s_0 + \overline{\sigma }} + \sum _{k = 1}^{n + 1} \Vert h_k \Vert _{s_0 + {\overline{\sigma }}} \leqslant \tfrac{1}{2} C_0 + C_*\sum _{k = 1}^\infty {\mathtt N}_{k-1}^{2 {\overline{\sigma }}} {\mathtt N}_{k -2}^{-\mathtt a_1} \lambda ^{- 1} \leqslant C_0, \end{aligned} \end{aligned}$$

for ${\mathtt N}_{0}=\gamma ^{-1}=\lambda ^{\mathtt c}\gg 1$ large enough and, if needed, $C_0>1$ a bit larger. We prove now that $w_{n+1}$ is a quasi-periodic traveling wave. By induction, we have that $w_{n}$ is a quasi-periodic. Moreover, the function $h_{n+1}$ in (7.13) is a quasi-periodic traveling wave by Proposition 6.2, Lemma 3.1, Lemma 2.12, and the definition of the projections only on momentum preserving sites, see (2.6). Therefore, by (7.13), $w_{n+1}:=w_n + h_{n+1}$ is a quasi-periodic traveling wave. Moreover, in the case where the forcing term *f* in (3.1) is $\textrm{even}({\varphi }, x)$, if by induction hypothesis $w_n = \textrm{odd}({\varphi }, x)$, then ${{\mathcal {F}}}(w_n) = \textrm{even}({\varphi }, x)$, ${{\mathcal {L}}}_n^{- 1}$ is reversibile, implying that $h_{n + 1} = \textrm{odd}({\varphi }, x)$ and hence also $w_{n + 1} = w_n + h_{n + 1} = \textrm{odd}({\varphi }, x)$.

**Proof of** $({{\mathcal {P}}}2)_{n + 1}, ({{\mathcal {P}}}3)_{n + 1}$. By the induction estimate (7.23) and using the induction hypothesis on $({{\mathcal {P}}}2)_{n }, ({{\mathcal {P}}}3)_{n }$, we obtain that

$$\begin{aligned} \begin{aligned} \Vert w_{n + 1} \Vert _{s_0 + \mathtt b_1} + \Vert {\mathcal {F}}(w_{n+1})\Vert _{s_0+{\mathtt b}_{1}}&\leqslant C\, {\mathtt N}_{n}^{4 {\overline{\sigma }} + 4} \Big ( \Vert {{\mathcal {F}}}(w_n) \Vert _{s_0 + \mathtt b_1} + \Vert w_n \Vert _{s_0 + \mathtt b_1 } \Big ) \\&\leqslant C C_* {\mathtt N}_{n}^{4{\overline{\sigma }} +4} {\mathtt N}_{n-1}^{\kappa } \leqslant C_* {\mathtt N}_{n}^{\kappa }, \end{aligned} \end{aligned}$$

by the choice of the constant $\kappa$ in (7.2) and taking ${\mathtt N}_{0} \gg 1$ large enough. This latter chain of inequalities proves $({{\mathcal {P}}}3)_{n + 1}$. Moreover, by (7.23), by the smallness condition (7.4) and by the choice of the constants in (7.2), we have

$$\begin{aligned} \begin{aligned} \Vert {\mathcal {F}}(w_{n+1}) \Vert _{s_0}&\leqslant C\, {\mathtt N}_{n}^{2 {\overline{\sigma }} + 3 - \mathtt b_1} \Big ( \Vert {{\mathcal {F}}}(w_n) \Vert _{s_0 + \mathtt b_1} + \Vert w_n \Vert _{s_0 + \mathtt b_1 } \Big )\ + C\,{\mathtt N}_{n}^{2 {\overline{\sigma }} + 4} \lambda ^{- 1} \Vert {{\mathcal {F}}}(w_n) \Vert _{s_0}^2 \\&\leqslant C C_* {\mathtt N}_{n}^{2 {\overline{\sigma }} + 3 - \mathtt b_1} {\mathtt N}_{n-1}^{\kappa } + C C_* {\mathtt N}_{n}^{2 {\overline{\sigma }} + 4} {\mathtt N}_{n-1}^{-2{\mathtt a}_{1}} \lambda ^{- 1} \\&\leqslant C_* {\mathtt N}_{n}^{-{\mathtt a}_{1}} . \end{aligned} \end{aligned}$$

The proof of the claimed statement is then concluded. $\square$

## Proof of Theorem 1.3 and Measure Estimates

Fix $\gamma = \lambda ^{- \mathtt c}$ as in (7.2), (7.4), in particular with $0<{ \mathtt c} < {\overline{\tau }}\,^{-1}$. Then we have that

$$\begin{aligned} {\mathtt N}_{0}^{{\overline{\tau }}} \lambda ^{- 1} = \gamma ^{- {\overline{\tau }}} \lambda ^{- 1} = \lambda ^{\mathtt c {\overline{\tau }} - 1} \ll 1 \end{aligned}$$

by taking $\lambda \gg 0$ large enough, since $1 - \mathtt c \overline{\tau }> 0$. This implies that the smallness condition (7.4) is fullfilled. We define the set ${{\mathcal {G}}}_\infty$ as

8.1 $$\begin{aligned} {{\mathcal {G}}}_\infty := \bigcap _{n \geqslant 0} {{\mathcal {G}}}_n \end{aligned}$$

where the sets ${{\mathcal {G}}}_n$ are given in Proposition 7.3-$({{\mathcal {P}}}1)_n$. Hence, by Proposition 7.3-$({{\mathcal {P}}}1)_n$, using a telescoping argument, for any $\omega \in {\mathcal G}_\infty$, the sequence $(w_n)_{n \geqslant 0}$ converges to $w_\infty \in H^{s_0 + {\overline{\sigma }}}_0$ with respect to the norm $\Vert \cdot \Vert _{s_0 + {\overline{\sigma }}}^{\textrm{Lip}(\gamma )}$, and

8.2 $$\begin{aligned} \Vert w_\infty \Vert _{s_0 + {\overline{\sigma }}}^{\textrm{Lip}(\gamma )} \leqslant C_0, \quad \Vert w_\infty - w_n \Vert _{s_0 + {\overline{\sigma }}}^{\textrm{Lip}(\gamma )} \lesssim {\mathtt N}_{n}^{2 {\overline{\sigma }}} {\mathtt N}_{n- 1}^{-\mathtt a_1} \lambda ^{- 1} \gamma ^{-1}, \quad \forall \,n \geqslant 1, \end{aligned}$$

and consequently, by the same arguments, recalling (7.3),

8.3 $$\begin{aligned} \begin{aligned} \inf _{\omega \in {{\mathcal {G}}}_\infty }\Vert w_{\infty }(\cdot ; \omega ) \Vert _{s_0+{\overline{\sigma }}}&\geqslant \inf _{\omega \in {{\mathcal {G}}}_\infty } \Vert w_{0}(\cdot ; \omega ) \Vert _{s_0+{\overline{\sigma }}} - \Vert w_{\infty } -w_0 \Vert _{s_0+{\overline{\sigma }}}^{\textrm{Lip}(\gamma )} \\&\geqslant K \lambda ^{-{\mathtt c}} - C_* {\mathtt N}_{0}^{2{\overline{\sigma }}} \lambda ^{-1}\gamma ^{-1} \geqslant \tfrac{K}{2} \lambda ^{-{\mathtt c}} , \end{aligned} \end{aligned}$$

for $\lambda \gg 1$ large enough, by (7.2), (7.4) and $\gamma =\lambda ^{-{\mathtt c}}$. By (8.1) and Proposition 7.3-$({{\mathcal {P}}}2)_n$, for any $\omega \in {{\mathcal {G}}}_\infty$, we have ${{\mathcal {F}}}(w_n) \rightarrow 0$ as $n \rightarrow \infty$. Therefore, the estimate (8.2) implies that ${{\mathcal {F}}}(w_\infty ) = 0$ for any $\omega \in {{\mathcal {G}}}_\infty$, whereas estimates (8.2)-(8.3) imply (1.12), after scaling back to $v_{\lambda }:= \lambda ^{\theta } w_{\infty }=\lambda ^{\alpha -1+{\mathtt c}} w_{\infty }$ (recall (4.5)). If we assume that *f* in (3.1) is $\textrm{even}({\varphi }, x)$, then $w_n = \textrm{odd}({\varphi }, x)$ for any $n \geqslant 0$ and hence also $w_\infty = \textrm{odd}({\varphi }, x)$. The linearized equation at the quasi-periodic traveling wave solution $v_\lambda (\lambda \, \omega t, x) = \lambda ^\theta w_\infty (\lambda \, \omega t, x)$, obtained by linearizing (1.7), has the form

$$\begin{aligned} \begin{aligned}&\partial _t h + L(\lambda \, \omega t)[h ] = 0, \quad L({\varphi }):= \textbf{a}_0({\varphi }, x) \cdot \nabla + {{\mathcal {E}}}_0({\varphi }) , \\&\quad \textbf{a}_{0}({\varphi }, x):= {\mathfrak {B}}\big [ v_\lambda ]({\varphi }, x) , \quad {\mathcal {E}}_{0}({\varphi })[h]:= \nabla v_\lambda ({\varphi }, x) \cdot {\mathfrak {B}}[h], \quad {\mathfrak {B}}= \nabla ^\perp (-\Delta )^{-1} . \end{aligned} \end{aligned}$$

The linearized equation can be fully reduced to the constant coefficients equation

$$\begin{aligned} \partial _t \phi + \textbf{D}_\infty \phi = 0 \end{aligned}$$

by the normal form scheme that we implemented in Sect. 5, see Propositions 5.5, 5.6, 5.13. In particular, since $w_\infty = \textrm{odd}({\varphi }, x)$, we can apply Lemma 5.11 and we deduce that the eigenvalues of the reduced diagonal operator $\textbf{D}_\infty$ are purely imaginary. Consequently, we conclude that the quasi-periodic traveling wave solution $v_\lambda = \lambda ^\theta w_\infty$ is linearly stable in the reversible case, and, for any $s \geqslant 0$, one has $\Vert \phi (t) \Vert _{H^s_x} = \Vert \phi (0) \Vert _{H^s_x}$ for any $t \in {\mathbb {R}}$ and hence $\Vert h(t) \Vert _{H^s_x} \lesssim _s \Vert h(0 ) \Vert _{H^s_x}$ for any $t \in {\mathbb {R}}$.

By setting $\Omega _\lambda := {{\mathcal {G}}}_\infty$, the proof of Theorem 1.3 is concluded once we estimate the Lebesgue measure of the set $\Omega {\setminus } \Omega _\lambda$, with $\Omega \subset {\mathbb {R}}^\nu$ as in (1.11). This is the content of the last part of the paper.

The main result is the following proposition.

### Proposition 8.1

Let $\Omega \subset {\mathbb {R}}^\nu$ be given as in (1.11) and let

8.4 $$\begin{aligned} \tau := \nu + 4. \end{aligned}$$

Then, we have that $|\Omega {\setminus } {{\mathcal {G}}}_\infty | \lesssim \gamma$. As a consequence, with $\gamma = \lambda ^{- \mathtt c}$ as in (7.2), (7.4), we have that $|\Omega {\setminus } {{\mathcal {G}}}_\infty | \lesssim \lambda ^{-{\mathtt c}}\rightarrow 0$ as $\lambda \rightarrow \infty$.

The rest of this section is devoted to the proof of Proposition 8.1. By (8.1), (7.7), (3.6) and (3.5), we have

8.5 $$\begin{aligned} \Omega \setminus {{\mathcal {G}}}_\infty \subseteq \big ( \Omega \setminus {\mathtt D}{\mathtt C}(\gamma ,\tau ) \big ) \bigcup \big ( {\mathtt D}{\mathtt C}(\gamma ,\tau ) \setminus \Omega _{\gamma } \big ) \bigcup _{n \geqslant 0} ({{\mathcal {G}}}_n \setminus {{\mathcal {G}}}_{n + 1}). \end{aligned}$$

By Lemma 3.2 and the standard estimate $|\Omega \setminus {\mathtt D}{\mathtt C}(\gamma ,\tau )|$, it remains to estimate the measure of ${{\mathcal {G}}}_n \setminus {{\mathcal {G}}}_{n + 1}$. By (7.7) and using elementary properties of set theory, one has that

8.6 $$\begin{aligned} {{\mathcal {G}}}_n \setminus {{\mathcal {G}}}_{n + 1} \subseteq ({\mathcal G}_n \setminus \Omega _\infty ^{\gamma _n}(w_n)) \cup ({{\mathcal {G}}}_n \setminus \Lambda _\infty ^{\gamma _n}(w_n))\, \end{aligned}$$

where we recall that the sets $\Omega _\infty ^{\gamma _n}(w_n)$, $\Lambda _\infty ^{\gamma _n}(w_n)$ are defined in (5.105), (6.3). Note that ${{\mathcal {G}}}_{n+1} \subseteq \mathcal {G}_{0} = \Omega _{\gamma }$ for any $n \geqslant 0$, by (7.7).

### Proposition 8.2

For any $n \geqslant 0$, the following estimates hold:

(*i*) ${{\mathcal {G}}}_0 {\setminus } \Omega _\infty ^{\gamma _0}(w_0) \lesssim \gamma$ and, for any $n \geqslant 1$, $|{{\mathcal {G}}}_n {\setminus } \Omega _\infty ^{\gamma _n}(w_n)| \lesssim \gamma {\mathtt N}_{n}^{- (\tau - 1)}$;

(*ii*) ${{\mathcal {G}}}_0 {\setminus } \Lambda _\infty ^{\gamma _0}(w_0) \lesssim \gamma$ and for any $n \geqslant 1$,$|{{\mathcal {G}}}_n {\setminus } \Lambda _\infty ^{\gamma _n}(w_n)| \lesssim \gamma {\mathtt N}_{n}^{- (\tau - 1)}$.

As a consequence $|{{\mathcal {G}}}_0 {\setminus } {\mathcal G}_1| \lesssim \gamma$ and for any $n \geqslant 1$, $|{{\mathcal {G}}}_n {\setminus } {{\mathcal {G}}}_{n + 1}| \lesssim \gamma {\mathtt N}_{n }^{- (\tau - 1)}$.

Propositions 8.1 and 8.2 are proved at the end of this section. Now we estimate the measure of the set ${{\mathcal {G}}}_n {\setminus } \Omega _\infty ^{\gamma _n}(w_n)$, $n\geqslant 0$. The estimate of the measure of ${{\mathcal {G}}}_n {\setminus } \Lambda _\infty ^{\gamma _n}(v_n)$ can be done arguing similarly (it is actually even easier) and therefore it is omitted here. By the definitions (7.7), (5.105), we get that

8.7 $$\begin{aligned} \begin{aligned}&{{\mathcal {G}}}_n \setminus \Omega _\infty ^{\gamma _n}(w_n) \subseteq \bigcup _{(\ell , j, j') \in {{\mathcal {I}}}}{{\mathcal {R}}}_{\ell j j'}(w_n), \end{aligned} \end{aligned}$$

where

8.8 $$\begin{aligned} {{\mathcal {I}}}:= \big \{ (\ell , j, j') \in ({\mathbb {Z}}^\nu \setminus \{0 \}) \times ({\mathbb {Z}}^2 \setminus \{ 0 \}) \times ({\mathbb {Z}}^2 \setminus \{ 0 \}): \pi ^\top (\ell ) + j - j' = 0 \big \},\nonumber \\ \end{aligned}$$

and

8.9 $$\begin{aligned} {{\mathcal {R}}}_{\ell j j'}(w_n):= \Big \{ \omega \in {{\mathcal {G}}}_n: |\textrm{i}\, \lambda \, \omega \cdot \ell + \mu _\infty (j; w_n) - \mu _\infty (j'; w_n) | < \frac{2 \gamma _n\, \lambda }{\langle \ell \rangle ^\tau |j'|^\tau } \Big \}.\nonumber \\ \end{aligned}$$

In the next lemma, we estimate the measure of the resonant sets ${{\mathcal {R}}}_{\ell j j'}(w_n)$.

### Lemma 8.3

For any $n \geqslant 0$, if ${{\mathcal {R}}}_{\ell j j'}(w_n) \ne \emptyset$, then we have that $|{{\mathcal {R}}}_{\ell j j'}(w_n)| \lesssim \gamma \,\langle \ell \rangle ^{-(\tau + 1)} |j'|^{-\tau }$.

### Proof

Recalling (5.103), (5.109), we write $\mu _\infty (j;\omega ) \equiv \mu _\infty (j; \omega , w_n(\omega ))$, with $(\ell , j, j') \in {{\mathcal {I}}}$ as in (8.8), and we set

$$\begin{aligned} \phi (\omega ):= \textrm{i}\,\lambda \,\omega \cdot \ell + \mu _\infty (j; \omega ) - \mu _\infty (j'; \omega ). \end{aligned}$$

Since $\ell \ne 0$, we write $\omega = \frac{\ell }{|\ell |} s + v$, with $s>0$ and $v \cdot \ell = 0$. We estimate the measure of the set

$$\begin{aligned} \begin{aligned} {{\mathcal {Q}}}&:= \Big \{ s: |\psi (s)| < \frac{2 \gamma _n\, \lambda }{\langle \ell \rangle ^\tau |j'|^\tau }, \ \ \frac{\ell }{|\ell |} \,s + v \in \Omega _{\gamma _n} \Big \} , \end{aligned} \end{aligned}$$

where

$$\begin{aligned} \begin{aligned} \psi (s)&:= \phi \Big ( \tfrac{\ell }{|\ell |} s + v\Big ) = \textrm{i}\,\lambda |\ell | s + \mu _\infty (j; s) - \mu _\infty (j'; s) , \quad \mu _\infty (j; s) \equiv \mu _\infty \Big (j; \tfrac{\ell }{|\ell |} s + v \Big ). \end{aligned} \end{aligned}$$

By Lemma 5.11, we have, for any $j\in {\mathbb {Z}}^2\setminus \{0\}$,

$$\begin{aligned} | \mu _\infty (j; s_1) - \mu _\infty (j; s_2)| \lesssim \gamma ^{- 1}\lambda ^{\alpha - 1+{\mathtt c}} |j|^{- 1} |s_1 - s_2| , \end{aligned}$$

and therefore

$$\begin{aligned} \begin{aligned} | \psi (s_1) - \psi (s_2)|&\geqslant \big ( \lambda |\ell | - C \lambda ^{\alpha - 1+{\mathtt c}} \gamma ^{- 1} \big ) |s_1 - s_2|\geqslant \frac{\lambda |\ell |}{2} |s_1 - s_2| , \end{aligned} \end{aligned}$$

since $\lambda ^{\alpha - 2+{\mathtt c}} \gamma ^{- 1} = \lambda ^{\alpha - 2(1-{\mathtt c})} \ll 1$ for $\lambda \gg 1$ large enough (note that $\alpha - 2(1-{\mathtt c}) < 0$ by (7.4)). This implies that the measure of the set ${{\mathcal {Q}}}$ satisfies

$$\begin{aligned} |{{\mathcal {Q}}}| \lesssim \frac{2 \lambda \gamma _n}{\langle \ell \rangle ^\tau |j'|^\tau } \frac{1}{\lambda |\ell |} \lesssim \frac{\gamma }{\langle \ell \rangle ^{\tau + 1} |j'|^{\tau }}. \end{aligned}$$

By the Fubini theorem, we also get the claimed bound on $|{\mathcal R}_{\ell j j'}(w_n)|$. $\square$

Several resonant sets ${\mathcal {R}}_{\ell j j'}(w_n)$ are actually empty.

### Lemma 8.4

Let $(\ell , j, j') \in {{\mathcal {I}}}$, with $|\ell | \leqslant {\mathtt N}_{n}$ and $\textrm{min}\{ |j|, |j'| \} \geqslant {\mathtt N}_{n}^{\tau }$, $n \geqslant 1$. Then ${{\mathcal {R}}}_{\ell j j'}(w_n) = \emptyset$.

### Proof

Let $\omega \in {{\mathcal {G}}}_n \subseteq {\mathtt D}{\mathtt C}(2\gamma _{n - 1},\tau )$, We have to prove that, if $0 < |\ell | \leqslant {\mathtt N}_{n}$, $\textrm{min}\{ |j|, |j'| \} \geqslant {\mathtt N}_{n}^\tau$ and $\pi ^\top (\ell ) + j - j' = 0$, then one has

8.10 $$\begin{aligned} \begin{aligned}&|\textrm{i}\, \lambda \, \omega \cdot \ell + \mu _\infty (j;w_n) - \mu _\infty (j';w_n)| \geqslant \frac{2\lambda \gamma _{n} }{{\langle {\ell }\rangle }^\tau } \geqslant \frac{2 \lambda \gamma _n}{\langle \ell \rangle ^\tau |j'|^\tau } , \end{aligned} \end{aligned}$$

which implies that ${{\mathcal {R}}}_{\ell j j'}(w_n) = \emptyset$.

By Lemma 5.11, recalling also that $\theta =\alpha -1+{\mathtt c}$ as in (4.5), we have

$$\begin{aligned} \begin{aligned}&|\textrm{i}\, \lambda \, \omega \cdot \ell + \mu _\infty (j;w_n) - \mu _\infty (j';w_n)| \geqslant \lambda |\omega \cdot \ell | - |\mu _{\infty }(j;w_n)| - |\mu _{\infty }(j';w_n)| \\&\quad \geqslant \frac{2\lambda \gamma _{n-1}}{{\langle {\ell }\rangle }^\tau } - \frac{C \lambda ^{\alpha -1+{\mathtt c}}}{\textrm{min}\{ |j|,|j'| \}} \\&\quad \geqslant \frac{2\lambda \gamma _{n}}{{\langle {\ell }\rangle }^\tau } + \frac{2\lambda \big (\gamma _{n - 1} - \gamma _{n} \big ) }{{\langle {\ell }\rangle }^\tau }- \frac{C \lambda ^{\alpha -1+{\mathtt c}}}{ {\mathtt N}_{n}^{\tau } } \geqslant \frac{2\lambda \gamma _{n}}{{\langle {\ell }\rangle }^\tau }, \end{aligned} \end{aligned}$$

provided that, since $\gamma _{n - 1}-\gamma _{n} = \gamma \,2^{-n}$,

$$\begin{aligned} \frac{2\lambda \big (\gamma _{n - 1} - \gamma _{n} \big ) }{{\langle {\ell }\rangle }^\tau }- \frac{C \lambda ^{\alpha -1+{\mathtt c}}}{ {\mathtt N}_{n}^{\tau } } \geqslant \frac{2\lambda \,\gamma \, 2^{-n}}{{\langle {\ell }\rangle }^{\tau }} \Big ( 1 - C \lambda ^{\alpha -2+{\mathtt c}} \gamma ^{-1} 2^{n-1} {\mathtt N}_{n}^{-\tau } {\langle {\ell }\rangle }^\tau \Big ) \geqslant 0 . \end{aligned}$$

This latter condition is satisfied since $|\ell | \leqslant {\mathtt N}_{n}$ and $\lambda ^{\alpha - 2+{\mathtt c}} \gamma ^{- 1} = \lambda ^{\alpha - 2(1-{\mathtt c})} \ll 1$, with $\lambda \gg 1$ large enough (note that $\alpha - 2(1-{\mathtt c})< 0$ by (7.4)) and ${\mathtt N}_{0}\gg 1$ large enough.

The claimed bound (8.10) is then proved. $\square$

When non-empty, the sequence of resonant sets $({\mathcal {R}}_{\ell j j'}(w_n))_{n\in {\mathbb {N}}_0}$ is nested.

### Lemma 8.5

Let $(\ell , j, j') \in {{\mathcal {I}}}$, with $|\ell | \leqslant {\mathtt N}_{n}$ and $\textrm{min}\{ |j|, |j'| \} \leqslant {\mathtt N}_{n}^\tau$, $n \geqslant 1$. Then

8.11 $$\begin{aligned} {{\mathcal {R}}}_{\ell j j'}(w_n) \subseteq {{\mathcal {R}}}_{\ell j j'}(w_{n - 1}). \end{aligned}$$

### Proof

We split the proof in two steps.

Step 1. We claim that, for any $j' \in {\mathbb {Z}}^2 {\setminus } \{ 0 \}$ and for any $\omega \in {{\mathcal {G}}}_n$, we have

8.12 $$\begin{aligned} \sup _{j \in {\mathbb {Z}}^2 \setminus \{ 0 \}}|\mu _\infty (j; w_n) - \mu _\infty (j'; w_{n - 1})| \lesssim {\mathtt N}_{n-1}^{2 {\overline{\sigma }} + 1} {\mathtt N}_{n-2}^{-\mathtt a_1} + {\mathtt N}_{n - 1}^{- {\mathtt a}} \end{aligned}$$

(the constant $\mathtt a$ is defined in (5.56)). By (5.103) and the inductive definition of the sets ${{\mathcal {G}}}_n$ in (7.7), recalling (5.61), (5.105) and Lemma 5.12, we have the inclusion

8.13 $$\begin{aligned} \begin{aligned} {{\mathcal {G}}}_n \subseteq \Omega _n^{\gamma _{n - 1}}(w_{n - 1}) . \end{aligned} \end{aligned}$$

We aim to apply Proposition 5.9-$\mathbf{(S4)_n}$ with $w_1 \equiv w_{n - 1}$, $w_2 \equiv w_n$, $\gamma \equiv \gamma _{n-1}$ and $\rho \equiv \gamma _{n - 1} - \gamma _n = \gamma 2^{-n}$. By (7.6), we have (recall (5.58))

8.14 $$\begin{aligned} \begin{aligned}&K {\mathtt N}_{n - 1}^{\tau + \tau ^2} \lambda ^{\alpha - 2+{\mathtt c}} \Vert w_n - w_{n - 1} \Vert _{s_0 + \Sigma ({\mathtt b})} \\&\quad \leqslant K C_{*} {\mathtt N}_{n - 1}^{\tau + \tau ^2 + 2 {\overline{\sigma }}} {\mathtt N}_{n-2}^{- \mathtt a_1} \, \lambda ^{\alpha - 2+{\mathtt c}} \lambda ^{- 1} \gamma ^{-1} \leqslant \gamma _{n - 1} - \gamma _n , \end{aligned} \end{aligned}$$

for some constant $K > 0$, where we have used that $\gamma ^{- 1} = {\mathtt N}_{0} \leqslant {\mathtt N}_{n - 1}$ for $n \geqslant 1$ and that

$$\begin{aligned} K C_{*} \, 2^n \, {\mathtt N}_{n - 1}^{\tau + \tau ^2 + 2 {\overline{\sigma }} + 2} {\mathtt N}_{n-2}^{- \mathtt a_1} \, \lambda ^{\alpha - 2+{\mathtt c}} \lambda ^{- 1} \leqslant 1 , \quad n \geqslant 1, \end{aligned}$$

recalling that, by (5.56), (7.2) and the smallness condition (7.4), we have ${\overline{\sigma }} > \Sigma ({\mathtt b})$, $\mathtt a_1 > \chi (\tau + \tau ^2 + 2 {\overline{\sigma }} + 2)$ and ${\overline{\tau }} \geqslant \tau + \tau ^2 + 2 {\overline{\sigma }} + 2$. The estimate (8.14) implies that Proposition 5.9-$\mathbf{(S4)_n}$ applies, leading, together with (8.13), to ${{\mathcal {G}}}_n \subseteq \Omega _n^{\gamma _{n - 1}}(w_{n - 1}) \subseteq \Omega _n^{\gamma _{n}}(w_{n })$. Then, we apply the estimate (5.70) of Proposition 5.9-$\mathbf{(S3)_n}$, with $w_1 \equiv w_{n - 1}$, $w_2 \equiv w_n$, $\gamma _1 \equiv \gamma _n$, $\gamma _2 \equiv \gamma _{n - 1}$, and obtain that, for any $\omega \in \mathcal {G}_{n}$,

8.15 $$\begin{aligned} \begin{aligned} \sup _{j \in {\mathbb {Z}}^2 \setminus \{ 0 \}} |\mu _n(j; w_n) - \mu _n(j; w_{n - 1})|&\lesssim \lambda ^{\alpha - 1+{\mathtt c}} \Vert w_n - w_{n - 1} \Vert _{s_0 + {\overline{\sigma }}} \\&\lesssim {\mathtt N}_{n-1}^{2 {\overline{\sigma }} + 1} {\mathtt N}_{n-2}^{-\mathtt a_1} \lambda ^{\alpha - 2+{\mathtt c}}, \end{aligned} \end{aligned}$$

where we have applied in the last inequality the estimate (7.6). Hence, by triangular inequality and by (5.104), (8.15), (7.5), we have, for any $j \in {\mathbb {Z}}^2 {\setminus } \{ 0 \}$,

$$\begin{aligned} \begin{aligned}&|\mu _\infty (j; w_n) - \mu _\infty (j; w_{n - 1})| \leqslant |\mu _n(j;w_n) - \mu _n(j; w_{n - 1}) | \\&\quad +|\mu _\infty (j; w_n) - \mu _n(j; w_{n})| + | \mu _n(j; w_{n - 1}) - \mu _\infty (j; w_{n - 1}) | \\&\quad \lesssim {\mathtt N}_{n-1}^{2 {\overline{\sigma }} + 1} {\mathtt N}_{n-2}^{-\mathtt a_1} \lambda ^{\alpha - 2+{\mathtt c}} + 2 \lambda ^{-(M(\theta -1)+1)} \gamma ^{-(M+1)} {\mathtt N}_{n - 1}^{- \mathtt a} , \end{aligned} \end{aligned}$$

which, by (7.4), implies the claimed estimate (8.12), since $M>\tfrac{1-{\mathtt c}}{2(1-{\mathtt c})-\alpha }$ by (5.56), $\alpha - 2+{\mathtt c}< 0$ and $\lambda \gg 1$ is large enough.

Step 2. Let $\omega \in {{\mathcal {G}}}_n$ and $(\ell , j, j') \in {{\mathcal {I}}}$, with $|\ell | \leqslant {\mathtt N}_{n}$ and $\textrm{min}\{ |j|, |j'| \} \leqslant {\mathtt N}_{n}^\tau$, $n \geqslant 1$. Note that, since $\pi ^\top (\ell ) + j - j' = 0$, we have that $|j - j'| \lesssim |\ell | \lesssim {\mathtt N}_{n}$ and hence

8.16 $$\begin{aligned} \textrm{max}\{ |j|, |j'| \} \lesssim |j - j'| + \textrm{min}\{ |j|, |j'| \} \lesssim {\mathtt N}_{n} + {\mathtt N}_{n}^\tau \lesssim {\mathtt N}_{n}^\tau . \end{aligned}$$

Therefore, by (8.12), using that $\omega \in {{\mathcal {G}}}_n$, we get, for some constant $C > 0$ large enough,

8.17 $$\begin{aligned} \begin{aligned}&|\lambda \, \textrm{i}\, \omega \cdot \ell + \mu _\infty (j; w_n) - \mu _\infty (j'; w_n)| \\&\quad \geqslant |\lambda \,\textrm{i}\, \omega \cdot \ell + \mu _\infty (j; w_{n - 1}) - \mu _\infty (j'; w_{n - 1})| \\&\qquad - |\mu _\infty (j; w_n) - \mu _\infty (j; w_{n - 1})| - |\mu _\infty (j'; w_n) - \mu _\infty (j'; w_{n - 1})| \\&\quad \geqslant \frac{2 \lambda \gamma _{n - 1}}{\langle \ell \rangle ^\tau |j'|^\tau } - C \Big ( {\mathtt N}_{n-1}^{2 {\overline{\sigma }} + 1} {\mathtt N}_{n-2}^{-\mathtt a_1} + {\mathtt N}_{n - 1}^{- \mathtt a} \Big ) \geqslant \frac{2 \lambda \gamma _{n }}{\langle \ell \rangle ^\tau |j'|^\tau } , \end{aligned} \end{aligned}$$

provided

$$\begin{aligned} C \Big ( {\mathtt N}_{n-1}^{2 {\overline{\sigma }} + 1} {\mathtt N}_{n-2}^{-\mathtt a_1} + {\mathtt N}_{n - 1}^{- \mathtt a} \Big ) \leqslant \frac{2 \lambda (\gamma _{n - 1} - \gamma _n)}{\langle \ell \rangle ^\tau |j'|^\tau }. \end{aligned}$$

Using that $|\ell | \leqslant {\mathtt N}_{n}$, the estimate (8.16), $\gamma _{n - 1} - \gamma _n = \gamma 2^{- n}$, $\gamma ^{- 1} = {\mathtt N}_{0} \leqslant {\mathtt N}_{n}$, the latter condition is implied by

8.18 $$\begin{aligned} C' 2^{n - 1} {\mathtt N}_{n}^{\tau + \tau ^2 + 1} \lambda ^{- 1}\Big ( {\mathtt N}_{n-1}^{2 {\overline{\sigma }} + 1} {\mathtt N}_{n-2}^{-\mathtt a_1} + {\mathtt N}_{n - 1}^{- \mathtt a} \Big ) \leqslant 1, \quad n \geqslant 1, \end{aligned}$$

for some constant $C' \geqslant C$. The condition (8.18) is verified by (5.56), (7.2) and the smallness condition (7.4). We conclude then that the claimed inclusion (8.11) follows by the estimate (8.17). $\square$

### Lemma 8.6

For any $n \geqslant 1$, the following inclusion holds:

8.19 $$\begin{aligned} {{\mathcal {G}}}_n \setminus \Omega _\infty ^{\gamma _n}(w_n) \subseteq \bigcup _{{\begin{array}{c}(\ell , j, j') \in {{\mathcal {I}}} \\ |\ell | \geqslant {\mathtt N}_{n} \end{array}}}{{\mathcal {R}}}_{\ell j j'}(w_n). \end{aligned}$$

### Proof

Let $n \geqslant 1$. By the definition (8.9), ${{\mathcal {R}}}_{\ell j j'}(w_n) \subseteq {{\mathcal {G}}}_n$. By Lemmata 8.4, 8.5, for any $(\ell , j, j') \in {{\mathcal {I}}}$, $|\ell | \leqslant {\mathtt N}_{n}$, if $\textrm{min}\{ |j|, |j'| \} \geqslant {\mathtt N}_{n}^\tau$, then ${{\mathcal {R}}}_{\ell , j j'}(w_n) = \emptyset$, and, if $\textrm{min}\{ |j|, |j'| \} \leqslant {\mathtt N}_{n}^\tau$, then ${{\mathcal {R}}}_{\ell j j'}(w_n) \subseteq {{\mathcal {R}}}_{\ell j j'}(w_{n - 1})$. On the other hand, by (7.7), we have ${{\mathcal {G}}}_n \cap {{\mathcal {R}}}_{\ell j j'}(w_{n - 1}) = \emptyset$. Therefore (8.19) holds. $\square$

Proof of Proposition 8.2. We prove item (*i*). For $(\ell , j, j') \in {{\mathcal {I}}}$, one has that $\pi ^\top (\ell ) + j - j' = 0$ and hence

8.20 $$\begin{aligned} |j| \leqslant |\pi ^\top (\ell )| + |j'| \lesssim |\ell | + |j'| \lesssim \langle \ell \rangle |j'|. \end{aligned}$$

Let $n \geqslant 1$. By Lemmata  8.6, 8.3, the inclusion (8.19), (8.20) and (8.4), we have

$$\begin{aligned} \begin{aligned} \Big | {{\mathcal {G}}}_n \setminus \Omega _\infty ^{\gamma _n}(w_n)\Big |&\lesssim \sum _{(\ell , j, j') \in {{\mathcal {I}}} \atop |\ell | \geqslant {\mathtt N}_{n} } |{{\mathcal {R}}}_{\ell j j'}(w_n)| \lesssim \gamma \sum _{(\ell , j, j') \in {{\mathcal {I}}} \atop |\ell | \geqslant {\mathtt N}_{n} }\frac{1}{\langle \ell \rangle ^{\tau + 1} |j'|^\tau } \\&\lesssim \gamma \sum _{ |\ell | \geqslant {\mathtt N}_{n}} \frac{1}{\langle \ell \rangle ^{\tau - 1}} \sum _{j' \in {\mathbb {Z}}^2 \setminus \{ 0 \}} \frac{1}{|j'|^{\tau - 2}} \lesssim \gamma \, {\mathtt N}_{n}^{- (\tau - 1)}. \end{aligned} \end{aligned}$$

Similarly, by the inclusion (8.7), Lemma 8.3 and using the standard volume estimate $|\Omega {\setminus } {\mathtt D}{\mathtt C}(\gamma , \tau )| \lesssim \gamma$, one also prove that $|{{\mathcal {G}}}_0 {\setminus } \Omega _\infty ^{\gamma _0}(w_0)| \lesssim \gamma$. The item (*ii*) can be proved by similar arguments. Finally the estimate on ${\mathcal G}_n {\setminus } {{\mathcal {G}}}_{n + 1}$ follows by recalling formula (8.6) and by applying items (*i*), (*ii*).

### Proof of Proposition 8.1

It follows by applying Proposition 8.2, Lemma 3.2, using the inclusion (8.5), the standard estimate $|\Omega \setminus {\mathtt D}{\mathtt C}(\gamma ,\tau ) |\lesssim \gamma$ and the fact that the series $\sum _{n \geqslant 1} {\mathtt N}_{n}^{-(\tau - 1)}$ converges. $\square$

## Data Availability

Data sharing is not applicable to this article as no datasets were generated or analyzed during the current study.
